# Design and Selection of Heterodimerizing Helical Hairpins for Synthetic Biology

**DOI:** 10.1021/acssynbio.3c00231

**Published:** 2023-05-24

**Authors:** Abigail J. Smith, Elise A. Naudin, Caitlin L. Edgell, Emily G. Baker, Bram Mylemans, Laura FitzPatrick, Andrew Herman, Helen M. Rice, David M. Andrews, Natalie Tigue, Derek N. Woolfson, Nigel J. Savery

**Affiliations:** † School of Biochemistry, University of Bristol, Bristol BS8 1TD, U.K.; ‡ School of Chemistry, University of Bristol, Bristol BS8 1TS, U.K.; § BioPharmaceuticals R&D, AstraZeneca, Cambridge CB4 0WG, U.K.; ∥ Flow Cytometry Facility, School of Cellular and Molecular Medicine, University of Bristol, Bristol BS8 1TD, U.K.; ⊥ Oncology R&D, AstraZeneca, Cambridge CB21 6GH, U.K.; # BrisEngBio, School of Chemistry, University of Bristol, Bristol BS8 1TS, U.K.

**Keywords:** coiled coil, in-cell library screening, protein−protein interactions, rational peptide design, synthetic biology

## Abstract

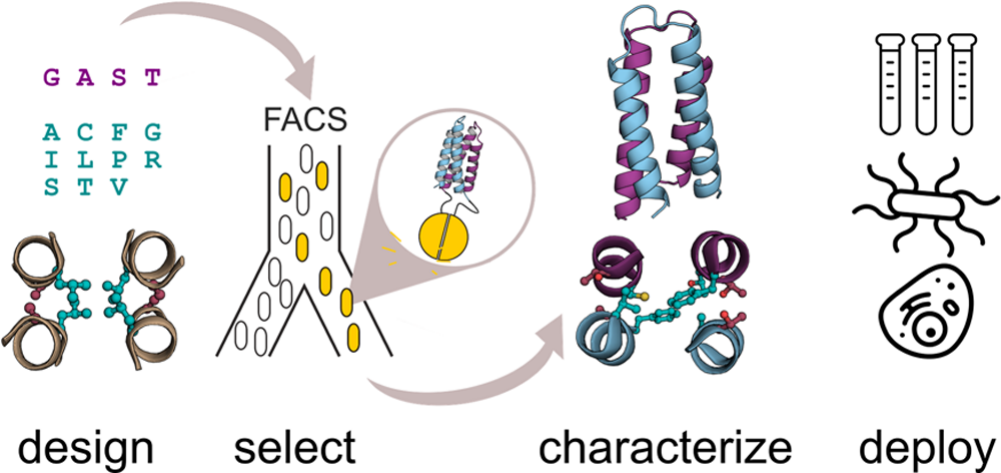

Synthetic biology applications would benefit from protein modules of reduced complexity that function orthogonally to cellular components. As many subcellular processes depend on peptide–protein or protein–protein interactions, *de novo* designed polypeptides that can bring together other proteins controllably are particularly useful. Thanks to established sequence-to-structure relationships, helical bundles provide good starting points for such designs. Typically, however, such designs are tested *in vitro* and function in cells is not guaranteed. Here, we describe the design, characterization, and application of *de novo* helical hairpins that heterodimerize to form 4-helix bundles in cells. Starting from a rationally designed homodimer, we construct a library of helical hairpins and identify complementary pairs using bimolecular fluorescence complementation in *E. coli*. We characterize some of the pairs using biophysics and X-ray crystallography to confirm heterodimeric 4-helix bundles. Finally, we demonstrate the function of an exemplar pair in regulating transcription in both *E. coli* and mammalian cells.

## Introduction

The *de novo* design of peptides and proteins is a rapidly expanding field.^1−3^ This presents opportunities for applying *de novo* proteins to problems of broader interest and utility outside of protein design itself. One application area is synthetic biology. Here, it would be useful to have self-assembling peptides and proteins that are less complex than natural proteins in terms of sequence, structure, function, and the interrelationships between these. In principle, such *de novo* peptides and proteins could be manipulated precisely and predictably at the residue or even the atomic level to tune their functions. In turn, this would allow the precision design and engineering of subcellular processes by manipulating the *de novo* components.

More specifically, because of the significant role of protein–protein interactions (PPIs) in cell biology,^4,5^ an ability to build relatively straightforward, robust, well-understood, and well-characterized *de novo* PPIs would be particularly useful.^6^ For example, these could be deployed to drive interactions between natural proteins in cells, for instance to control enzyme activities and gene regulatory networks.^7−12^ In turn, these could be used as components of cell-based biosensors that can be adapted to respond to different analytes.^8,12,13^ Designed PPIs could also be used to locate and highlight endogenous proteins of interest in cells, and even to alter and control their subcellular location and function.^14−16^ Key properties for synthetic PPI modules include: *simplicity*, they should be as well understood and as straightforward as possible;^17^*specificity*, they should be faithful and not promiscuous, particularly if multiple PPIs are being designed and delivered;^18^*predictability*, they should be transferable with consistent behaviors between cellular systems; and *orthogonality*, their interactions with the endogenous proteomes and PPIs should be minimal unless designed otherwise. Encouragingly, considerable progress is being made in developing *de novo* PPIs to meet these design criteria, and in using the resulting modules to direct biomolecular interactions *in vitro* and in cells.^7,9,10,19−21^

The α-helical coiled coils (CCs) are one class of PPIs that are abundant in nature and have been particularly well characterized.^22^ As a result, there is a wealth of sequence and structural data to derive sequence-to-structure relationships needed for design. In turn, understanding of these relationships has been used to guide the rational or computational design of a wide variety of *de novo* CCs.^1,3,17^ This is because CC sequences have signatures consisting of heptad repeats of hydrophobic (*h*) and polar (*p*) amino acids, *hpphppp*, often denoted *abcdefg*.^17^ In water, such repeats direct the polypeptide chains to fold into amphipathic α helices, and two or more such helices can combine through their hydrophobic interfaces to form helical bundles with left-handed supercoiling. Variation of the hydrophobic residues within the core direct natural and designed CCs to assemble into a variety of oligomerization states and helix orientations.^17^ Dimers, trimers, and tetramers tend to dominate natural CC structures, but through *de novo* design higher-order oligomers can be accessed, as well as uniform homodimers or heterodimers in both parallel or antiparallel orientations.^17,23^ This depth and quality of data and understanding has led to a large number of highly successful CC design programs,^1,17^ and the development of rationally designed “toolkits” of CC peptides.^18,21,24−28^ The application of these toolkits is beginning to realize the vision of building increasingly complex synthetic biological systems from simpler components.^10,20,21,29,30^

One component of the *de novo* peptide toolkit is the tetrameric coiled coil, or 4-helix bundle (4HB). These have been a well-studied target for protein design for several decades due to their apparent simplicity and preponderance in biological systems.^1,3,31^ Moreover, because of their relatively large, well-defined, and malleable hydrophobic cores, 4HBs have proved amenable to modification and the introduction of functions such as metal-ion and small-molecule binding.^1,20,31−33^ 4HBs come in various forms, such as 4-chain tetramers, dimers of helix–loop–helix (hairpin) structures, and single-chain proteins.^28,34^ As discussed later, these present both opportunities and challenges for protein design. Here we target the design and selection of helical hairpins that dimerize to form 4HBs in order to take advantage of the relatively large core for functionalization while keeping a tractable 2-component system.

Specifically, we describe a strategy for selecting cognate heteromeric pairs of *de novo* helical hairpins that dimerize in cells. We start with a rationally designed CC-based hairpin that homodimerizes. From this, we generate a library of hydrophobic-core mutants, which we sample in a split-YFP assay in *E. coli* cells with FACS to identify pairs of heterodimerizing hairpins. From a pool of candidate interacting hairpins, we identify four pairs that are true heterodimeric partners, deselecting the more promiscuous and homodimerizing hairpins. The four selected heterodimeric pairs are characterized thoroughly *in vitro* using solution-phase biophysical methods, and we confirm one pair as a CC-based 4HB by X-ray crystallography. Finally, we show that this fully characterized combination dimerizes as designed and can act as a regulatory PPI in systems that control gene transcription in both bacterial and human cells.

## Results and Discussion

### A Rationally Designed Dimeric Helical Hairpin as a Starting Point for Selection

**Figure 1:**
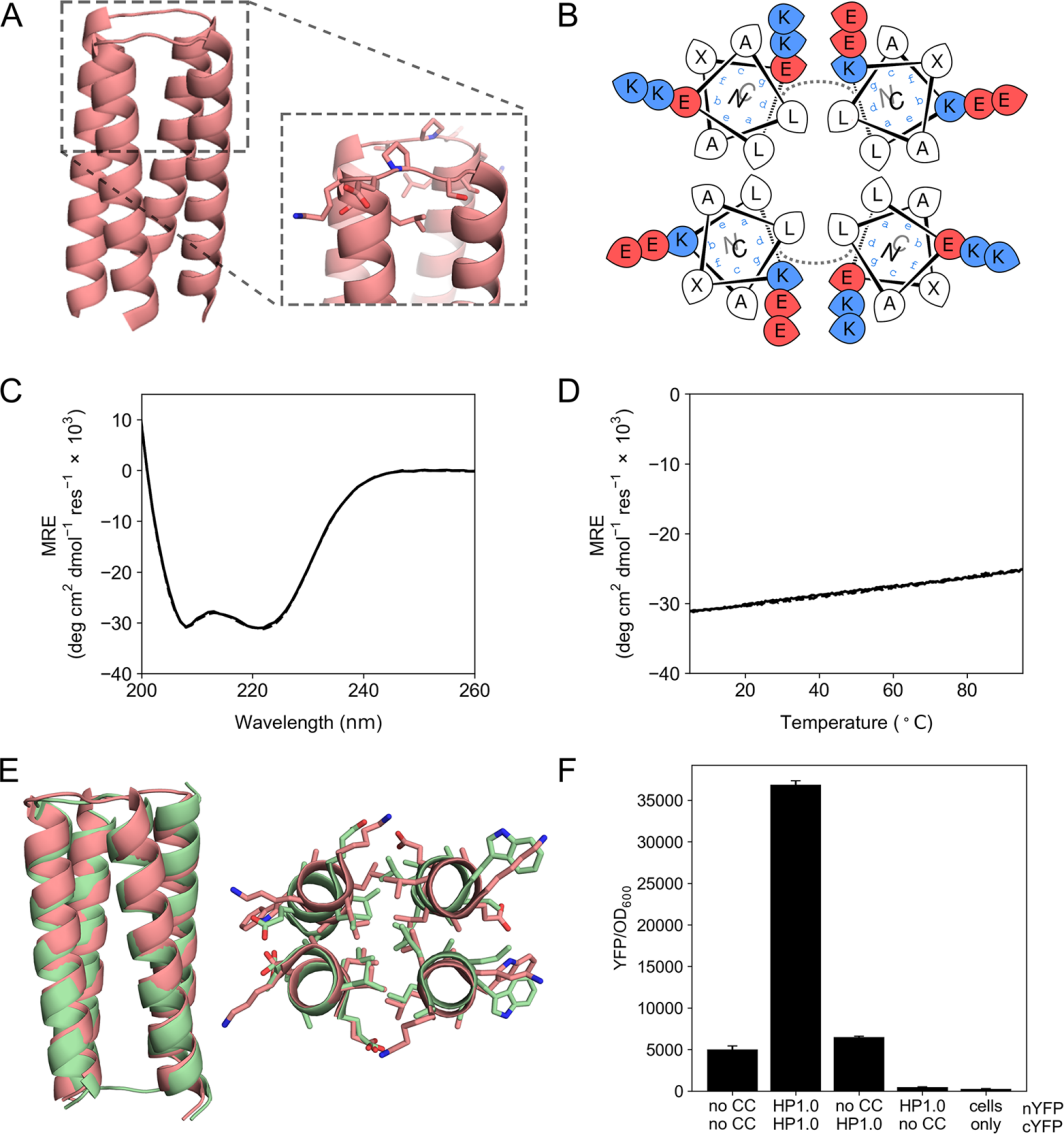
A *de novo* designed CC hairpin that dimerizes to form a 4HB. (A) Model of the designed homodimer CC-HP1.0 in a *syn* topology. This was built by grafting the loop LSKEPS from PDB entry 2fv2 (residues 204–209) onto the structure of apCC-Tet (PDB ID: 6q5s), and then truncating each helix by one heptad. (B) Helical-wheel representation of the *syn* model of CC-HP1.0. (C) CD spectra at 5 °C before (solid line) and after (dashed line) heating to 95 °C, and (D) thermal-response curves of the CD signal at 222 nm (heating, solid line; cooling, dashed line) for peptide CC-HP1.0. Conditions: 10 μM peptide, PBS, pH 7.4. (E) Structural overlay of the X-ray crystal structure of CC-HP1.0 dimer (green; PDB ID, 8bcs; Table S1) and the *in silico* designed *syn* model (pink); RMSD_all-atom_ = 1.035 Å. Left: Backbone overlay of the entire structure. Right: Backbone and side-chain overlay for one heptad repeat. Note: Experimental structure reveals that CC-HP1.0 crystallized in the *anti* conformation (left panel, green cartoon). (F) Bar chart showing the reconstitution of split YFP activity by CC-HP1.0 for combinations of nYFP and cYFP fusions as indicated. YFP fluorescence was normalized to the OD_600_ of the bacterial cell culture, averaged from three different cultures, and shown with standard deviations.

Previously, we and others have successfully used *de novo* designed homo and heterodimeric and tetrameric CCs as PPI modules in *E. coli*, for instance to control transcription when fused to DNA binding domains.^7,9,10,21,28,35^ For the new study presented here, we were interested in (1) breaking the CC symmetry of homomeric assemblies to generate specific heteromers, and (2) screening the hydrophobic-core composition to explore a wider range of CC sequences and, potentially, topologies. As a starting scaffold, we aimed to design a helical hairpin that homodimerized to form a 4HB. This was achieved by adapting the sequence of a previously rationally designed antiparallel homotetramer, apCC-Tet,^36^ and linking two helices to make a hairpin using a loop taken from a natural structure in the RSCB Protein Data Bank (PDB).^37^ The loop was chosen on the basis that it spanned a similar helix-to-helix distance to the target (Figure 1A). There are three possible topologies for the dimeric hairpins: *syn*, with the hairpins side by side and the loops at the same end of the quaternary assembly; *anti*, with the hairpins side by side and the loops at the opposite ends; and a bisecting U, in which the hairpins interdigitate and the loops are at opposite ends of the 4HB (Figure S1).^31,38−40^ We used Glu-Lys-based charge patterning at the *g* and *b* sites of the heptad repeats in an attempt to favor the *syn* topology (Figures 1A,B). The resulting peptide was called CC-HP1.0 (Table 1).

**Table 1:**
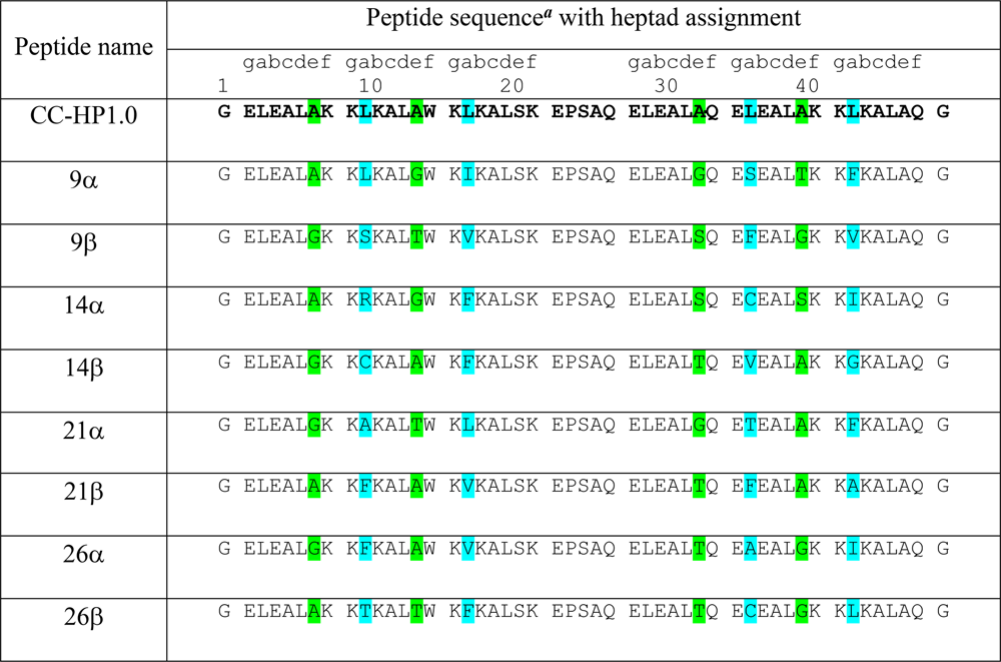
Amino Acid Sequences of the Peptides Designed and Isolated in This Work as Candidate Heterodimerizing Hairpins^a^^,^^b^

a Sequences shown in the table were used for *in vivo* characterization and selection. For *in vitro* characterization, peptides were acetylated at the *N* terminus and amidated at the *C* terminus (see Table S2).

b Cyan and green highlights indicate the *a* and *e* sites, respectively, of the heptad repeats that were varied in the libraries.

CC-HP1.0 was made by solid-phase peptide synthesis (SPPS), purified by HPLC, and confirmed by ESI mass spectrometry (Figure S2). CC-HP1.0 was soluble in aqueous buffer, and circular dichroism (CD) spectroscopy showed that it formed a stable helical assembly without an accessible thermal unfolding transition (Figure 1C,D). By analytical ultracentrifugation (AUC), CC-HP1.0 formed a monodisperse dimer (Figure S2). Moreover, we were able to obtain crystals for the peptide assembly and solve an X-ray crystal structure to 2.1 Å resolution, (Figure 1E and Figure S3; PDB ID, 8bcs). This revealed a dimer of hairpins forming an antiparallel 4-helix core as designed. However, surprisingly, the hairpins were in an *anti*-arrangement rather than *syn*. Nonetheless, the backbone of the computationally designed *syn* model and the experimental structure of the CC-HP1.0 dimer are similar (Figure 1E): they both have wide and narrow helical interfaces;^36^ moreover, the positions of the hydrophobic-core residues overlay very well. Thus, it is possible that, due to the expected similarities of their hydrophobic cores, the *syn* and *anti* forms lie close on the free-energy landscape of these assemblies, and that under the conditions used we have crystallized out the latter.^31,39^ To explore this through modeling, we generated AlphaFold2^59−61^ models using the CC-HP1.0 sequence (Figure S4). These predicted dimers of hairpins with the *syn* arrangement, i.e., as designed but in contrast to the *anti*-arrangement observed in the crystal structure. We return to this point later.

To test whether CC-HP1.0 could be expressed and would form stable dimers in cells, we utilized a bimolecular fluorescence complementation (BiFC) assay in *E. coli*.^41,42^ For this assay, CC-HP1.0 was fused to the *C* termini of the two fragments of split enhanced yellow fluorescence protein (YFP), namely nYFP (residues 1–154) and cYFP (residues 155–238). Both fusions were expressed from plasmids containing the arabinose inducible pBAD promoter. AlphaFold2 predictions of the splitYFP-CC-HP1.0 fusion suggest that both *C* termini of the split-YFP and the linker between cYFP and CC-HP1.0 are likely unstructured (Figure S4). Expression of reconstituted YFP was monitored by measuring the fluorescence of bacterial cultures, normalizing for the OD_600_ of the cell culture (Figure 1F). We observed low levels of fluorescence for the controls in which the YFP fragments were expressed without CC-HP1.0, and controls with a single-CC-HP1.0 on one of the split-YFP fragments. However, with CC-HP1.0 fused to both nYFP and cYFP, YFP activity was 7.4-fold greater than for the split-YFP fragments alone, and 136.4-fold greater than for cells without YFP fusion proteins. Thus, the CC-HP1.0 homodimer appears to reconstitute in *E. coli* cells.

### Heterodimeric Variants of CC-HP1.0 Can Be Selected from Libraries in *E. coli*

With the homodimeric CC-HP1.0 in hand and assembling in *E. coli*, we turned to generating designing heterodimeric variants to deliver new *de novo* PPI modules. While much has been achieved in directing *de novo* CC hetero-oligomerization with electrostatic interactions and charge patterns,^26,43,44^ as observed above, this does not always deliver robust designs. Therefore, we explored how changes in the hydrophobic core might be used instead. To do this, we adapted the split-YFP system to select pairs of heterodimeric hairpins from a library of CC-HP1.0 variants fused to both nYFP and cYFP. We reasoned that substitutions in one hairpin that disrupt the core packing and prevent homodimerization might be compensated for by complementary substitutions in a partner hairpin to generate an obligate heterodimer. Nominally, the core of CC-HP1.0 comprises of Leu residues at the *a* and *d* positions of the CC repeats. However, analysis of the X-ray structure using computational alanine scanning, (BAlaS),^45^ indicated that Leu at *a* sites may contribute more to the hairpin interface of the 4HB (Figure S5). Therefore, we generated a library of variants at these sites, specifically at Leu10, Leu17, Leu36, and Leu43 (Table 1), using the degenerate codon NBC ({A,C,G,T}{C,G,T}C) to give combinations of the amino acids Ala, Cys, Phe, Gly, Ile, Leu, Pro, Arg, Ser, Thr, and Val. In addition, we expanded the library to include some of the adjacent *e* sites, Ala7, Ala14, Ala33, and Ala40, as these also play a role in the interhelix interactions (Figure 1B). However, as the corresponding helix–helix interface at the *e* positions is an Ala-coil with a narrow interface (Figure 1E),^28^ we restricted the amino acid usage to smaller side chains, Gly, Ala, Ser and Thr, using the degenerate codon RST ({A,G}{C,G}T). Thus, each hairpin within the library, named CC-HP1.0-lib, had 8 variable positions, giving a potential library size of ≈3.75 × 10^6^ variants. Therefore, screening two hairpin libraries against each other in the split-YFP assay would give a library with a theoretical complexity of 1.4 × 10^13^. On this basis, a linear combinatorial library was designed, and other codons were optimized for expression in *E. coli*.

**Figure 2:**
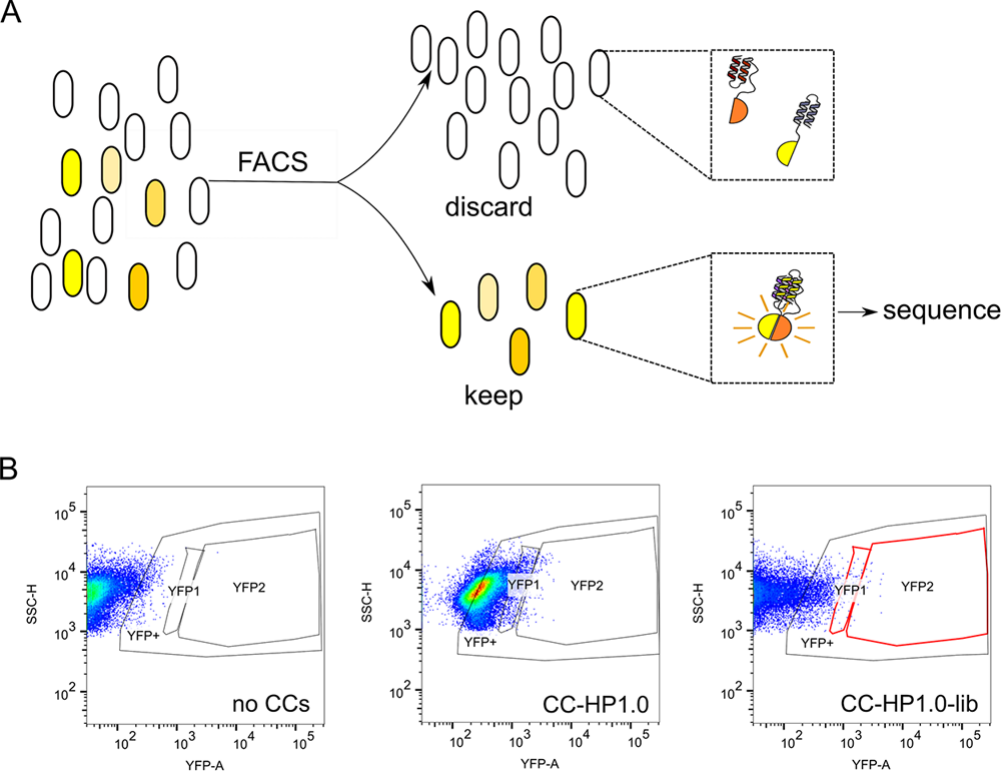
Screening the CC-HP1.0-lib library for heterodimeric hairpins. (A) Cartoon showing the process of library sorting. *E. coli* cells harboring interacting variants of CC-HP1.0 reconstitute split-YFP fluorescence and were separated from cells expressing noninteracting variants by FACS. (B) Representative FACS plots showing *E. coli* cells expressing nYFP and cYFP in the absence of the hairpins (left), fused to CC-HP1.0 (middle), or fused to the degenerate codon library CC-HP1.0-lib (right). Side scatter (SSC) is plotted against YFP fluorescence. A large YFP+ gate was defined as containing cells in which split YFP fluorescence was reconstituted, and the two gates selecting the brightest cells (YFP1 and YFP2), used for sorting potential heterodimers, are outlined in red.

The combinatorial library was cloned *C* terminally to both nYFP and cYFP and *E. coli* were transformed with both libraries. We obtained a library of 1.9 × 10^5^ transformants, i.e., a small subset of the theoretical library complexity. However, even this partial coverage of the library gave multiple heterodimer candidate pairs when screened as follows. Fluorescence-Activated Cell Sorting (FACS) was used to select highly fluorescent “bright” cells, which we assumed had reconstituted split-YFP (Figure 2). Cells expressing nYFP and cYFP fused to CC-HP1.0 were used as a control to determine gates for identifying YFP-positive cells. To select for interacting heterodimers, two gates (YFP1 and YFP2) were set to isolate cells with high and highest YFP fluorescence, respectively (Figure 2B). Single cells were collected from each of these gated populations. These cells were then grown in culture and the DNA encoding the hairpin sequences was amplified by PCR. Reconstitution of split YFP was confirmed by assaying the fluorescence of selected cells in 96 well plates. 27% of the selectants did not exhibit YFP fluorescence equal to or above that of split YFP reconstituted by the CC-HP1.0 control, and these were discounted. Of the remainder, 47% of cells gave PCR fragments of the expected length, and these were sequenced and used for the statistical analysis below.

### Statistical Analysis of Candidate Heterodimer Sequences

**Figure 3:**
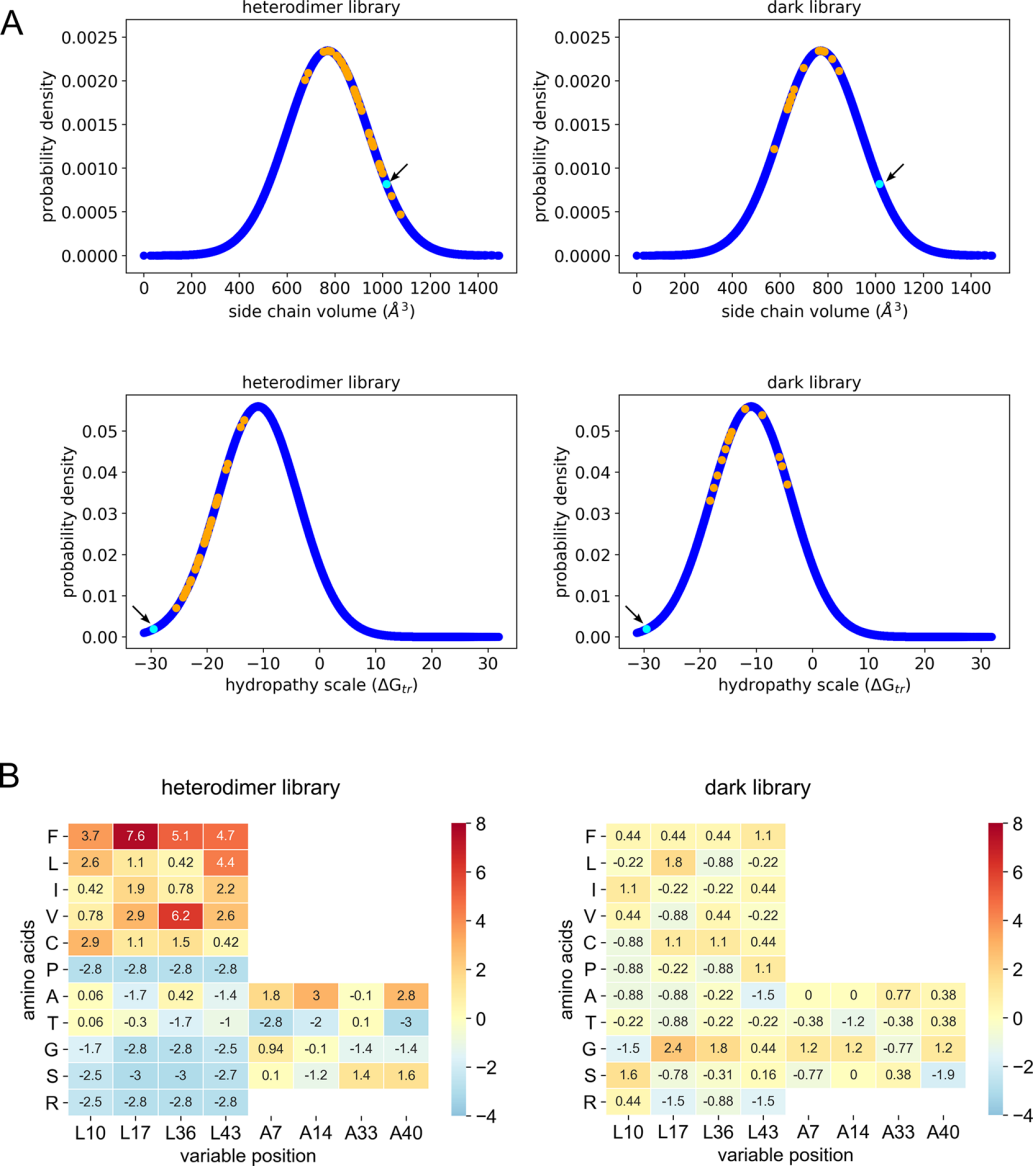
Statistical analysis of interacting hairpins selected from the library. (A) Calculated normal distributions of the summed side-chain volumes and hydrophobicities at the varied *a* and *e* positions for all the possible library variants (blue solid lines). Corresponding values for the sequences of the selected putative heterodimers and the dark library are overlaid on these curves and shown in orange. The values for CC-HP1.0 are plotted in cyan and indicated with an arrow. The values for the heterodimers, dark library, and CC-HP1.0 are shown plotted on the normal-distribution curve to ease comparisons only; however, only the *x*-axis values are relevant for these points. (B) Heatmap showing the *z*-score of the standard error of proportion^48^ of the observed selected amino acids compared to the expected numbers of those amino acids. The amino acids are ordered in increasing hydrophobicity from bottom to top. The *a* sites (positions 10, 17, 36, and 43) are shown left, and the *e* sites (7, 14, 33, and 40) right. A value of 0 indicates the amino acid is found at the same frequency as that expected by chance; >0 indicates favored residues (red scale); and <0 disfavored residues (blue scale).

We analyzed the sequences of 47 full-length interacting pairs selected from the library. Specifically, we summed the volumes^46^ and the hydrophobicities^47^ of the side chains selected at 16 variable positions for each hairpin (orange points, Figure 3A). These data were superimposed on values calculated for all of the combinations of the variable amino acids in the theoretical library, i.e., the distribution expected by chance assuming no bias in the library (blue line, Figure 3A). Also for comparison, we isolated nonfluorescent (“dark”) cells from a hairpin library produced in a different *E. coli* strain; this library behaved similarly to CC-HP1.0-lib in FACS analysis (Figure S6). When sequenced, the majority (83%) of the PCR fragments from these dark cells contained deletions, insertions, frameshifts, or no CC sequences in one or both of the constructs, and we only rescued 14 pairs with full-length sequences from the “dark” cells. Presumably, this reflects that we screened for nonfluorescent cells in this “dark” gate and there are many ways that cells can be nonfluorescent other than containing hairpins that do not interact. The summed side-chain volumes and hydrophobicities for the 14 full-length sequences and the parent sequence (cyan points) are included in Figure 3A.

Comparing the general features of the “bright” and “dark” sequences revealed trends. For instance, the total side-chain volumes of the targeted residues from the selected pairs were predominantly toward the higher end of the normal distribution together with that for the parent sequence (top panels, Figure 3A). In contrast, sequences from “dark” noninteracting constructs had summed side-chain volumes spanning the middle to lower end of the expected distribution. Similarly, residues from the selected heterodimer candidates were also more hydrophobic than those in the noninteracting sequences, as judged by summed hydrophobicity values for each residue (bottom panels, Figure 3A). Total side-chain volumes were also calculated for individual hairpins, and for the *a* and *e* positions separately (Figure S7). These revealed a bias for larger side chains at the *a* position (Figure 3B). Moreover, and interestingly, the spread of summed side-chain volumes for individual hairpins was greater than observed for hairpin pairs (Figure S7). This suggests that the overall volume of the core of the presumed four-helix bundle is optimized in some way and responsible for stable PPIs in our selection.

Next, we checked for specific amino acid preferences at the variable positions in the selected pairs. To do this, we normalized the numbers of each residue observed at each position by those expected from the degenerate codes used for the theoretical library (Figure 3B). We found a strong preference for larger hydrophobic residues at the *a* sites; for example, Phe at positions 10 and 17, Val or Phe at 36, and Phe or Leu at 43. In contrast, Pro (a known helix-breaking residue) and Arg (charged) were selected against at all *a* sites. At the *e* sites, there was a clear preference to maintain the parent Ala residue, with the only alternative being the next-smallest amino acid, Ser, (Figure 3B). By contrast, the profiles constructed from the sequences from the dark library indicated very little preference for hydrophobic amino acids in noninteracting sequences (Figure 3B).

Together, these data indicate clear preferences for large hydrophobic residues at the *a* sites of the sequence repeats of interacting pairs, and for Ala at *e*. Moreover, *en masse*, the selected sequences have total side-chain volumes and overall hydrophobicities more similar to the parent than to the bulk of the original library. These results strongly imply that the in-cell split-YFP assay selects for stably folded and well-defined 4-helix bundles as intended.

### Selection of Obligate and Nonpromiscuous Heterodimeric Pairs

**Figure 4:**
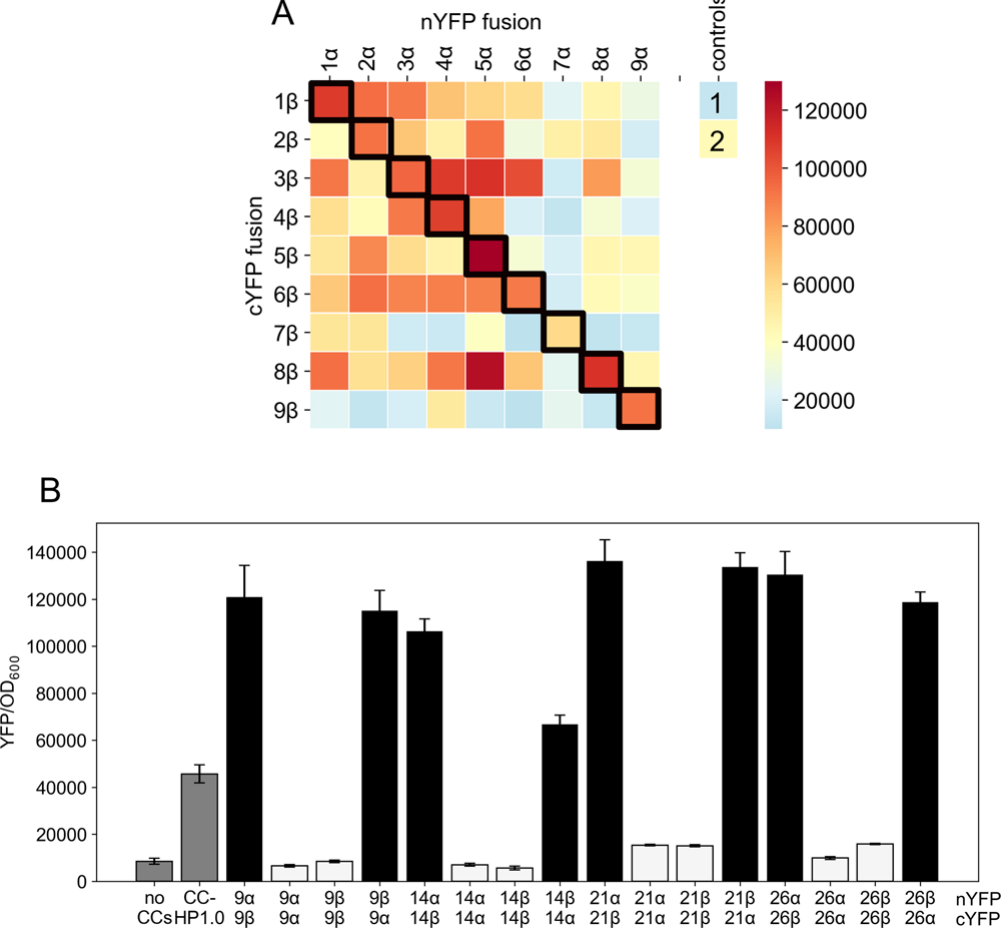
Identifying hairpins from the library screen that form obligate heterodimers. (A) A heatmap showing split-YFP reconstitution between different hairpins fused to nYFP (α) and cYFP (β). The numbering scheme simply refers to pairs isolated from the library screen, with the coselected pairs highlighted by black boxes. Each hairpin was tested against 9 other hairpins. The scale is for YFP fluorescence normalized to the OD_600_ of the bacterial cell culture. Controls are 1, no CCs; and 2, CC-HP1.0. (B) Bar chart of normalized YFP fluorescence for the four selected heterodimer candidates. Each hairpin was fused to nYFP and cYFP in the combinations shown. Bars are colored black to show heterodimer interactions between the α and β hairpins, and white to show homodimerization of two α or two β hairpins. YFP fluorescence was normalized to the OD_600_ of the bacterial cell culture, averaged from three different cultures, and shown with standard deviations.

To identify specific obligate heterodimers, we discarded those hairpins that engaged in promiscuous interactions with hairpins from other selected pairs by carrying out a series of limited all-against-all screens of individual hairpins. To do this, plasmids encoding the nYFP and cYFP hairpin fusions were purified separately; henceforth termed α and β, respectively. Each α hairpin was tested against each β hairpin, using the BiFC assay, in a series of 9 × 9 pairings (Figure 4A and Figure S8A). Combinations that yielded a YFP signal were assumed to form α:β complexes; any hairpins that yielded a YFP signal in association with other hairpins in addition to the original identified cognate pair were deemed promiscuous and unsuitable for generating specific heterodimers. Figure 4A illustrates this: the hairpins from all except the 7α:7β and 9α:9β pairs have significant off-diagonal signals (orange/red coloring). Thus, only pairs 7 and 9 were considered good candidates to take forward. Similarly, from additional limited screens, other pairs (14, 16, 21, 23, 25, 26, and 41 in our numbering scheme) were selected as being the least promiscuous and were taken forward (Figure S8A).

Next, to test for any homodimerization, which we also wanted to avoid, we generated constructs in which the α hairpins were fused to cYFP and the β hairpins to nYFP. This allowed YFP activity to be determined for all homo- and heterodimer combinations for a given pair; i.e., α:β, α:α, β:β, and β:α, (Figure 4B and Figure S8B). Gratifyingly, with the exception of pair 7, the above subset of selected hairpin combinations all formed heterodimers more efficiently than homodimers (Figure S8B). From this analysis, four candidate heterodimeric α:β hairpin combinations were chosen for *in vitro* biophysical and structural studies; namely, pairs 9, 14, 21, and 26. Indeed, the BiFC data for the α:β, α:α, β:β, and β:α combinations for these all had the pattern of YFP reconstitution expected for heterospecific hairpin pairs (Figure 4B). The sequences of these combinations are given in Table 1.

### *In Vitro* Characterization Validates Heterodimerization of the Selected Pairs

We made peptides for the 4 chosen pairs by SPPS. The *N* and *C* termini were acetylated and amidated, respectively. The peptides were purified by HPLC and masses were confirmed by ESI mass spectrometry (Figures S9–S12). Solution-phase biophysical data are illustrated in the main text for the CC-HP1.26α/CC-HP1.26β pair (shorthand 26α/26β; Figure 5) with the complete data given in Figures S14–S25.

**Figure 5:**
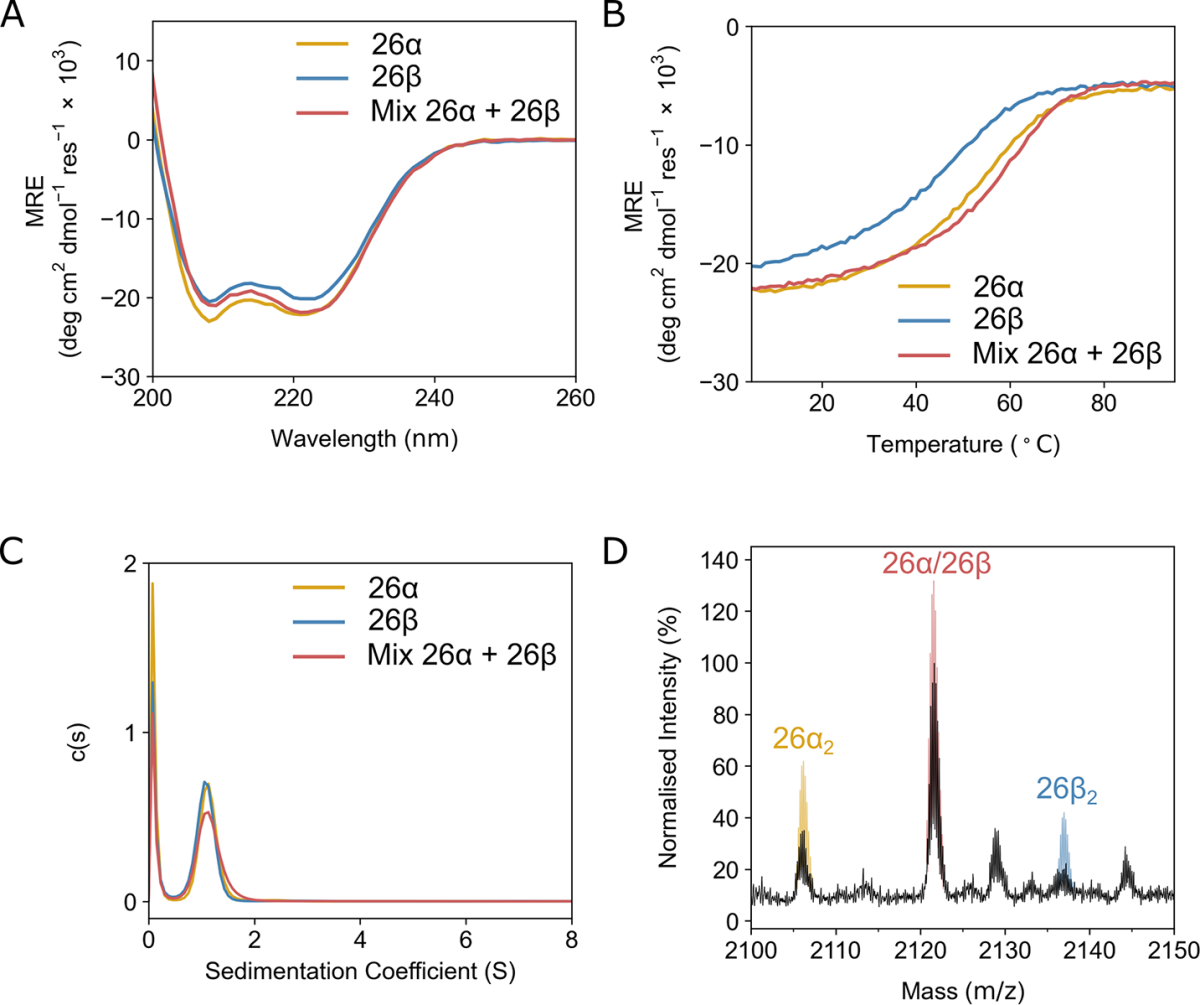
*In vitro* biophysical characterization of hairpin pair 26. (A) CD spectra at 5 °C of the peptides 26α (yellow) and 26β (blue) alone and when mixed (26α/26β, red). (B) Thermal unfolding following the CD signal at 222 nm. Same coloring as in panel A. Second derivatives of melting curves returned melting temperatures of 55 °C for 26α, 48 °C for 26β, and 60 °C for the mixture 26α/26β. (C) Sedimentation-velocity AUC data colored as in panel A. Fits returned molecular weights corresponding to dimers for each peptide. (D) Native mass spectrum (black line) for the 26α/26β mixture showing the region corresponding to the 5 H^+^ state of the dimers. Simulations of the 5 H^+^ protonated state for the homodimer 26α (yellow), the homodimer 26β (blue), and the heterodimer 26α/26β (red) are overlaid. The peaks at 2128.8 *m*/*z* and at 2144.2 *m*/*z* correspond to the TFA adduct (+ 113 Da) of the homodimer 26α and the heterodimer 26α/26β, respectively. Conditions for A–C: 50 μM peptide, PBS, 500 μM TCEP, pH 7.4. Conditions for D: 50 μM peptide, ammonium acetate buffer, 500 μM TCEP, pH 7.0.

By CD spectroscopy, the individual peptides showed various levels of α-helical folding at 50 μM concentration in phosphate buffered saline (PBS) (Figure 5A, Figures S14A-S17A). To test for heteroassociation, CD spectra for equimolar α + β mixtures were also measured and compared to the calculated average of the spectra signals of the individual. For the 9α/9β and 14α/14β combinations there were clear increases in helicity upon mixing compared to the theoretical average. However, for the 21α/21β and 26α/26β pairs, the spectra indicated a similar degree of folding between the individual peptides and the corresponding mixtures. Therefore, the stabilities of the folded states were assessed by thermal denaturation monitoring the CD signal at 222 nm (Figure 5B, Figures S14B–S17B). All of the individual peptides and mixtures showed thermal unfolding transitions with one hairpin in each pair (namely, 9β, 14β, 21α, and 26β) generally being less stable than its partner. Importantly, the cognate α + β mixtures all had more-sigmoidal unfolding curves with higher midpoint melting temperatures compared with the theoretical averages from the individual peptides. This indicates the predominance of heteromeric species in solution upon mixing.

We used sedimentation velocity (SV) experiments in AUC to probe the oligomeric states of the individual peptide and the cognate mixtures at 50 μM total peptide concentration in PBS. In all cases, we observed monodispersed species with molecular weights corresponding closely to dimers (Figure 5C, Figures S18–S21). However, these AUC experiments cannot distinguish between homo- and heterodimers. Therefore, we turned to native mass spectrometry to complete our solution-phase biophysical analysis of the chosen α/β pairs. For each combination, the individual peptides and the α + β equimolar mixture of were analyzed at 50 μM total concentration in ammonium acetate buffer, pH 7.0. All of the individual peptides showed some homodimer formation (see the ionization state of 5 H^+^ or 7 H^+^ for dimers, Figure S22–S25). However, for the mixtures, species from the heterodimers generally dominated the spectra, with those from the homodimers in the minority. This is illustrated for the 26α/26β pair in Figure 5D.

In summary to this section, the *in vitro* solution-phase biophysical data confirm that the cognate pairs selected from the in *vivo* BiFC assay, i.e., 9α/9β, 14α/14β, 21α/21β, and 26α/26β,all form stable, helical heterodimers consistent with the targeted 4HB structures. Nonetheless, these data also indicate that some of the individual peptides can form helical homodimers, albeit with lower thermal stabilities that the cognate mixtures. This seems to be at odds with the extended in-cell assays described above. This may reflect a difference between the high concentration used in these *in vitro* assays and the concentration at which our constructs were in cells: although some cellular proteins are present at concentrations >10 μM, many are expressed at concentrations lower than μM and often closer to nM.^49,50^ To test this hypothesis, we measured equilibrium and variable-temperature CD spectra at different concentrations of peptides from the pair 26 (Figure S26). At the lowest concentration of peptides that gave measurable CD spectra (i.e., 1 μM total peptide), second derivatives of melting curves returned melting temperatures of 43 °C for 26α, 30 °C for 26β, and 48 °C for the mixture 26α/26β. This suggests that the 26α/26β heterodimer should be well formed and stable, whereas the individual peptides should be unfolded or partially folded at concentrations close to those anticipated in cells and at the required temperature for cell experiments (37 °C).

### An X-ray Crystal Structure of the 26α/26β Complex Reveals a Syn 4-Helix Bundle

**Figure 6:**
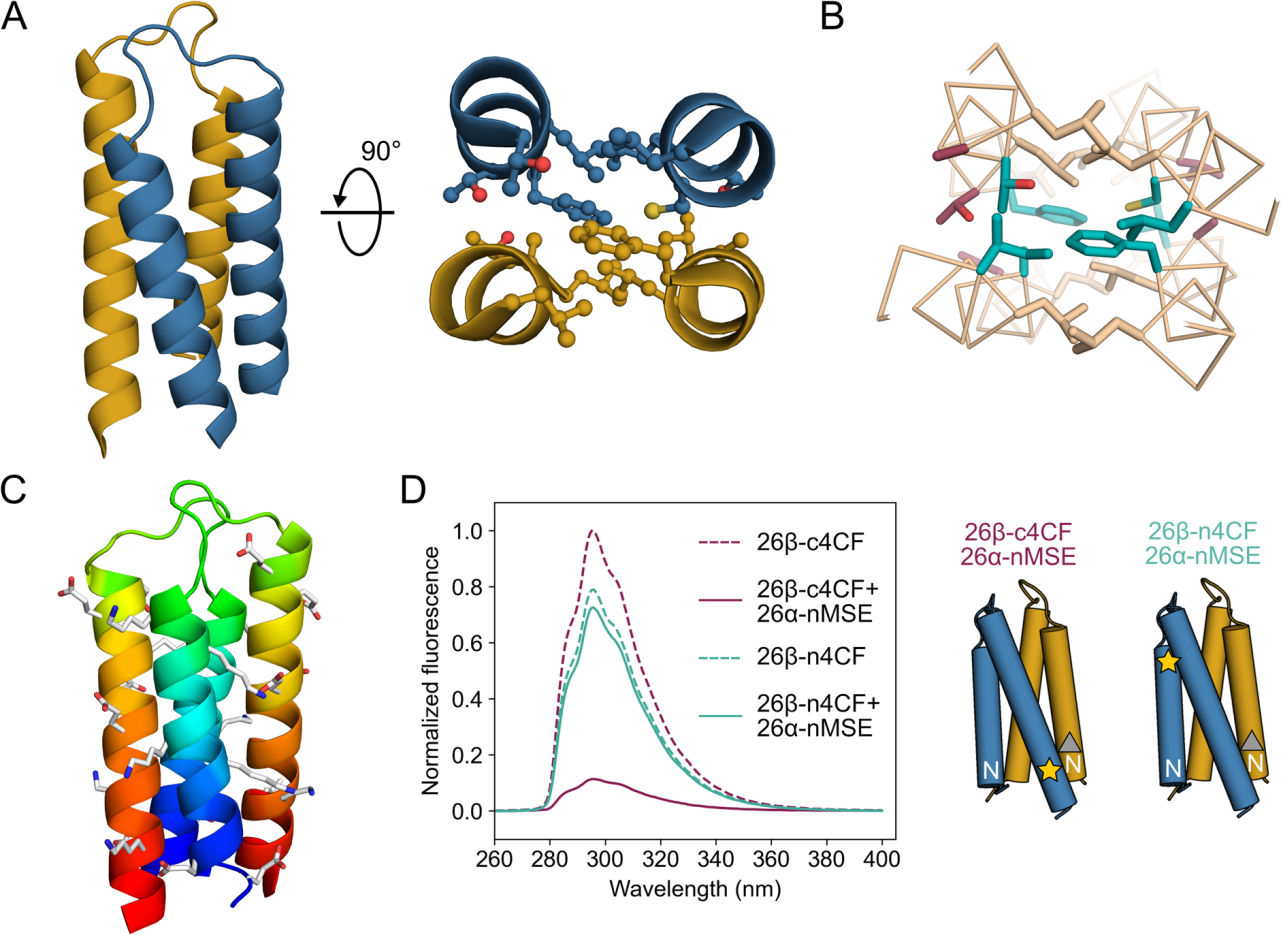
X-ray crystal structure of the heterodimer 26α/26β (PDB ID, 8bct, Table S1). (A) Left: Cartoon with hairpin 26α depicted in yellow and 26β in blue. Right: Cross section through one heptad repeat showing the knobs-into-holes packing. (B) View into the core with modifications at *a* positions in cyan and at *e* in red. (C) Chainbow representation with each chain colored from blue (*N* terminus) to red (*C* terminus) and with charged side chains shown as sticks. (D) Fluorescence-quenching assay for the 26α/26β pair. 4CF refers to 4-cyano-l-phenylalanine fluorophore (bottom panels, yellow star), and MSE to l-selenomethionine fluorescence quencher (bottom panels, gray triangle); and “n” and “c” indicate the fluorophore is near the *N* and *C* termini, respectively. Color code as in panel A. Conditions: 50 μM concentration of each peptide in phosphate buffer, pH 7.4.

We attempted to crystallize all four selected pairs and were successful with 26α/26β. This gave good X-ray diffraction data to 1.57 Å resolution, and a structure (PDB ID, 8bct) was solved by molecular replacement using the parent hairpins CC-HP1.0. The 26α/26β complex formed the targeted heterodimer of hairpins, which was confirmed as a 4-helix CC with knobs-into-holes packing using the program Socket2 (Figure 6).^51^ The heterodimer has a *syn* topology. As mentioned above, this is different to the parent CC-HP1.0 structure, which crystallized in an *anti* conformation. However, the *syn* conformation is consistent with the designed charge patterning of CC-HP1.0 as depicted in the helical wheels for the design (Figure 1B), and with AlphaFold2 predictions (Figure S4). Moreover, although the *anti* and *syn* forms are likely close in energy, and the linkers in the split-YFP assay should be flexible enough to allow both topologies, we suggest that the *syn* arrangement is favored as discussed below.

To test whether the *syn* topology of the 26α/26β complex was robustly maintained in solution, we took advantage of the heteromeric nature of the system to perform fluorescent-quenching experiments established by Raleigh et al.^52^ Based on an analysis of the crystal structures for the *syn* (i.e., 26α/26β) and the *anti* (CC-HP1.0) topologies, we incorporated the fluorescent 4-cyanophenylalanine (4CF) at the *C* terminus of the 26β peptide (26β-c4CF, Table S2 and Figure S13) and its quencher, selenomethionine (MSE), at the *N* terminus of the 26α hairpin (26α-nMSE, Table S2 and Figure S13); two positions that would be close in a *syn* topology. We synthesized another 26β variant carrying the 4CF residue at position 18 in the *N*-terminal helix (26β-n4CF, Table S2, Figure S13), which should be too far from the MSE residue to be quenched in a *syn* assembly with 26α-nMSE. Indeed, the latter combination showed fluorescence similar to the 26β-n4CF alone (Figure 6D). In contrast, near-complete quenching occurred when 26β-c4CF and 26α-nMSE were mixed, demonstrating the proximity of the 4CF and MSE residues. These data are consistent with 26α/26β forming a heterodimer with a *syn* topology in solution as observed in the experimental X-ray crystal structure.

### The Selected Dimers of Hairpins Are Orthogonal and Portable into Different Cellular Contexts

**Figure 7:**
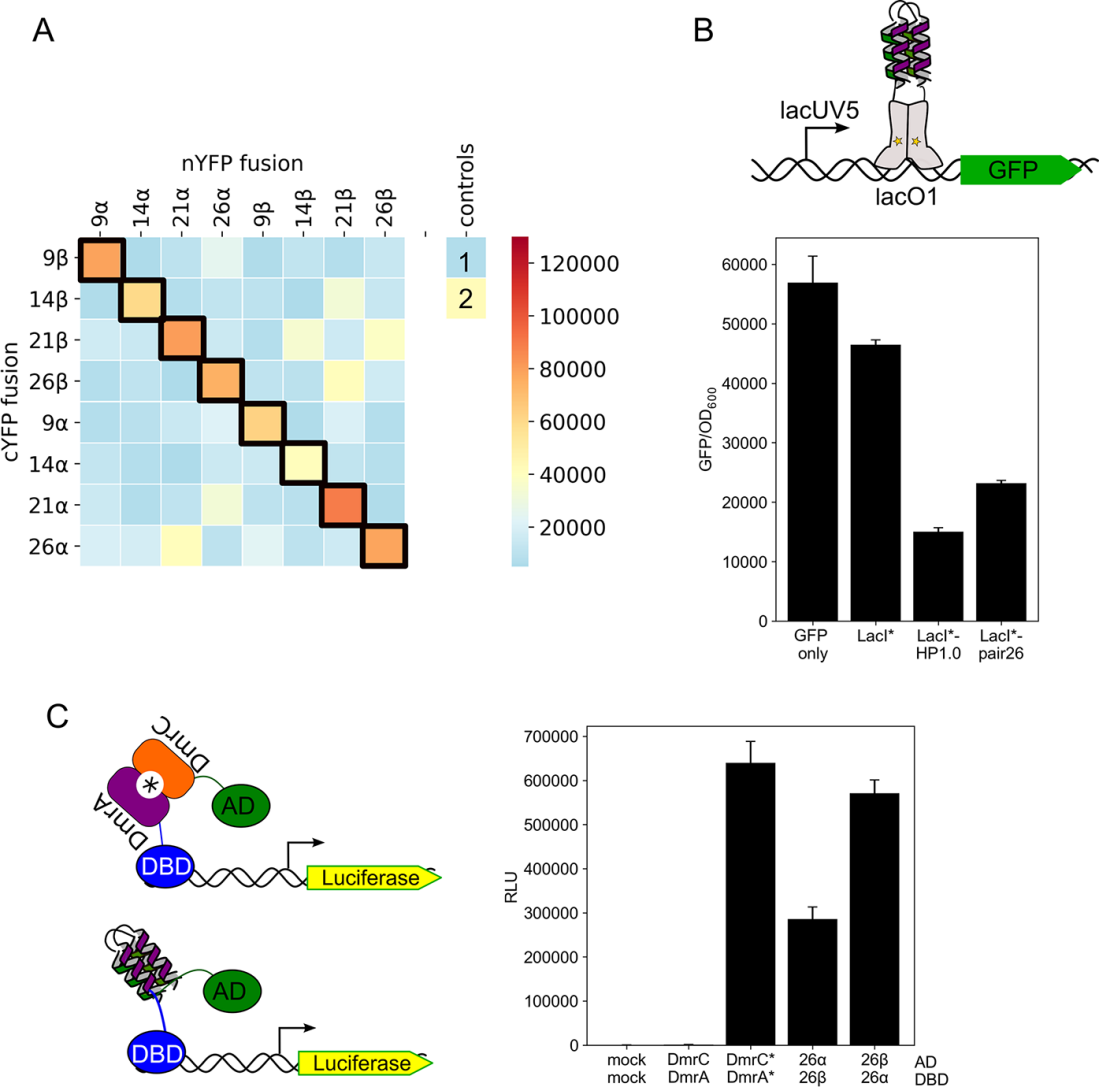
Heterodimeric 4HBs are orthogonal and portable into other subcellular contexts. (A) Heatmap showing YFP activity to examine the orthogonality of the different hairpin heterodimers. Each hairpin was fused to nYFP or cYFP and cells were transformed in the combinations indicated. Transformations were carried out in 96-well plates and YFP fluorescence was normalized to the OD_600_ of the bacterial cell culture. Each square is the average of at least two independent transformations. Controls: 1, no CCs; and 2, CC-HP1.0. (B) Cartoon and bar chart showing repression of a GFP gene expressed from the *lacUV5* promoter in *E. coli*. Hairpins were fused to LacI*, a dimerization mutant of the LacI repressor also lacking the tetramerization domain. Fusion with CC-HP1.0 or 26α/26β increased repression of GFP. GFP fluorescence was normalized to the OD_600_ of the bacterial cell culture, averaged from three different cultures, and shown with standard deviations. (C) Cartoons and bar chart showing the activation of transcription in human HEK293 cells elicited by the dimerization of 26α/26β. The DNA-binding (DBD) and activation domains (AD) of a split transcription factor are brought together by the PPI, which initiates transcription of a luciferase reporter gene. As a positive control the iDimerize DmrA and DmrC proteins dimerize in the presence of A/C heterodimerizer (*), and also activate transcription of luciferase. Luminescence is shown as relative light units (RLU) and was the average of 6 independent experiments with standard deviations shown.

For the generated heterodimeric 4HBs to function as components of a wider synthetic biology toolkit for different applications, ideally they should be usable together and across multiple cell types. To probe the orthogonality of the pairs in *E. coli*, we used the split-YFP to test the 4 chosen α/β pairs in an all-against-all manner (Figure 7A). As the heat map shows, we only observed appreciable YFP signals (orange coloring) for the cognate pairings, and there was little signal for the noncognate pairings. Moreover, all but one of the cognate pairs (14α/14β) outperformed the parent CC-HP1.0 control, correlating with 14α/14β having the lowest thermal stability measured by CD *in vitro*. We note that despite the higher *in vitro* stability of CC-HP1.0 compared to the other heterodimeric hairpins, the parent CC-HP1.0 constructs gives less fluorescence in the BiFC assay. This is most likely due to nonproductive self-complementation, i.e., homodimers, of the nYFP and cYFP fused to CC-HP1.0.

To demonstrate functionality of the heteromeric hairpins in different cellular applications, we cloned the 26α/26β pair into an alternative reporter system in *E. coli* (Figure 7B). Specifically, we demonstrated that this pair could direct oligomerization of a monomeric Lac repressor mutant (LacI*)^9^ enabling it to bind DNA and repress the expression of a reporter GFP gene (Figure 7B). Next, to demonstrate the application of the 26α/26β pair in different cell types, we introduced it into mammalian (HEK293) cells as separate fusion proteins to either the activation (AD) or DNA-binding domains (DBD) of a synthetic split transcription factor (iDimerize Regulated Transcription System, Takara Bio USA Inc.). Heterodimerization of the 26α/26β pair was able to bring together the fused transcription factor domains, forming an active complex capable of inducing expression of a luciferase reporter from an orthogonal promoter (Figure 7C). We tested the 26α/26β pair as fusions in both orientations and both induced luciferase expression. Indeed, the 26α–DBD and 26β–AD configuration induced similar levels of luciferase expression to the iDimerize positive control, a rapamycin analogue (A/C heterodimerizer)-inducible (DmrA/DmrC) interaction.

## Conclusion

Our aim was to generate straightforward and portable *de novo* protein–protein interaction (PPI) modules that operate in cells. We have selected PPI pairs from libraries expressed directly in *E. coli*, rather than designing PPIs *in silico*, characterizing them *in vitro*, and then transplanting them into cells. We target 4-helix bundles (4HBs), starting with the rational design of a homodimer of helical hairpins. Using this, we generate a library of core mutants, which is fused to each of the two halves of a split-YFP system.^41^ This allows direct screening for competent pairs of hairpins in cells using FACS. Encouragingly, the selected sequences are not random, but consistent with the formation of consolidated hydrophobic cores. Further in-cell assays identify four cognate and nonpromiscuous heteromeric pairs of hairpins. These have been characterized fully in solution and with a high-resolution X-ray crystal structure. The latter reveals a heterodimer of α-helical hairpins with the *syn* topology. Finally, we show that this pair can substitute for two other PPIs in *E. coli* and mammalian cells.

We recognize that other synthetic biology applications of these PPIs may require further testing and even modification to take into account different cellular contexts. For example, while our biophysical and structural studies are consistent with the targeted heterodimers of hairpins, these had to be conducted at high μM concentrations where both the hetero- and homomeric complexes are folded. We find no evidence for homomers in cells. However, other factors may be at play in the complex environment of the cell: we presume that the concentration of our constructs within cells combined with the availability of transient nonspecific interactions with other cellular components reduces the propensity for assembly of the noncognate and homomeric pairings relative to the selected heterodimeric interactions.

Placing our work in the context of *de novo* protein design: Many others have targeted 4HBs,^1,3,20,32^ although the majority focus on minimal, rational, or computational design and *in vitro* characterization. Hecht’s work on selecting stable proteins in cells from *hp*-patterned sequence libraries (where *h* and *p* are hydrophobic and polar amino acids, respectively) is an exception.^53^ Moreover, like Hecht, we show that screening directly in cells allows a more-direct approach to downstream subcellular applications of the selected *de novo* proteins. That said, we and others are using rationally and computational designed peptides and proteins in cells for synthetic biology applications with increasing success.^9,11,16,30,35^

More specifically, others have engineered natural dimerizing hairpins, notably using ROP, and designed *de novo* hairpins to produce 4HBs.^20,31,54−56^ These studies raise questions on how overall bundle architecture is specified and controlled. For example, variants of ROP access three possible states: the wild type has an *anti* arrangement of hairpins (PDB ID, 1rop);^57^ a designed core mutant accesses the flipped *syn* state (1f4m);^58^ and the A31P point mutation, which is in the loop, forms a bisecting U (1b6q).^39^ The latter is also observed in a *de novo* designed hairpin from Hill and DeGrado (1qp6).^40^ This potential for structural plasticity challenges protein designers. For our initial design and starting point, we targeted the *syn* arrangement. An AlphaFold2-multimer prediction^59−61^ for the starting sequence is *syn* (Figure S4). However, the experimental X-ray crystal structure of the parent 4HB, CC-HP1.0, has the *anti* arrangement. Because of this uncertainty, for the in-cell selections, we made the linkers of the hairpin-split-YFP fusions long enough to access all architectures. Interestingly, the solution-phase and crystal structure for one of the selected heterodimeric pairs is *syn*. Finally on this point, we searched the CC+ database for dimers of hairpins forming CC 4HBs.^62^ We find that *syn* arrangements are slightly more represented than *anti* (Table S3).

Finally on context, core-directed protein design and engineering has a long history.^31,56^ This has included repacking the cores of natural proteins;^63−67^ methods development for design;^68,69^ and designing completely *de novo* frameworks.^20,70,71^ The resulting principles include the conservation of core volume and hydrophobicity, and the importance of side-chain complementarity. Our work adds to this by targeting protein–protein interfaces. While others have explored hydrogen-bonding networks to design specific PPIs as heterodimers of hairpins using computational methods,^10,20^ we screen a range of side-chain volumes and hydrophobicities directly in cells. Interestingly, we find a shift from more-random sequences of the starting library toward those with predicted core volumes and hydrophobicities similar to the parent hairpin. Moreover, we find a preference for large hydrophobic residues (Leu and Phe) at sites anticipated to contribute most to the hydrophobic core, and a preference for smaller Ala residues at flanking sites where the interhelical distances are expected to be shorter.^28^ While it is difficult to make inferences from small libraries that cannot and do not sample all of the possible sequence space, it is interesting that we select sequences that show rationalizable trends. This is analogous to folded variants of ubiquitin selected from an exhaustive library of hydrophobic-core mutations, which gives sequences close to the wild type.^66^

In addition to these reflections and insights, our study has delivered new cognate and orthogonal PPI pairings that we hope will prove useful to others in synthetic biology. Our future studies will explore how the system can be used to produce more-complex PPIs including those that respond to post-translational modifications and small-molecule, peptide, and protein binding. Ultimately, our aim is to produce regulatable PPIs that would have many applications across synthetic biology.^8,72,73^

## Methods

### Plasmids

The construction of the plasmids used in this work is described in more detail in the Supporting Information. In short, the plasmids pBAD-nYFP-xa and pVRc-cYFP-xa were made by inserting EYFP aa1–154 and EYFP aa 155–238^41^ into the pBAD and pVRc vector backbones, which are described in Smith et al.^9^ A DNA fragment encoding CC-HP1.0 was inserted *C*-terminally to the YFP fragments to create pBAD-nYFP-HP1.0 and pVRc-cYFP-HP1.0. In order to assay transcriptional repression of GFP, DNA encoding CC-HP1.0 and CC-HP1.26α were cloned into pBADLacI*^9^ and DNA encoding CC-HP1.26β was cloned into pVRcLacI*^9^*C*-terminally of LacI*. For mammalian cell dimerization assays the hairpins 26α/26β were cloned into the pHet-Act1–2 vector (Takara Bio USA Inc.) as fusions to the activation domain and the DNA binding domain respectively (26α-AD/DBD-26β) and in the opposite configuration (26β-AD/DBD-26α).

### Constructing the Hairpin Library

A library (CC-HP1.0-lib) consisting of CC-HP1.0 with 8 variable amino acids, Leu at the *a* positions 10, 17, 36, and 43 and Ala at the *e* positions 7, 14, 33, and 40, was designed and codon optimized for *E. coli*. Degenerate codons were used to introduce variability at these 8 positions; namely, NBC at the *a* position, and RST at the *e* position (IUPAC nucleotide code). At the *a* positions NBC encodes combinations of Ala, Cys, Phe, Gly, Ile, Leu, Pro, Arg, Ser, Thr, and Val; and at the *e* positions RST encodes Gly, Ala, Ser, and Thr. The library was synthesized as a linear combinatorial library (Eurofins genomics) and was cloned into pBAD-nYFP-xa and pVRc-cYFP-xa *C*-terminally of the YFP fragments. One Shot OmniMAX 2 T1^R^ Chemically Competent *E. coli* (Invitrogen) were transformed with this library as described in the Supporting Information and contained 1.9 × 10^5^ transformants. In order to sequence noninteracting (“dark”) hairpins, TB28 (MG1655ΔLacIZYA)^74^*E. coli* cells were also transformed with the splitYFP-CC-HP1.0-lib fusions as described in the Supporting Information. This library contained approximately 1.35 × 10^7^ transformants.

### Fluorescence Activated Cell Sorting (FACS)

To culture cells for FACS 100 μL of a glycerol stock of CC-HP1.0-lib was diluted into 50 mL LB media + 25 μg/mL chloramphenicol + 100 μg/mL ampicillin and grown at 37 °C for 6 h. 50 μL of this culture was then used to inoculate M9 minimal media + 0.25% glycerol + 0.5 mM CaCl_2_ + 2 mM MgSO_4_ + 2 μg/mL thiamine + 0.2% casamino acids + 25 μg/mL chloramphenicol + 100 μg/mL ampicillin + 0.2% arabinose. Cultures were grown at 30 °C for 18 h. 0.5 mL cells were harvested by centrifugation and resuspended in 0.5 mL PBS (137 mM NaCl, 2.7 mM KCl, 10 mM Na_2_HPO_4_, 2 mM KH_2_PO_4_). Samples were then diluted in PBS before analyzing on the BD Biosciences FACS Aria II SORP (Special Order Research Project) (Franklin Lakes, NJ, USA). The Aria II is equipped with 4 lasers (Violet 405 nm, Blue 488 nm, Yellow 561 nm and Red 640 nm). We used 70 μm nozzle tip, 70 PSI. For more sensitive detection of light scatter we used the 405 nm laser for threshold (with 405/20 nm Bandpass filter). YFP was detected using the blue laser via 530/30 nm BP filter. Gates were created as described in the Supporting Information and individual cells were sorted into wells of a 96-well plate containing 200 μL LB + 25 μg/mL chloramphenicol + 100 μg/mL ampicillin. The plates were then incubated at 37 °C with shaking (1000 rpm) in a Stuart SI505 microtiter plate incubator for 18 h.

The sequences of the hairpins from the cells isolated by FACS were determined by amplifying DNA encoding the hairpins by PCR as described in the Supporting Information. DNA fragments of the expected length were sequenced by Eurofins genomics. Plasmid DNA encoding pBAD-nYFP-α fusions and pVRc-cYFP-β fusions was subsequently purified from the mixed cultures as described in the Supporting Information.

### YFP Assays

To assay reconstitution of split YFP, TB28 cells were transformed with plasmids expressing nYFP and cYFP fusion proteins as indicated in the text. Overnight cultures from at least 3 separate colonies were grown at 37 °C in M9 minimal media + 0.25% glycerol + 0.5 mM CaCl_2_ + 2 mM MgSO_4_ + 2 μg/mL thiamine + 0.2% casamino acids (+ 25 μg/mL chloramphenicol + 100 μg/mL ampicillin where required). The overnight cultures were used to inoculate 10 mL of the same medium containing 0.2% arabinose in induce expression of the split YFP fusion proteins. Cultures were grown at 30 °C until they reached an OD_600_ ∼ 0.5. Five milliliters of culture was centrifuged for 10 min at 4500*g* and the pellet was resuspended in 250 μL PBS (137 mM NaCl, 2.7 mM KCl, 10 mM Na_2_HPO_4_, 2 mM KH_2_PO_4_). 2 × 100 μL cell suspension was placed in a black, flat bottomed 96 well plate and the fluorescence read in a Clariostar plus microplate reader (BMG Labtech) on YFP settings (ex 497–515 nm, em 540–620 nm). YFP fluorescence was normalized by dividing by the OD_600_ of the bacterial culture.

When screening for hairpin promiscuity and when testing the orthogonality of the candidate heterodimeric hairpins, pairwise transformations of split-YFP fusion proteins were carried out in 96-well plates. pBAD-nYFP and pVRc-cYFP plasmids expressing hairpins selected from the library screening was added to 50 μL CaCl_2_ competent TB28 cells. After heatshock for 90 s at 42 °C the cells were added to 150 μL LB and incubated at 37 °C for 1 h with shaking at 250 rpm in an orbital shaker. Ten μL cells were then added to 200 μL LB containing 25 μg/mL chloramphenicol and 100 μg/mL ampicillin in a 96 well plate. Cells were grown at 37 °C with shaking (1000 rpm) in a Stuart SI505 microtiter plate shaking incubator for 18 h. To assay for YFP fluorescence 10 μL from each culture of transformed cells was added to 200 μL M9 minimal media containing 0.2% arabinose and the additives detailed above. Cells were grown at 30 °C for 6 h and YFP activity was assayed in a Clariostar plus microplate reader as described above. YFP fluorescence was normalized by dividing by the OD_600_ of the bacterial culture.

### GFP Repression Assays

GFP repression assays were carried out as described by Smith et al.,^9^ except that fluorescence was measured in the Clariostar plus microplate reader on GFP settings (ex 470–515 nm, em 515–520 nm).

### Mammalian Cell Culture, Transfection, and Luciferase Assay

HEK293 cells (ATCC, CRL-1573) were cultured in DMEM supplemented with 10% FBS grown at 37 °C in a humidified 5% CO_2_ incubator. For transfection, HEK293 cells were plated in 96-well solid-white flat-bottom TC-treated plates (Corning) at 2.5 × 10^4^ cells per well. The following day, cells were transfected with pHet-Act1–2 vector (or variants thereof; 66 ng per well) and pZHD1-Luc reporter vector (Takara Bio; 29 ng per well) using lipofectamine 2000 (Invitrogen), according to the manufacturer’s instructions. At 24 h post-transfection, media was replaced either with fresh media alone or with media containing 1000 nM rapalog inducer (A/C heterodimerizer, Takara Bio; for positive control only). At 6 h postinduction, the plate was equilibrated to room temperature for 10 min before 100 μL of Steady-Glo reagent (Promega) was added to each well. The plate was incubated for 10 min at room temperature before luminescence was measured used an Envision plate reader (PerkinElmer).

### Statistical Analysis of Hairpin Libraries

A custom Python script was written to calculate the possible combinations of amino acid sequences in CC-HP1.0-lib. Each pair of the heterodimeric hairpins contains 16 variable amino acids (8 × Leu@a and 8 × Ala@e in 2 copies of CC-HP1.0). The theoretical side-chain volumes in the protein interior (Å^3^)^46^ and hydrophobicities (using the hydropathy scale of Δ*G*_tr_ corrected for self-solvation)^47^ were calculated at these 16 positions. These data were then used to plot normal distributions of the theoretical side-chain volumes and hydrophobicities of all the possible combinations of hairpin pairs in CC-HP1.0-lib. The side-chain volumes and hydrophobicities of the 16 variable amino acids of the candidates selected from the libraries were calculated and overlaid on the normal distribution plots.

To compare the observed and expected proportions of each amino acid at each variable site in the candidates selected from CC-HP1.0-lib, the statistical method of standard error of proportion was used. Here our heatmap shows z-scores, calculated as described by Woolfson and Alber,^48^ where a value of zero indicates that the amino acid is found at the same proportion as expected by chance.

### *In Vitro* Characterizations of Peptides

Detailed methods for the *in vitro* synthesis and purification of peptides and their solid-phase biophysical characterization are provided in the Supporting Information.

### Fluorescence Quenching Experiments

Fluorescent quenching experiments were performed following previously published procedure.^52^ Peptide samples were prepared to have equimolar ratio of 26α and 26β analogues at 50 μM of the CN-Phe-containing peptide in phosphate buffer (8.2 mM sodium phosphate dibasic, 1.8 mM potassium phosphate monobasic), 500 μM TCEP, pH 7.4. Fluorescence emission spectra from 260 to 400 nm were recorded on a Jasco Fluorimeter, on a 10 × 10 mm quartz cuvette with an excitation wavelength of 240 nm. Every fluorescence emission spectrum was obtained by averaging of 3 scans and subtracting the background signal of buffer and cuvette.

### X-ray Crystal Structure Determination

Details of the X-ray crystal structure determination are provided in the Supporting Information.

## Supporting Information Available

Additional and detailed methods: construction of plasmids and hairpin libraries, FACS sorting, PCR amplification from library candidates, isolation of plasmids, solid-phase peptide synthesis, peptide purification and purity analysis, CD spectroscopy, analytical ultracentrifugation, native MS, X-ray structure determination, AlphaFold modeling, Bude alanine scanning, sequences of constructs used in this work; Supplementary figures: representation of the possible topologies for a dimer of hairpins, additional views of the crystal structure of CC-HP1.0, AlphaFold2-multimer models, Bude alanine scanning results, additional FACS plots, additional statistical analysis, additional split-YFP assay results, HPLC and MS traces for all peptides, additional biophysical characterizations (CD, analytical ultracentrifugation, native MS) for all peptides; Supplementary tables: merging and refinement statistics for all X-ray crystal structures, sequences of the synthesized peptides, comparison of *syn* and *anti* conformations found within characterized structures of 4-helix coiled coils and obtained from CC+ database; Supplementary references (PDF)
