# Supplementary material for: Design and Selection of Heterodimerizing Helical Hairpins for Synthetic Biology
Source: ACS Synth Biol. 2023 May 24;12(6):1845–58. doi: 10.1021/acssynbio.3c00231 (PMC10278171; doi:10.1021/acssynbio.3c00231)
Supplement: Supplementary file 1 — sb3c00231_si_001.pdf [file sb3c00231_si_001.pdf]

Design and selection of heterodimerizing helical hairpins for synthetic biology

Abigail J. Smith,[1†] Elise A. Naudin,[2†] Caitlin L. Edgell,[1,2] Emily G. Baker,[1,2] Bram Mylemans,[2] Laura FitzPatrick,[3] Andrew Herman,[4] Helen M. Rice,[4] David M. Andrews,[5] Natalie Tigue,[3] Derek N. Woolfson[1,2,6,*] and Nigel J. Savery[1,6*]

[1]School of Biochemistry, University of Bristol, Biomedical Sciences Building, University Walk, Bristol BS8 1TD, UK

[2]School of Chemistry, University of Bristol, Cantock's Close, Bristol BS8 1TS, UK

[3]BioPharmaceuticals R&D, AstraZeneca, Cambridge Science Park, Darwin Building, Cambridge CB4 0WG, UK

[4]Flow Cytometry Facility, School of Cellular and Molecular Medicine, University of Bristol, Biomedical Sciences Building, University Walk, Bristol BS8 1TD, UK

[5]Oncology R&D, AstraZeneca, Granta Park, Cambridge CB21 6GH, UK

[6]BrisEngBio, School of Chemistry, University of Bristol, Cantock's Close, Bristol BS8 1TS, UK

[†]Both authors contributed equally to this work

*To whom correspondence should be addressed:

D.N.Woolfson@bristol.ac.uk and N.J.Savery@bristol.ac.uk

TABLE OF CONTENTS

ADDITIONAL METHODS **S3**

1.1 Construction of plasmids and DNA codon libraries **S3**

1.1.1 Plasmids S3

1.1.2 Construction of a hairpin library S3

1.2 Library Screening **S4**

1.2.1 FACS sorting S4

1.2.2 PCR amplification of hairpins from the library candidates S5

1.2.3 Isolation of plasmid DNA encoding YFP-HP fusions S6

1.3 *In vitro* characterizations of peptides **S6**

1.3.1 General S6

1.3.2 Peptide synthesis and purification S6

1.3.2.1 Automated microwave Fmoc/tBu solid-phase peptide synthesis (SPPS) S6

1.3.2.2 Semi-preparative High Performance Liquid Chromatography (HPLC) S7

1.3.2.3 Analytical HPLC S7

1.3.2.4 Mass spectrometry S8

1.3.2.5 Peptide concentration determination S8

1.3.3 Solution-phase biophysical characterizations S8

1.3.3.1 Circular dichroism (CD) spectroscopy S8

1.3.3.2 Analytical ultracentrifugation S9

1.3.3.3 Native mass spectrometry (MS) S10

1.4 X-ray crystal structure determination **S10**

1.5 AlphaFold **S11**

1.6 Bude alanine scanning **S11**

1.7 Sequences of Constructs used in this work **S11**

SUPPLEMENTARY DATA **S13**

2.1 Supplementary Figures **S13**

2.2 Supplementary Tables **S39**

SUPPLEMENTARY REFERENCES **S42**

ADDITIONAL METHODS

1.1 Construction of plasmids and DNA codon libraries

1.1.1 Plasmids

The plasmids pBAD-nYFP-xa and pVRc-cYFP-xa were made by inserting EYFP aa1-154 and EYFP aa 155-238[1] into the pBAD and pVRc vector backbones, which are described in Smith *et al.*[2] DNA encoding split YFP fragments was inserted into these backbones at NheI/XbaI sites. A DNA fragment encoding CC-HP1.0, codon optimized for *E. coli*, was synthesized by Eurofins genomics and inserted into pBAD-nYFP-xa and pVRc-cYFP-xa in frame with the YFP fragments at XbaI/Acc65I restriction sites to create pBAD-nYFP-HP1.0 and pVRc-cYFP-HP1.0. In order to assay transcriptional repression of GFP, DNA encoding CC-HP1.0 and CC-HP1.26α were cloned into pBADLacI*[2] at XbaI/Acc65I sites. DNA encoding CC-HP1.26β was cloned into pVRcLacI*[2] at XbaI/Acc65I sites.

For mammalian cell dimerization assays the hairpins 26α/26β were cloned into the pHet-Act1-2 vector (Takara Bio) as fusions to the activation domain and the DNA binding domain respectively (26α-AD/DBD-26β) and in the opposite configuration (26β-AD/DBD-26α). The DNA sequences were codon optimized for expression in human cells and synthesized as DNA strings by GeneArt (ThermoFisher) which contained 30bp overlaps with the corresponding 5′ and 3′ vector fragments. The NEBuilder HIFI DNA assembly kit (NEB) was used to assemble the digested vector fragments and DNA strings in a 4-fragment assembly reaction, according to manufacturer's instructions.

1.1.2 Construction of a hairpin library

A library (CC-HP1.0-lib) consisting of CC-HP1.0 with 8 variable amino acids, Leu at the ***a*** positions 10, 17, 36, and 43 and Ala at the ***e*** positions 7, 14, 33, and 40, was designed and codon optimized for *E. coli*. Degenerate codons were used which introduced variability at these 8 positions, namely NBC at the ***a*** position and RST at the ***e*** position (IUPAC nucleotide code). At the ***a*** positions NBC encodes either Ala, Cys, Phe, Gly, Ile, Leu, Pro, Arg, Ser, Thr or Val and at the ***e*** positions RST encoded Gly, Ala, Ser or Thr.

The library was synthesized as a linear combinatorial library (Eurofins genomics) and was cloned into pBAD-nYFP-xa and pVRc-cYFP-xa at XbaI/Acc65I sites. Ligations were transformed into One Shot™ OmniMAX™ 2 T1^R Chemically Competent *E. coli* (Invitrogen)

and plated onto multiple LB plates. Transformed colonies were then scraped from the plates in LB media and the cells were pelleted by centrifugation and stored at -80 °C. The number of transformants obtained for pBAD-nYFP-HPlib was $2.9x10^4$ and for pVRc-cYFP-HPlib was $1.2x10^4$. DNA was extracted from the cell pellets using a midiprep DNA purification kit (QIAGEN). Prior to DNA extractions, the OmniMAX cells containing pBAD-nYFP-HPlib were used to seed a 50 mL culture which was made chemically competent using $CaCl_2$ following standard procedures. These competent cells were transformed with 10 ng pVRc-cYFP-HPlib plasmid DNA in 10 separate transformation reactions, cells were scraped from the LB agar plates and the library was stored at -80 °C as glycerol stocks. This library contained a $1.9x10^5$ transformants.

In order to sequence non-interacting ("dark") hairpins electrocompetent TB28 cells (MG1655ΔLacIZYA)[3] were transformed with 10 ng pBAD-nYFP-HPlib plasmid DNA in 10 separate transformation reactions. Transformed colonies were then scraped from the plates in LB media and used to seed a 50 mL culture in order to make electrocompetent TB28 + pBAD-nYFP-HPlib cells. These cells were then transformed with 10 ng pVRc-cYFP-HPlib plasmid DNA in 10 separate transformation reactions, cells were scraped from the LB agar plates and the library was stored at -80 °C as glycerol stocks. This library contained $1.35x10^7$ transformants. Dark cells were then isolated from this library by selecting non-fluorescent cells using FACS sorting as described below.

1.2 Library Screening

1.2.1 FACS sorting

To culture cells for fluorescence activated cell sorting (FACS) 100 μL of a glycerol stock of CC-HP1.0-lib was diluted into 50 mL LB media + 25 μg/mL chloramphenicol + 100 μg/mL ampicillin and grown at 37°C for 6 hours. 50 μL of this culture was then used to inoculate M9 minimal media + 0.25% glycerol + 0.5 mM $CaCl_2$ + 2 mM $MgSO_4$+ 2 μg/mL thiamine + 0.2% casamino acids + 25 μg/mL chloramphenicol + 100 μg/mL ampicillin + 0.2% arabinose. Cultures were grown at 30 °C for 18 hours. 0.5 mL cells were harvested by centrifugation and resuspended in 0.5 mL PBS (137 mM NaCl, 2.7 mM KCl, 10 mM Na_2HPO_4, 2 mM KH_2PO_4). Samples were then diluted appropriately in PBS before analysing on the BD Biosciences FACS Aria II SORP (Special Order Research Project) (Franklin Lakes, NJ, USA). The Aria II is equipped with 4 lasers (Violet 405 nm, Blue 488 nm, Yellow 561 nm and Red 640 nm). We used 70 μm nozzle tip, 70 PSI. For more sensitive detection of light scatter we used the 405

nm laser for threshold (with 405/20 nm Bandpass filter). YFP was detected using the blue laser via 530/30 nm BP filter.

Prior to cell sorting controls of OmniMAX cells expressing pBAD-nYFP-xa + pVRc-cYFP-xa (negative control) and pBAD-nYFP-HP1.0 + pVRc-cYFP-HP1.0 (positive control) were used to determine gates for non-fluorescent ("dark") cells and YFP positive cells (YFP+). Cells were gated on scatter (plotting SSC-H using the violet laser vs SSC using the blue laser) followed by gating on singlets (plotting SSC-A vs SSC-H using the violet laser), before plotting YFP signal (blue laser) vs SSC-H (violet laser). In order to select heterodimer candidates individual cells were collected from OmniMAX cells expressing the library CC-HP1.0-lib. Two gates, YFP1 and YFP2, were created in order to select "bright" cells which had greater fluorescence intensity than cells expressing splitYFP-HP1.0 fusions. Individual cells were sorted into wells of a 96 well plate containing 200 μL LB + 25 μg/mL chloramphenicol + 100 μg/mL. The plates were then incubated at 37 °C with shaking (1000 rpm) in a Stuart SI505 microtitre plate incubator for 18 hours. In order to produce a "dark" library of non-fluorescing cells a gate ("dark") was used to collect individual cells from TB28 cells expressing the library CC-HP1.0-lib as described above.

1.2.2 PCR amplification of hairpins from the library candidates

In order to determine the sequence of the hairpins from the clonal cultures isolated by FACS DNA encoding the hairpins was amplified by PCR using Phusion DNA polymerase (NEB). DNA encoding the hairpins fused to nYFP were amplified using the primers nYFP-F (5'-ggcattgacttcaaagagg-3') and pBADLCR (5'-agaccgcttctgcgttc-3'), and DNA encoding the hairpins fused to cYFP were amplified using the primers using the primers cYFP-F (5'-gtattaccctgggtatggacg-3') and pBADLCR. PCR reactions were set up in 96 well plates using Phusion polymerase (NEB) with 1 μl of a 1/10 dilution of the clonal cell cultures added to each PCR reaction. PCR reactions were performed under standard conditions. PCR products examined by electrophoresis on 7.5% polyacylamide/TAE gels and DNA fragments of the expected length were sequenced by Eurofins genomics.

1.2.3 Isolation of plasmid DNA encoding YFP-HP fusions

In order to test candidate heterodimers for their ability to homodimerize, both plasmids encoding both the nYFP and cYFP fusions were purified from the clonal cell cultures isolated by FACS. In order to separate the two plasmids pBAD-nYFP-α fusions were removed from the mixture by digestion with PciI/PvuI and pVRc-cYFP-β fusions were digested with ClaI/PvuII. These restriction digests were transformed into competent XLIBlue cells (Novagen) so that individual plasmids could be purified from the mixture. After selecting the least promiscuous hairpins the α hairpins were cloned into pVRc-cYFP-xa at XbaI/Acc65I sites and the β hairpins were cloned into pBAD-nYFP-xa at XbaI/Acc65I sites.

1.3 *In vitro* characterizations of peptides

1.3.1 General

All solvents, chemicals, and reagents were purchased from commercial sources and used without further purification. Fluorenylmethoxycarbonyl(Fmoc)-α-L-amino acids, Rink amide MBHA resin for solid-phase peptide synthesis (SPPS) and N,N-dimethylformamide (DMF) were purchased from Sigma-Aldrich, Fisher Scientific and Cambridge Reagents. Coupling reagents Oxyma Pure and diisopropylcarbodiimide (DIC) were purchased from Fluorochem and Sigma-Aldrich, respectively. Pyridine was purchased from Fisher Scientific; triisopropylsilane (TIPS) was from Acros Organics. Morpholine and trifluoroacetic acid (TFA) as well as all other chemicals (reagent grade) were purchased from Sigma-Aldrich.

1.3.2 Peptide synthesis and purification

1.3.2.1 Automated microwave Fmoc/tBu solid-phase peptide synthesis (SPPS)

Automated microwave SPPS was performed on a Liberty Blue (CEM) synthesizer with in-line UV monitoring. The synthesis was performed on a 0.1 mmol scale on Rink amide MBHA (0.65 mmol/g loading, 100-200 mesh) resins. Side-chain protections of the amino acids were as follows: Gln(Trt), Glu(O*t*Bu), Lys(Boc), Tyr(*t*Bu), Trp(Boc). The coupling of the amino acids was performed in DMF by adding 5 equivalents of amino acids (2.5 mL, 0.2 M), 10 equivalents of DIC in DMF (1.0 mL, 1 M) and 5 equivalents of Oxyma Pure in DMF (1 mL, 0.5 M). Standard couplings were performed at 90 °C for 4 min (100 W for 20 s, 60 W for 10 s, 35 W for 240 s). Standard deprotections were performed using 20% (v/v) morpholine in DMF at 90 °C for 1 min (125 W 30 s, 32 W 60 s). All peptides were manually capped with *N*-terminal

acetyl groups through addition of pyridine (0.5 mL) and acetic anhydride (0.25 mL) in DMF (9.25 mL), with shaking at room temperature (rt) for 20 minutes. The resin was washed 3 times with DMF followed by 6 times with DCM before cleavage. Peptides were cleaved from the resin and deprotected with addition of 10 mL of a mixture 95:2.5:2.5 v/v/v TFA/H_2O/TIPS, with shaking at rt for 2 hours. Cys-containing peptides were cleaved from the resin with addition of 10 mL of a mixture 92.5:2.5:2.5:2.5 v/v/v/v TFA/H_2O/TIPS/3,6-Dioxa-1,8-octane-dithiol (DODT), with shaking at rt for 2 hours. The SeMet-containing peptide was cleaved from the resin with addition of 10 mL of a mixture 88.7:5:3.3:3 v/v/v/v TFA/thioanisole/anisole/1,2-ethanedithiol (EDT), with shaking at rt for 2 hours. The TFA solution was filtered to remove the resin beads and was reduced in volume to ≤5 mL using a flow of N_2. Cleaved peptide was precipitated with cold diethyl ether (≈45 mL), isolated via centrifugation and dissolved in a 1:1 mixture MeCN/H_2O. Crude peptides were lyophilized to yield white powders.

1.3.2.2 Semi-preparative High Performance Liquid Chromatography (HPLC)

All peptides were purified by reverse phase HPLC (JASCO) using a Luna C18 (Phenomenex) column (150 × 10 mm, 5 μM particle size, 100 Å pore size). Crude peptide was dissolved at 7 mg/mL in 20% v/v MeCN in H_2O with 0.1% TFA, injected to the column and eluted with a 3 mL/min linear gradient (20-60%) of MeCN in H_2O with 0.1% TFA each over 30 minutes. Elution of the peptide was detected with in-line UV monitoring at 220 nm and 280 nm simultaneously. When required, a column oven (50 °C) was used to improve separation. Pure fractions were identified by analytical HPLC and matrix-assisted laser desorption/ionization–time of flight (MALDI-TOF) mass spectrometry, then pooled, and freeze-dried.

1.3.2.3 Analytical HPLC

Analytical HPLC traces were obtained using a Jasco 2000 series HPLC system using a Phenomenex Kinetex C18 (100 × 4.6 mm, 5 μm particle size, 100 Å pore size) column. Chromatograms were monitored at 220 and 280 nm wavelengths. The linear gradient was 20–80% MeCN in water (each containing 0.1% TFA) over 25 min at a flow rate of 1 mL/min.

1.3.2.4 Mass spectrometry

Matrix-assisted laser desorption/ionization–time of flight (MALDI-TOF) mass spectra were collected on a Bruker UltraFlex MALDI-TOF mass spectrometer operating in positive-ion reflector mode. Peptides were spotted on a ground steel target plate using a-cyano-4-hydroxycinnamic acid dissolved in 1:1 MeCN/H2O as the matrix. For final spectra of combined pure fractions, full electrospray ionization (ESI) MS spectra were acquired on a Synapt G2S (Waters) mass spectrometer equipped with an IMS-Q-TOF analyser and using an Advion Nanomate for robot chip-based nanospray ionization in positive mode. 5 µL of a 50 µM peptide solution in 1:1 MeCN/H2O were generally injected for the analysis. Deconvolution of electrospray data was performed with MagTran 1.03 (Amgen Inc.).

1.3.2.5 Peptide concentration determination

Peptide and protein concentrations were determined from absorbance at 280 nm measured in a Nanodrop 2000 (Thermo Scientific) spectrometer (ε_{280}(Trp) = 5690 cm^{-1}), or at 214 nm using a Cary-100 (Agilent) UV-Visible spectrometer by measuring the peptide bond.[4]

1.3.3 Solution-phase biophysical characterizations

1.3.3.1 Circular dichroism (CD) spectroscopy

Circular dichroism (CD) data were collected in the far-UV region on a JASCO J-810 or J-815 spectropolarimeter fitted with a Peltier temperature controller. CD spectra were acquired at 50 µM peptide solution in phosphate buffered saline (PBS; 8.2 mM sodium phosphate dibasic, 1.8 mM potassium phosphate monobasic, 137 mM sodium chloride, 2.4 mM potassium chloride), pH 7.4 at 5 °C. For pairs containing cysteine residues (i.e., pair 14 and 26), 500 µM of tris(2-carboxyethyl)phosphine (TCEP, 10 equiv. over peptides) were added to the buffer to prevent from cysteine oxidation. Data were collected in a 1 mm quartz cuvette between 190 and 260 nm and the instrument was set as follows: band width 1 nm, data pitch 1 nm, scanning speed 100 nm/min, 1 s response time. Every CD curve was obtained by averaging of 8 scans and subtracting the background signal of buffer and cuvette.

For thermal profile experiments, CD signals were monitored at 222 nm wavelength. A temperature range of 5–95 °C and a temperature ramp rate of 60 °C per hour were used with the same settings and peptide or protein concentration as above.

CD spectra were converted from ellipticities (mdeg) to mean residue ellipticities (MRE, ($deg \cdot cm^2 \cdot dmol^{-1} \cdot res^{-1}$)) by normalizing for concentration of peptide bonds and the cell path length using the equation:

$$MRE = \frac{\theta \times 10^6}{c \times l \times n}$$

where the variable θ is the measured difference in absorbed in left- and right-handed circularly polarized light in millidegrees, c is the micromolar concentration of the compound, l is the path length of the cuvette in mm, and n is the number of amide bonds in the polypeptide, for which the N-terminal acetyl bond was included but not the C-terminal amide.

1.3.3.2 Analytical ultracentrifugation

Analytical ultracentrifugation (AUC) was performed on a Beckman Optima X-LA or X-LI analytical ultracentrifuge with an An-50-Ti or An-60-Ti rotor (Beckman-Coulter). Buffer densities, viscosities, and peptide partial specific volumes ($\overline{v}$) were calculated using SEDNTERP (http://rasmb.org/sednterp/).

For sedimentation velocity, sample solutions of 310 or 410 μL were prepared in PBS (+500 μM of TCEP for pairs 14 and 26) at 50 or 140 μM peptide concentration and placed in a sedimentation velocity cell with an epon or aluminium, respectively, 2-channel centerpiece and quartz windows. The reference channel was loaded with 320 μL (or 420 μL, respectively) of PBS buffer (+500 μM of TCEP for pairs 14 and 26). The samples were centrifuged at 50 krpm at 20 °C, with absorbance scans taken across a radial range of 5.8–7.3 cm at 5 min intervals to a total of 120 scans. Data from a single run were fitted to a continuous c(s) distribution model using Sedfit,[5] at 95% confidence level. Residuals for sedimentation velocity experiments are shown as a bitmap in which the grayscale shade indicates the difference between the fit and raw data. Good fits are uniformly grey without major dark or light streaks.

Sedimentation equilibrium (SE) experiments were performed at 140 μM peptide concentration in 110 μL at 20 °C. Experiments were run in triplicate in a six- channel epon centrepiece. The samples were centrifuged at speeds in the range 24-42 krpm in increments of 3 krpm. Scans at each speed were duplicated 8 hours after the increase in speed to confirm equilibration. Data were fitted using SEDPHAT to a single species model.[6] Monte Carlo analysis was performed to give 95% confidence limits.

1.3.3.3 Native mass spectrometry (MS)

Sample solutions were prepared at 50 µM total peptide concentration in buffer of 100 mM NH_4OAc, pH 7.0 (+500 µM of TCEP for pairs 14 and 26). Pair of peptides were prepared separately as stock in NH_4OAc buffer and mixed in an equimolar ratio at 50 µM total peptide concentration. Full MS spectra were acquired on a Synapt G2S mass spectrometer using an Advion Nanomate for robot chip-based nanospray ionization in positive mode at a 1.45 kV spray voltage. Source temperature was set up at 25 °C with sampling cone set up to 20 and source offset to 120. The collision energy was turned off. Scans were recorded every second for 5 minutes. Simulated masses of the corresponding peptides were generated with the online Prot pi tool at a resolution of 18000.

1.4 X-ray crystal structure determination

Diffraction-quality peptide crystals were grown using a sitting-drop vapour-diffusion method. Freeze-dried peptides were dissolved in ultrapure water and diluted to 10-15 mg/mL. For the heterodimer pairs (9, 14, 21, and 26), peptides were first dissolved individually and then mixed in an equimolar ratio. Commercially available sparse matrix screens were used (Morpheus, JCSG-plus, Structure Screen 1 + 2, Pact Premier, ProPlex; Molecular Dimensions), and the drops were dispensed using a robot (Oryx8; Douglas Instruments). The peptides were screened in MRC 2 drop plates. Drops consisting of 0.3 µL of peptide solution and 0.3 µL of reservoir solution were screened in parallel with drops consisting of 0.4 µL of the peptide solution and 0.2 µL of reservoir solution. All plates were incubated at 20 °C. Crystals generally formed within a month, and after looping were soaked in reservoir solution containing 25% glycerol as a cryoprotectant. Crystals of CC-HP1.0 were obtained in the condition from Structure Screen 1 and 2 B6 comprising 0.2 M sodium acetate trihydrate, 0.1 M sodium cacodylate, 30% w/v PEG 8000, pH 6.5. Crystals of the heterodimer 26α/26β were obtained in the condition from JCSG-plus C11 comprising 2.0 M ammonium sulfate, 0.1 M sodium acetate, pH 4.6.

Diffraction data for the crystals were obtained at the Diamond Light Source on beamline I04. Data were processed using the automated pipelines: Xia2,[7] which ports data through DIALS[8] and AIMLESS[9] as implemented in the CCP4 suite.[10] Both structures of CC-HP1.0 and of the pair 26α/26β were solved by molecular replacement using apCC-Tet (PDB ID: 6q5s)[11] or CC-HP1.0, respectively, as models using PHASER.[12] Final structures were obtained after iterative rounds of model building with COOT and refinement with PHENIX Refine[13] or REFMAC5, respectively.[14] Solvent-exposed atoms lacking map density were either

deleted or left at full occupancy. Data collection and refinement statistics are provided in Table S2.

1.5 AlphaFold

AlphaFold2-multimer predictions were performed using the version implemented in ColabFold.[15-17] Predicted structures were relaxed using the S4 implemented Amber force fields. Predictions were colored with AlphaFold pLDDT scores using PyMol software (Schrödinger). The pLDDT score is a measure of confidence in the prediction, based on the IDDT-Cα metric[18].

1.6 Bude alanine scanning

Computational alanine scanning was performed using Bude Alanine Scan (BAlaS)[19] to identify hotspot residues at the interface of CC-HP1.0 by setting one hairpin as the 'ligand' and the other as the 'receptor'.

1.7 Sequences of Constructs used in this work

CC-HP1.0

```
tctagagggtagtggcgaactggaggcgctggccaagaaactgaaagcgctggcctggaagctgaaa
            G  E  L  E  A  L  A  K  K  L  K  A  L  A  W  K  L  K
gctctgagcaaagaaccgagcgcccaggaactggaggcgctggcccaggagctggaagcactggcc
A  L  S  K  E  P  S  A  Q  E  L  E  A  L  A  Q  E  L  E  A  L  A
aaaaagttaaaagcgctggcccagggttaggtacc
K  K  L  K  A  L  A  Q  G  *
```

CC-HP1.0-lib

```
tctagagggtagtggcgaactggaggcgctgrstaagaaanbcaaagcgctgrsttggaagnbcaaa
            G  E  L  E  A  L  A  K  K  L  K  A  L  A  W  K  L  K
gctctgagcaaagaaccgagcgcccaggaactggaggcgctgrstcaggagnbcgaagcactgrst
A  L  S  K  E  P  S  A  Q  E  L  E  A  L  A  Q  E  L  E  A  L  A
aaaaagnbcaaagcgctggcccagggttaggtacc
K  K  L  K  A  L  A  Q  G  *
```

Sequences of DNA fragments encoding CC-HP1.0 and the degenerate codon library CC-HP1.0-lib. Variable codons in the DNA library are shown using the IUPAC nucleotide codes: b=C, G or T; n=G, A, T or C; r=A or G; s=G or C.

nYFP-CC-HP1.0

MGGSHHHHHHGMASAGSGVSKGEELFTGVVPILVELDGDVNGHKFSVSGEGEGDATYGKLTLKFICTT
GKLPVPWPTLVTTFGYGLQCFARYPDHMKQHDFFKSAMPEGYVQERTIFFKDDGNYKTRAEVKFEGDT
LVNRIELKGIDFKEDGNILGHKLEYNYNSHNVYIMASGSGLEGSGELEALAKKLKALAWKLKALSKEP
SAQELEALAQELEALAKKLKALAQG

cYFP-CC-HP1.0

MGGSHHHHHHGMASAGDKQKNGIKVNFKIRHNIEDGSVQLADHYQQNTPIGDGPVLLPDNHYLSYQSA
LSKDPNEKRDHMVLLEFVTAAGITLGMDELYKTTSGSLEGSGELEALAKKLKALAWKLKALSKEPSAQ
ELEALAQELEALAKKLKALAQG

LacI*-CC-HP1.0

MGGSHHHHHHGMASMTGGQQMGRDLYDDDDKDPSSRSVKPVTLYDVAEYAGVSYQTVSRVVNQASHVS
AKTREKVEAAMAELNYIPNRVAQQLAGKQSLLIGVATSSLALHAPSQIVAAIKSRADQLGASVVVSMV
ERSGVEACKAAVHNLLAQRVSGLIINYPLDDQDAIAVEAACTNVPALFLDVSDQTPINSIIFSHEDGT
RLGVEHLVALGHQQIALLAGPLSSVSARLRLAGWHKYLTRNQIQPIAEREGDWSAMSGFQQTMQMLNE
GIVPTAMLVANDQMAAGAMRAITESGLRVGADISVVGYDDTEDSSCYIPPLTTIKQDFRLLGQTSVDR
LLQLSQGQAVKGNQLLPVSLVKRKTTLAPGLEGSGELEALAKKLKALAWKLKALSKEPSAQELEALAQ
ELEALAKKLKALAQG

Amino acid sequences of the fusion proteins used in this work. Green highlights the His-tag, yellow the split YFP fragments (nYFP and cYFP), cyan is CC-HP1.0 and grey highlights LacI*.

SUPPLEMENTARY DATA

2.1 Supplementary Figures

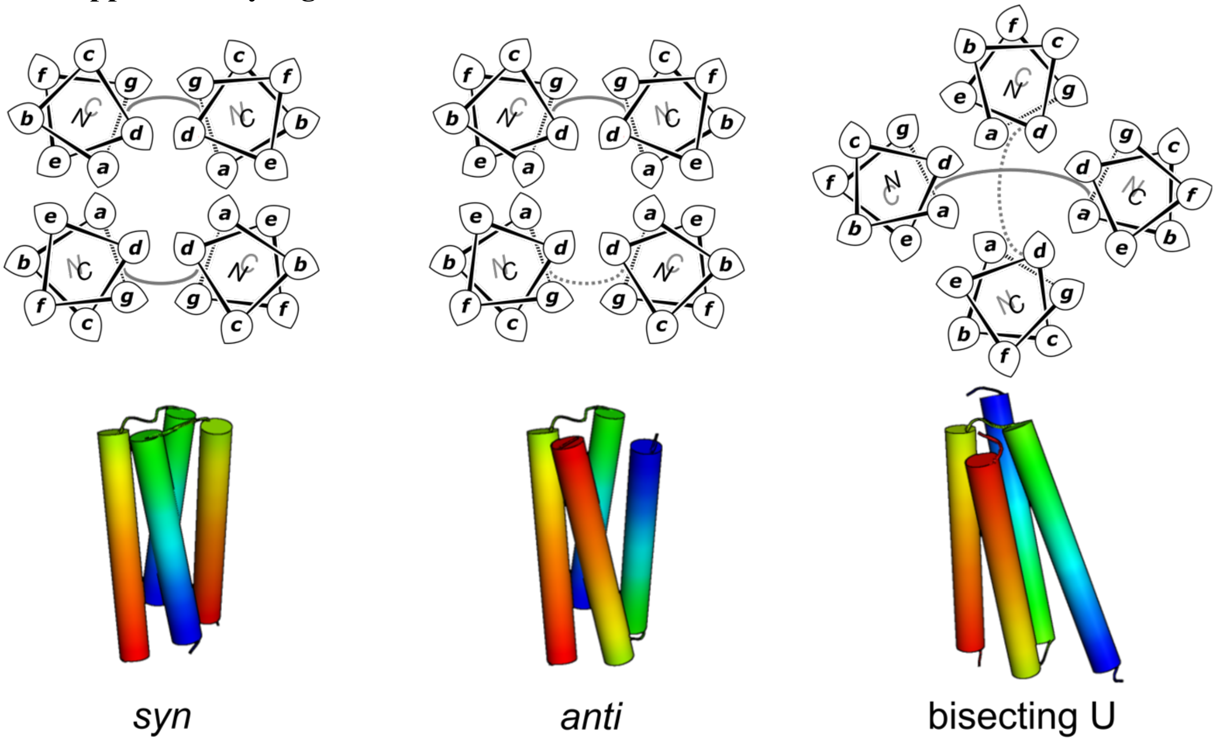

Figure S1. Representation of the different possible topologies for a dimer of hairpins (helix-loop-helix). Left: helical wheel (top) and model structure (bottom) of the *syn* topology, in which the loops are at the same end of the quaternary assembly. Middle: helical wheel (top) and model structure (bottom) of the *anti* topology, in which the loops are at the opposite ends of the quaternary assembly. Right: helical wheel (top) and model structure (bottom) of the bisecting-U topology, in which the hairpins interdigitate and the loops are at opposite ends of the quaternary assembly. In the helical wheel, the N-to-C-terminal directions of the helices are indicated with the 'N' or 'C' with the darker font indicating that end is closer to the viewer. The chains of the model structures are colored in chainbow from the N (blue) to the C termini (red).

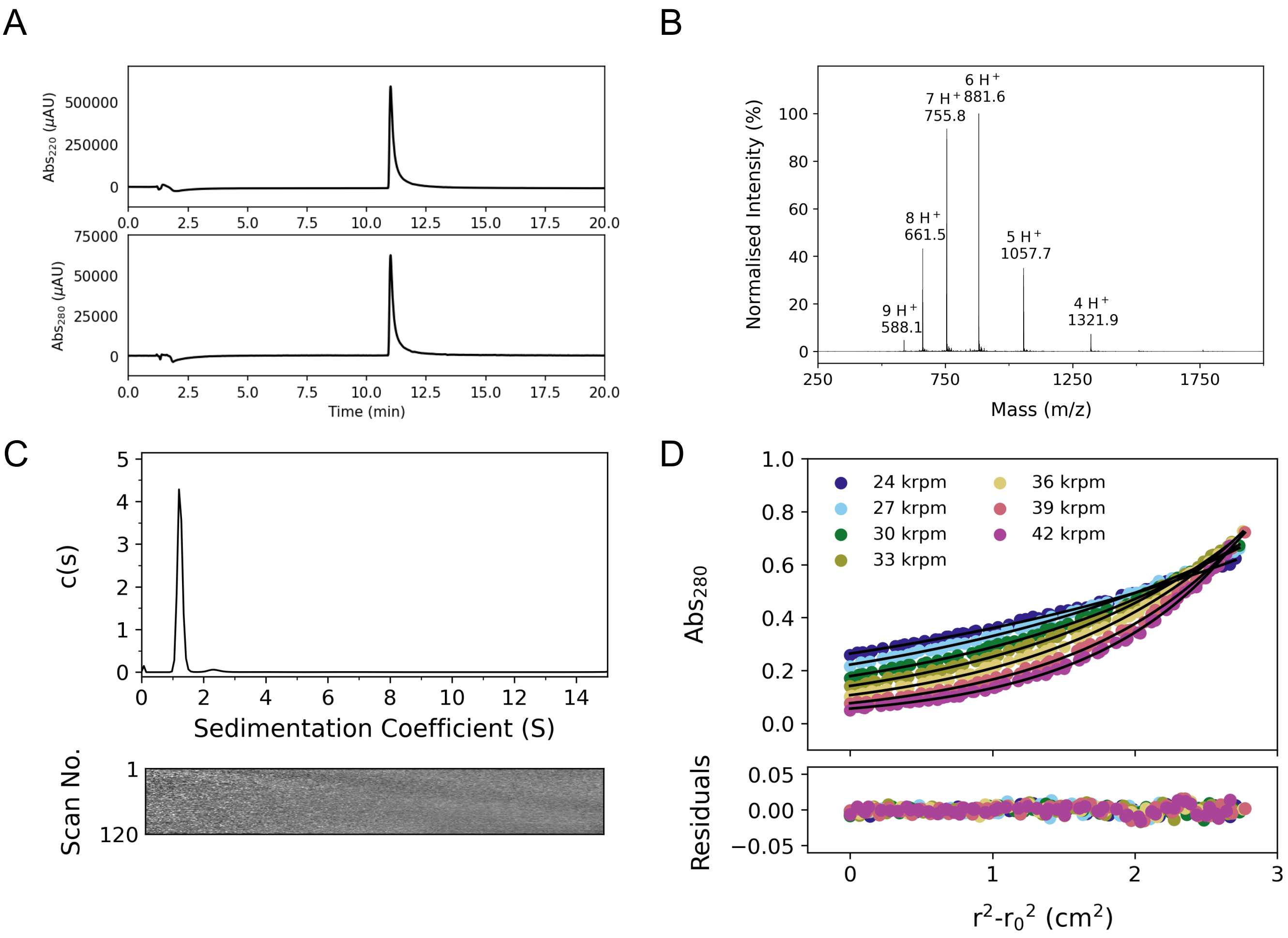

Figure S2. Characterization of the peptide CC-HP1.0. (A) Analytical HPLC chromatogram of CC-HP1.0 at 220 nm (top) and 280 nm (bottom) from a gradient of 20 to 80% MeCN (0.1% TFA) in H_2O (0.1% TFA). (B) ESI MS of CC-HP1.0. Calculated mass: 5283.3 [M]. Deconvoluted observed mass: 5383.7 Da [M]. (C) Sedimentation velocity (SV) data of the peptide CC-HP1.0 ($\bar{v}$ = 0.769 cm^3 g^{-1}). Continuous c(s) distribution returned a molecular mass of 11138 Da corresponding to 2.1 x monomer mass at 95% confidence level (f/f_0 = 1.170, s = 1.241 S, $s_{20,w}$ = 1.292 S). (D) Sedimentation equilibrium (SE) AUC data for CC-HP1.0 between 24 and 42 krpm at 3 krpm intervals. Fitted single-ideal species model curves are overlaid in black and gave a molecular mass 11027 Da corresponding to 2.1 x monomer mass, 95% confidence limits 10965-11156 Da. Conditions: 140 µM peptide, PBS, pH 7.4, 20 °C.

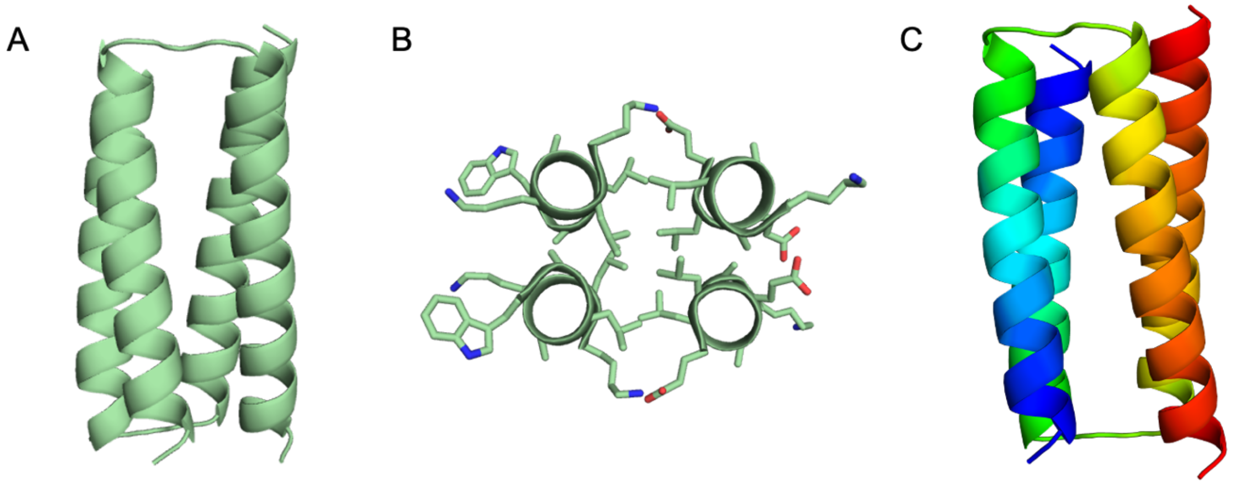

Figure S3. Orthogonal views of the X-ray crystal structure of CC-HP1.0 at 2.1 Å resolution (PDB ID, 8bcs; Table S1). (A) Cartoon representation of the dimer of hairpins, which crystalized in the *anti* conformation. (B) Cross section through one heptad layer showing the knobs-into-holes packing. (C) Chainbow representation of CC-HP1.0 with each chain colored from the (*N* terminus (blue) to the *C* terminus (red).

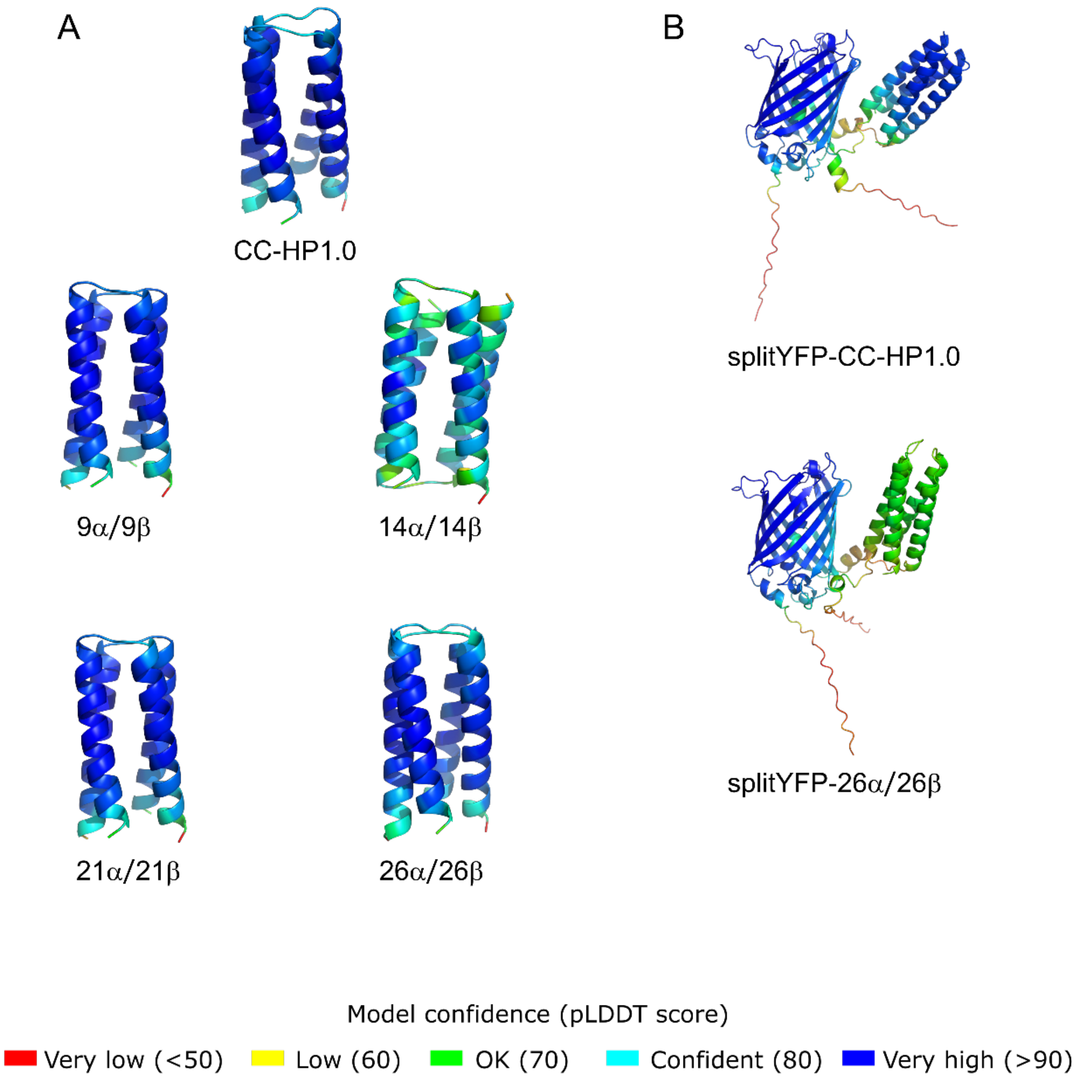

Figure S4. (A) AlphaFold2 multimer models of parent CC-HP1.0 and the 4 heterodimer pairs selected from this screen. (B) AlphaFold2 multimer models of splitYFP fused to CC-HP1.0 and 26α/26β. Structures are colored using AlphaFold pLDDT scores as B factors with the color key showing the model confidence.

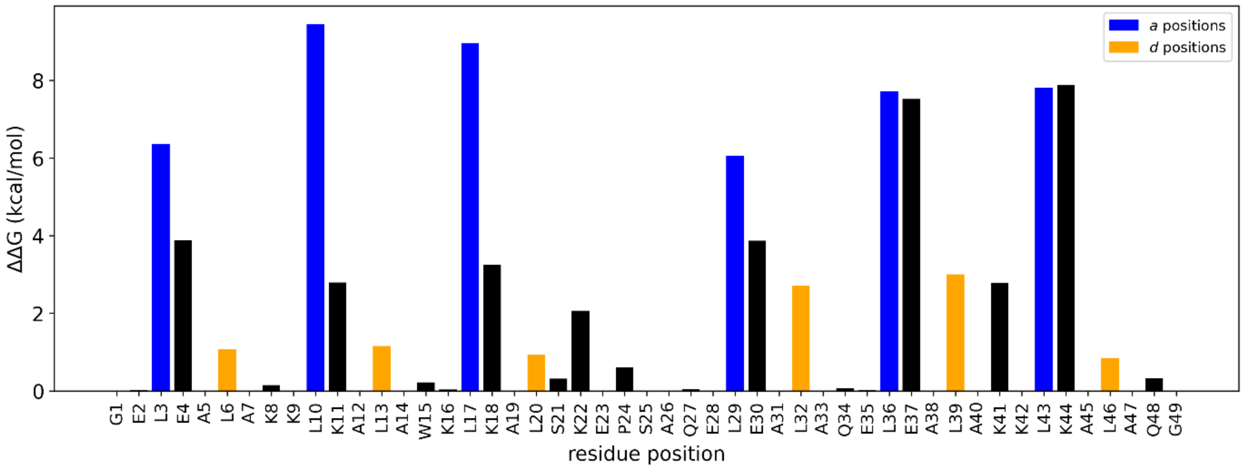

Figure S5. Bar chart showing the free energy contributions of individual residues in CC-HP1.0 to the hairpin interface of the homodimer. This was calculated using computational alanine scanning, performed using Bude Alanine Scan (BAlaS). The free energy contributions at the ***a*** positions are shown in blue and those at the ***d*** positions are shown in orange. All other residues are shown in black.

.

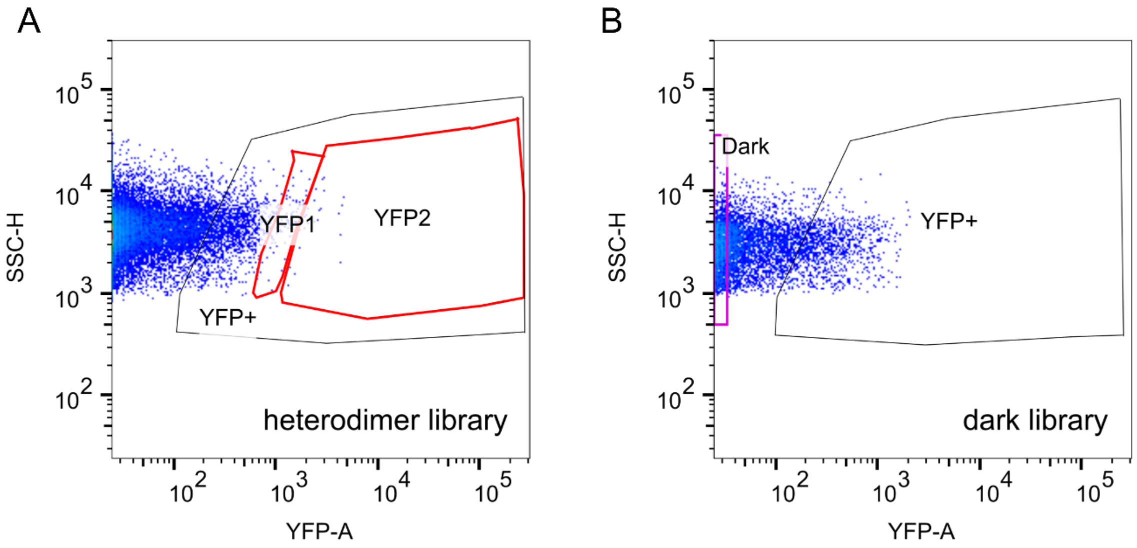

Figure S6. Representative FACS plots showing split-YFP fusions of CC-HP1.0-lib expressed in (A) OmniMAX™ and (B) TB28 cells. Side scatter (SSC) is plotted against YFP fluorescence. OmniMAX™ cells were used to select the "bright" heterodimer library candidates (gates shown with red outline) and the TB28 cells were used to select "dark" library candidates for sequencing (gate shown with magenta outline).

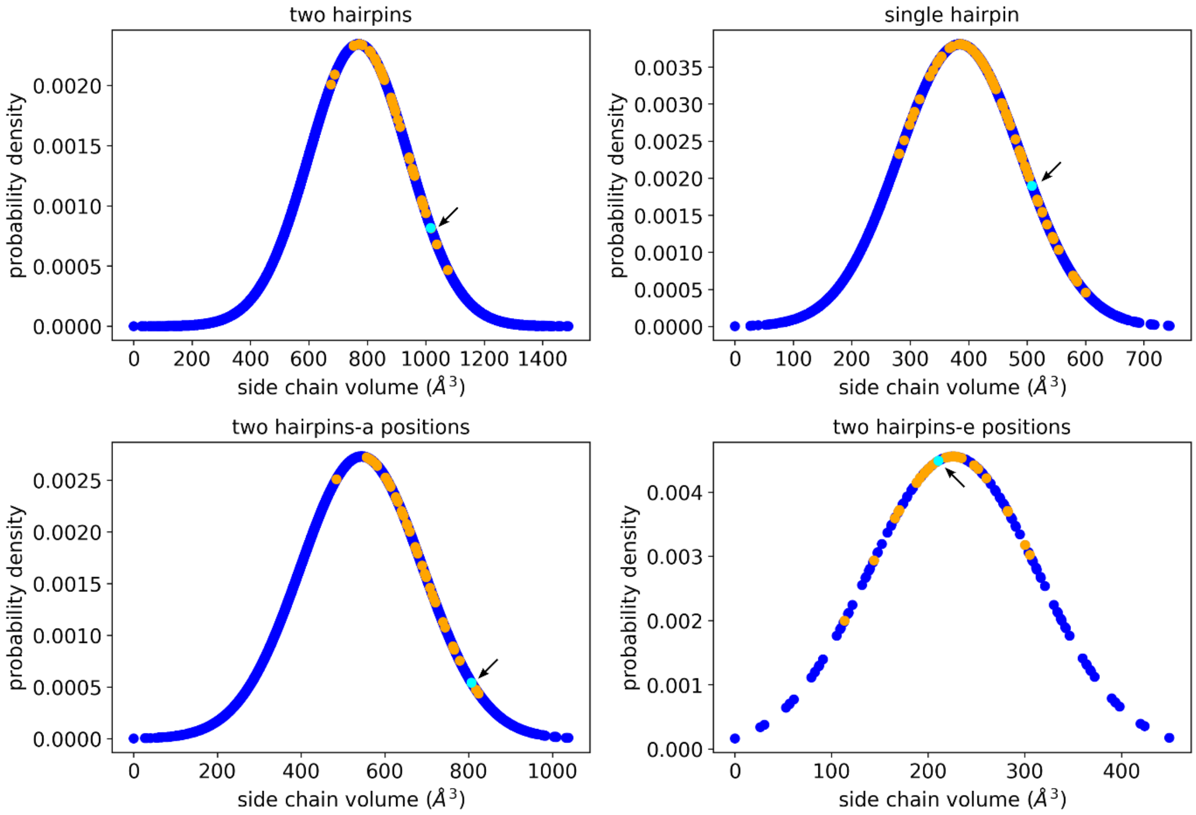

Figure S7. Statistical analysis of interacting hairpins selected from the library. Calculated normal distributions of the summed side-chain volumes at the varied ***a*** and ***e*** positions for all the possible library variants in the combinations indicated (colored blue). Corresponding values for the sequences of the selected putative heterodimers are overlaid on these curves and shown in orange. The values for CC-HP1.0 are plotted in cyan and indicated with an arrow. The values for the heterodimers, dark library and CC-HP1.0 are shown plotted on the normal-distribution curve to ease comparisons only; however, only the x-axis values are relevant for these points.

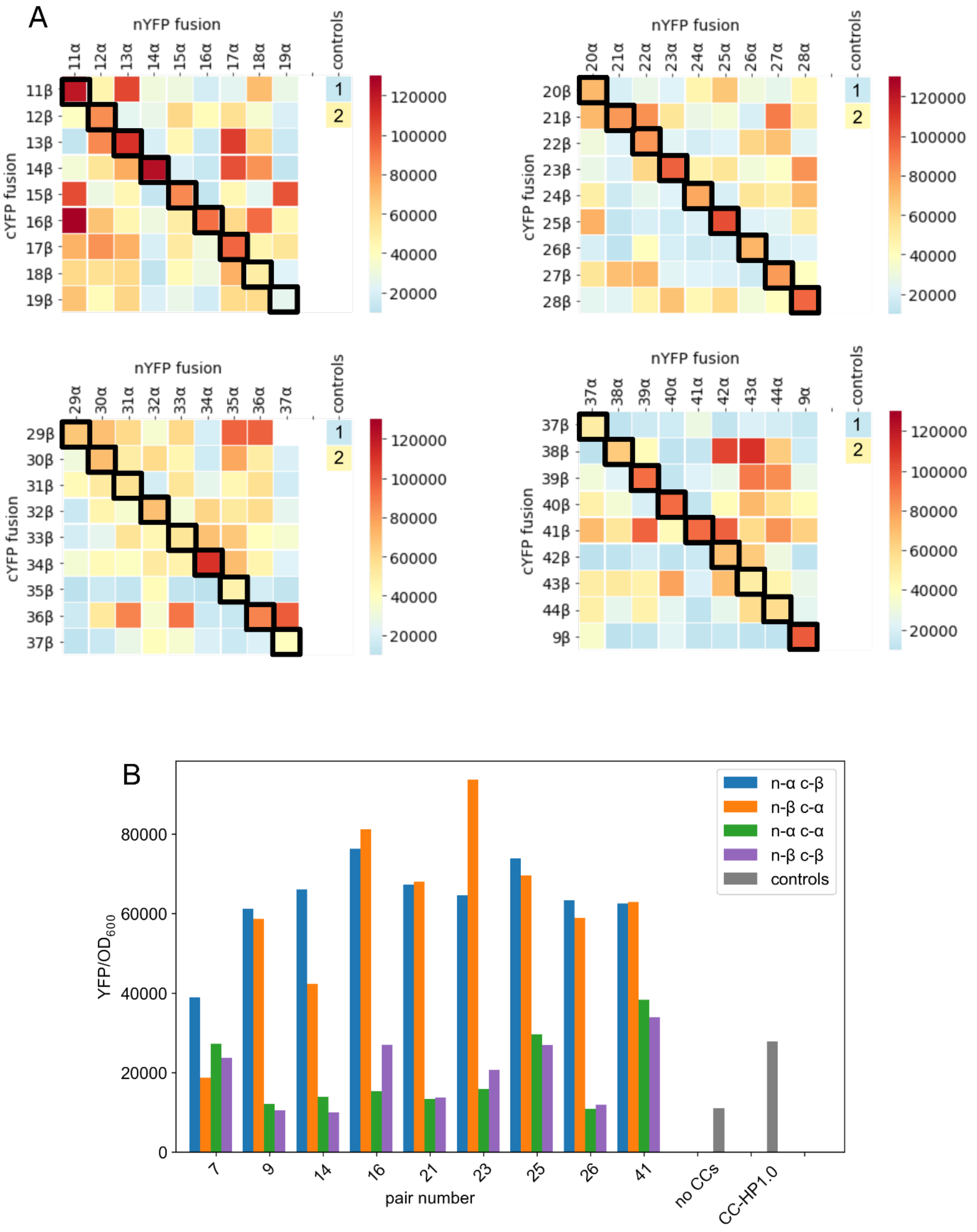

Figure S8. Identifying hairpins from the library screen that can heterodimerize but not homodimerize. (A) Further heatmaps showing split-YFP reconstitution between different hairpins fused to nYFP(α) and cYFP The numbering scheme simply refers to pairs isolated from the library screen, with the co-selected pairs highlighted by black boxes. Each hairpin was tested against 9 other hairpins in the combinations shown. The scale shows

YFP fluorescence normalized to the OD_{600} of the bacterial cell culture. Controls are labelled 1 for no coils and 2 for the parent CC-HP1.0. (B) Bar chart showing YFP fluorescence of the selected heterodimer candidates. Each hairpin from the heterodimer pair was fused to nYFP and cYFP in the combinations shown. Blue and orange bars show heterodimer interactions between the α and β hairpins, and green and purple bars show homo-dimerization of two α or two β hairpins, respectively. Transformations and assays were carried out in 96 well plates and YFP fluorescence was normalized to the OD_{600} of the bacterial cell culture.

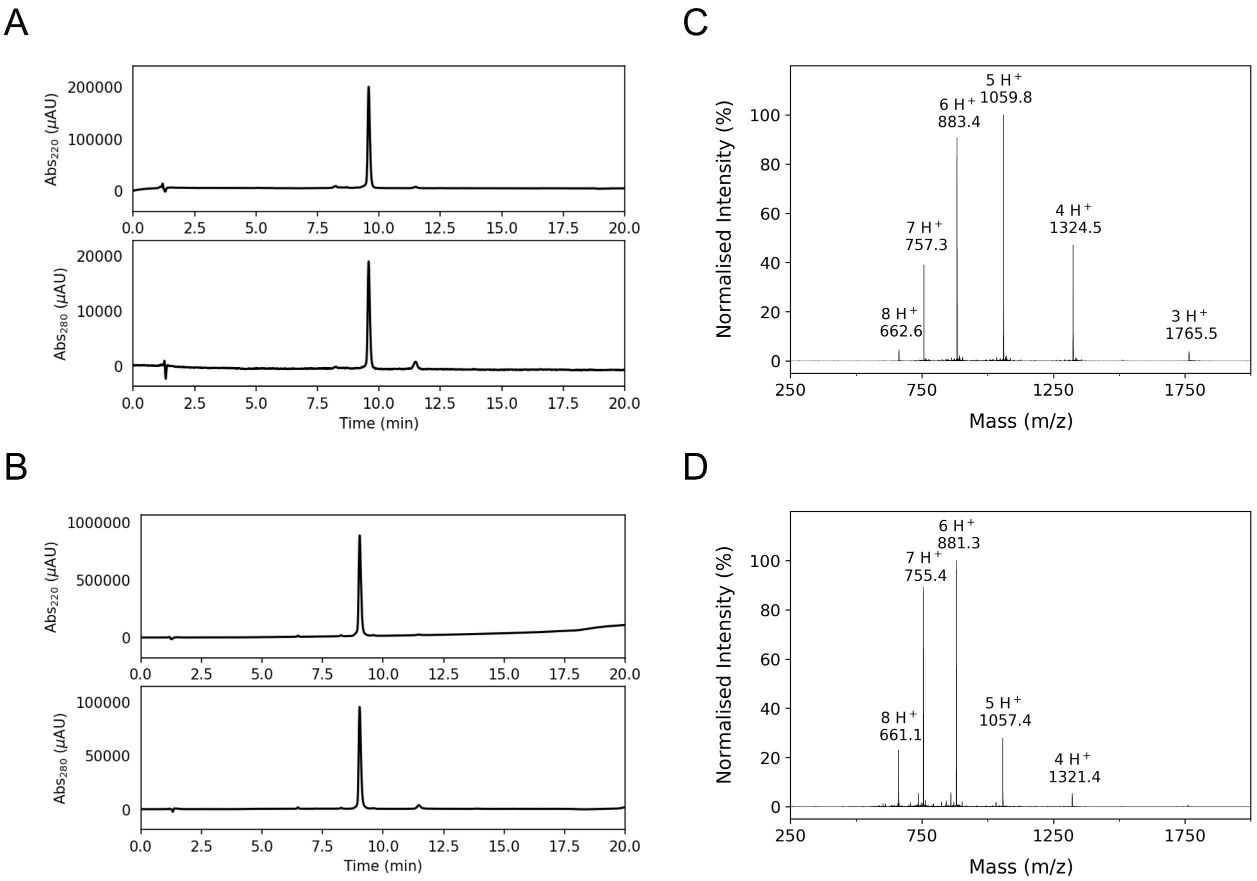

Figure S9. Characterization of the peptides 9α and 9β. (A-B) Analytical HPLC chromatogram of 9α (A) and 9β (B) at 220 nm (top) and 280 nm (bottom) from a gradient of 20 to 80% MeCN (0.1% TFA) in H_2O (0.1% TFA). (C-D) ESI MS of 9α (C) and 9β (D). (C) Calculated mass of 9α: 5293.2 [M]. Deconvoluted observed mass of 9α: 5294.1 Da [M]. (D) Calculated mass of 9β: 5281.2 Da [M]. Deconvoluted observed mass of 9β: 5281.8 Da [M].

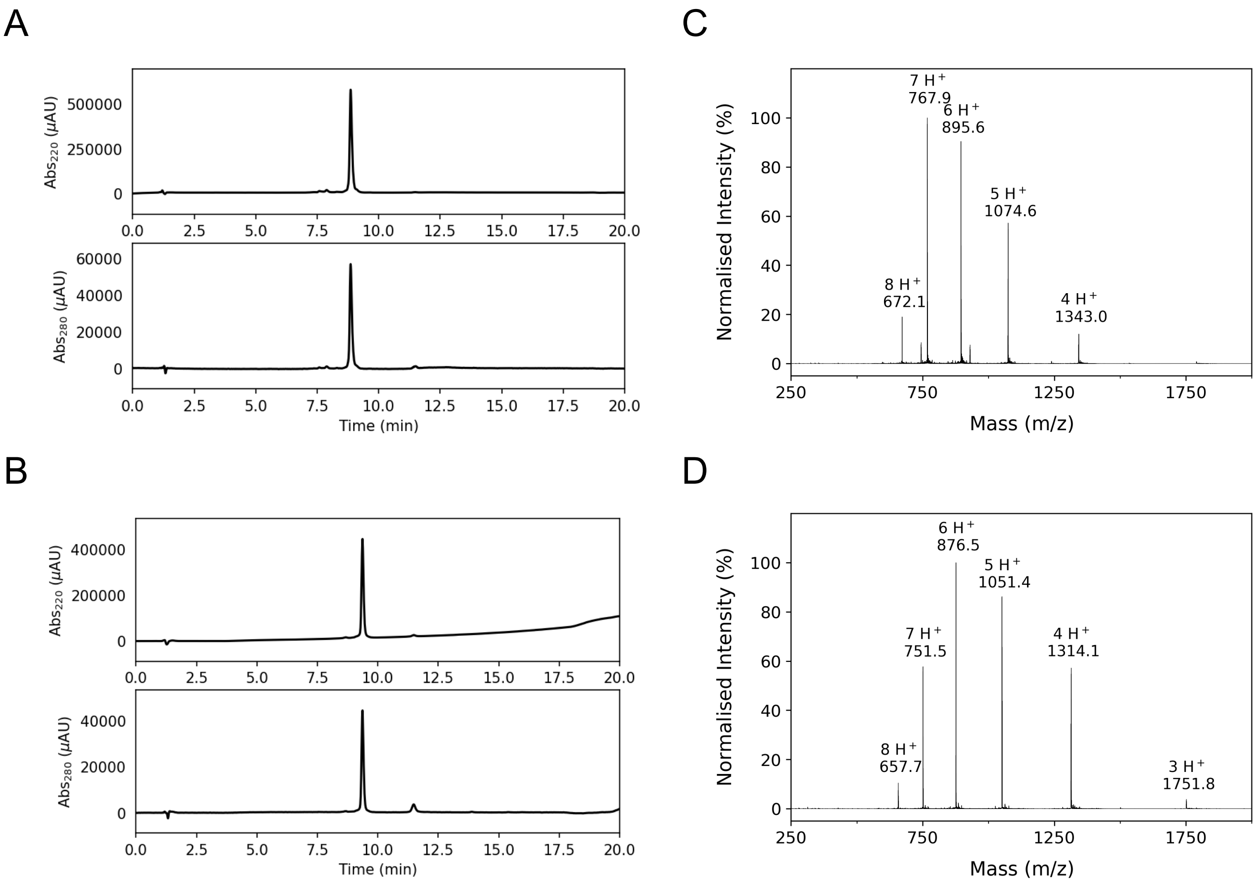

Figure S10. Characterization of the peptides 14α and 14β. (A-B) Analytical HPLC chromatogram of 14α (A) and 14β (B) at 220 nm (top) and 280 nm (bottom) from a gradient of 20 to 80% MeCN (0.1% TFA) in H_2O (0.1% TFA). (C-D) ESI MS of 14α (C) and 14β (D). (C) Calculated mass of 14α: 5368.3 [M]. Deconvoluted observed mass of 14α: 5368.4 Da [M]. (D) Calculated mass of 14β: 5252.2 Da [M]. Deconvoluted observed mass of 14β: 5253.0 Da [M].

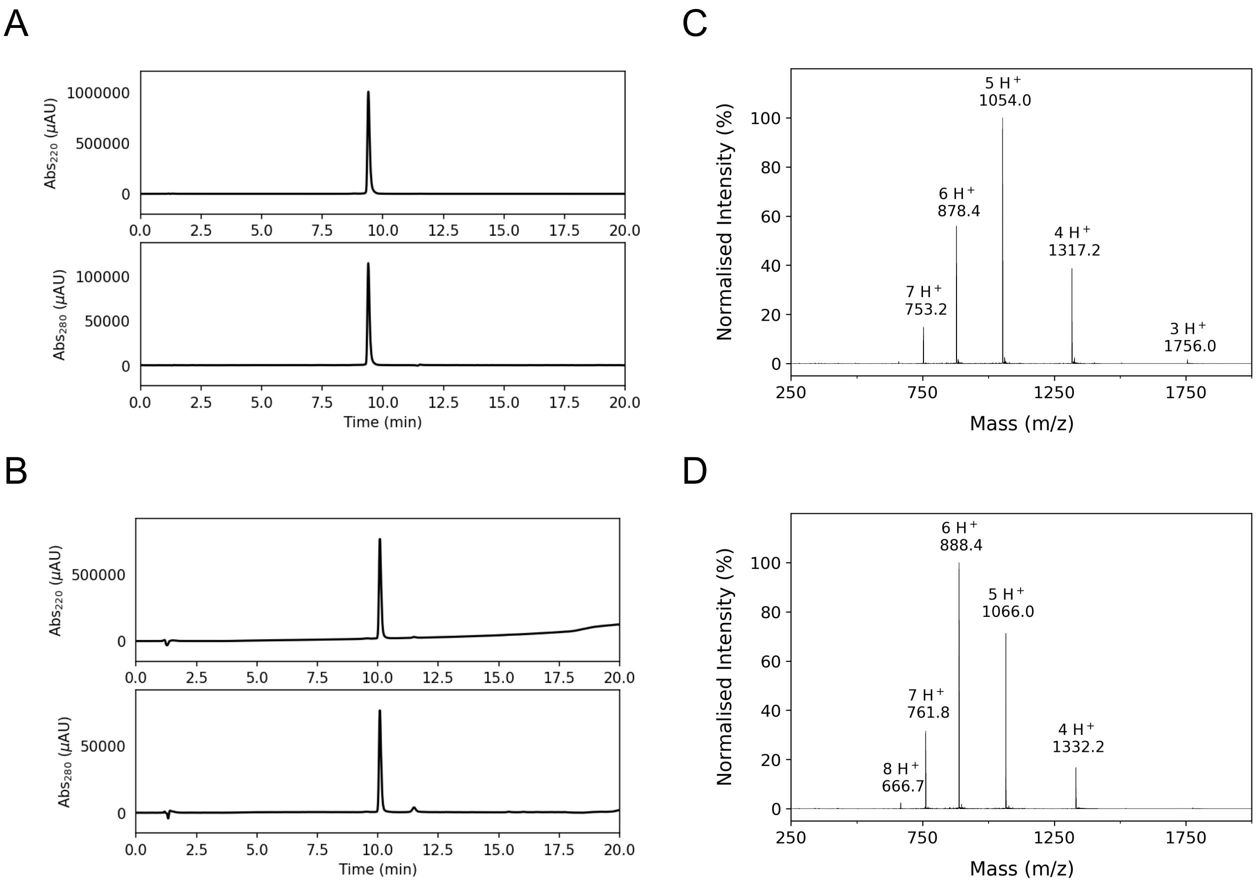

Figure S11. Characterization of the peptides 21α and 21β. (A-B) Analytical HPLC chromatogram of 21α (A) and 21β (B) at 220 nm (top) and 280 nm (bottom) from a gradient of 20 to 80% MeCN (0.1% TFA) in H_2O (0.1% TFA). (C-D) ESI MS of 21α (C) and 21β (D). (C) Calculated mass of 21α: 5265.2 [M]. Deconvoluted observed mass of 21α: 5265.2 Da [M]. (D) Calculated mass of 21β: 5325.3 Da [M]. Deconvoluted observed mass of 21β: 5325.3 Da [M].

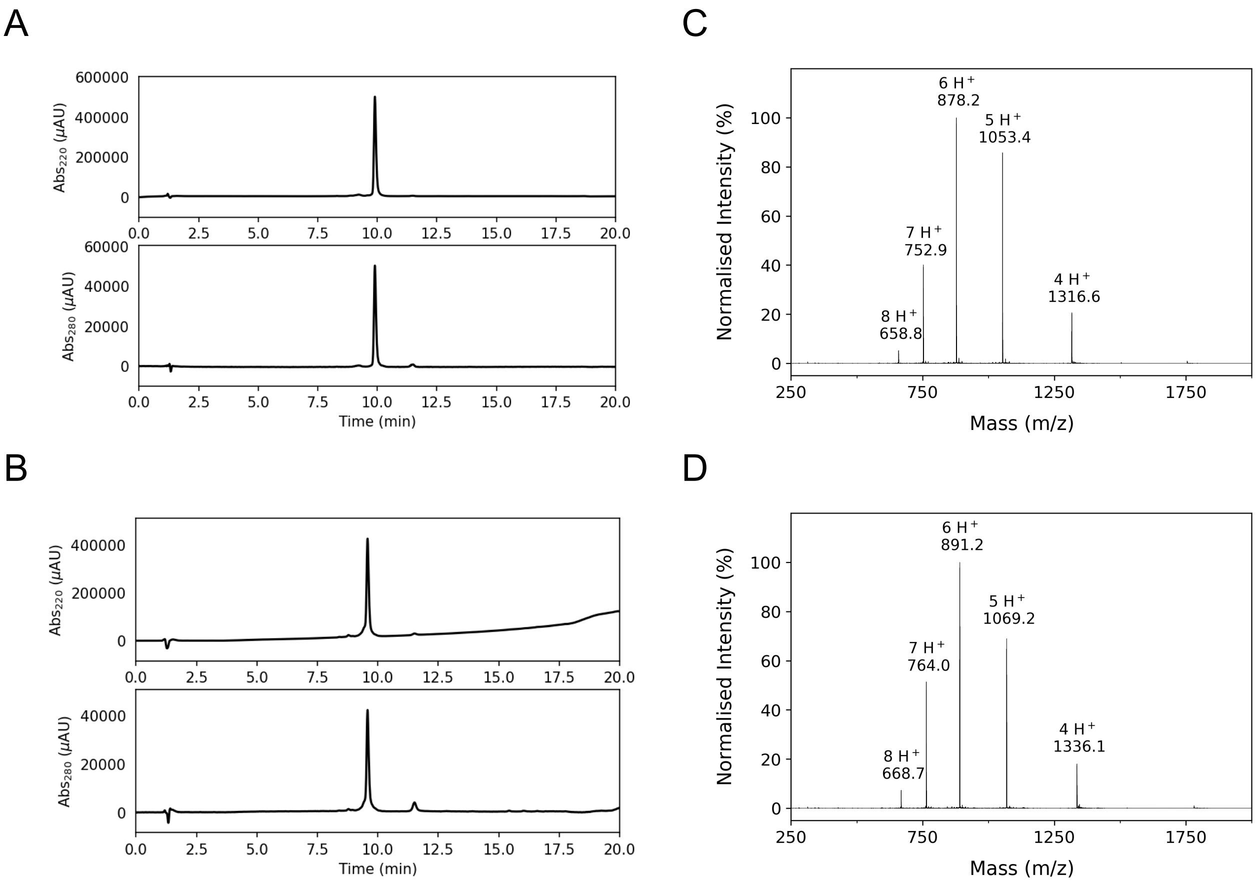

Figure S12. Characterization of the peptides 26α and 26β. (A-B) Analytical HPLC chromatogram of 26α (A) and 26β (B) at 220 nm (top) and 280 nm (bottom) from a gradient of 20 to 80% MeCN (0.1% TFA) in H_2O (0.1% TFA). (C-D) ESI MS of 26α (C) and 26β (D). (C) Calculated mass of 26α: 5263.2 [M]. Deconvoluted observed mass of 26α: 5263.0 Da [M]. (D) Calculated mass of 26β: 5340.3 Da [M]. Deconvoluted observed mass of 26β: 5340.9 Da [M].

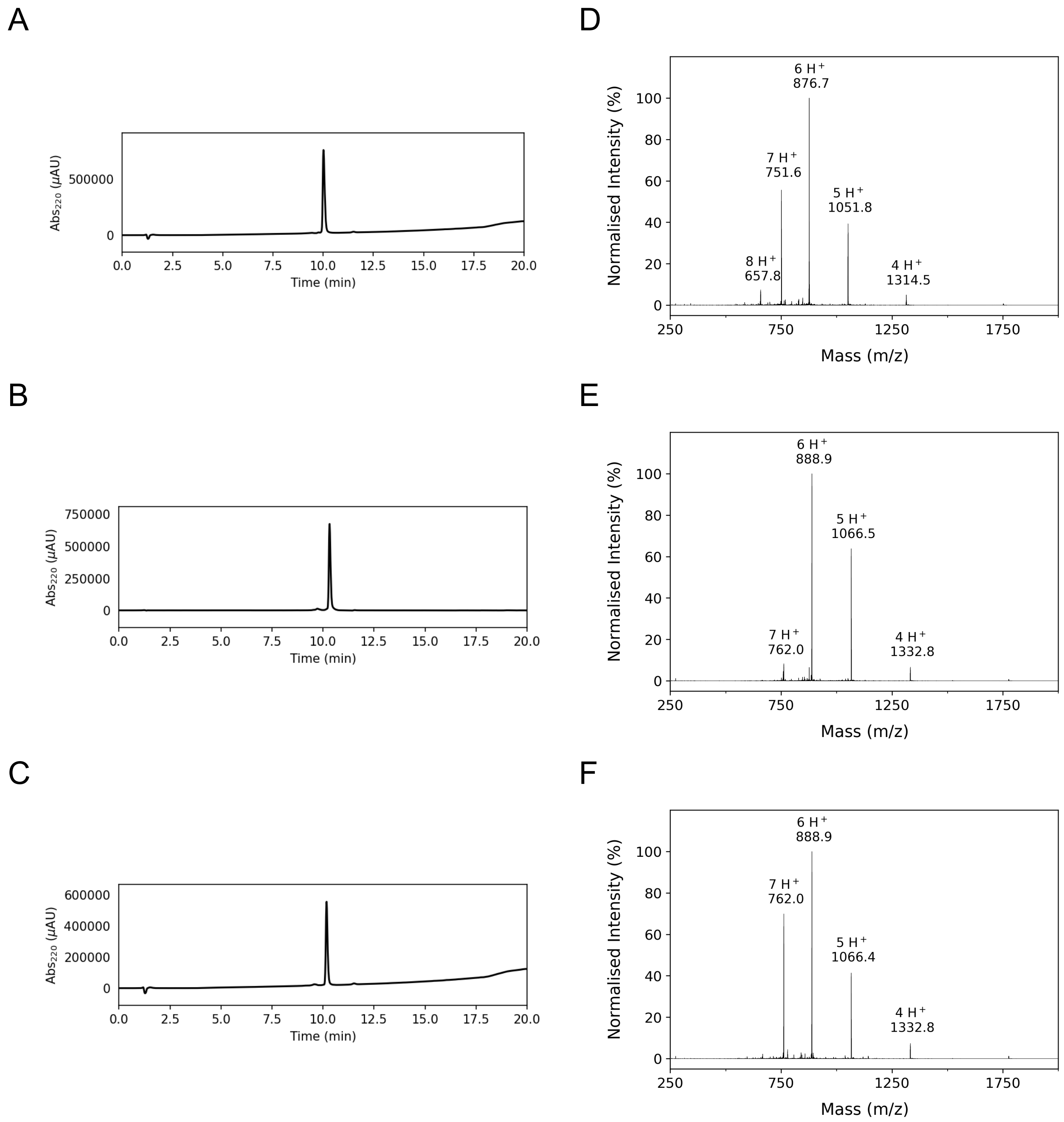

Figure S13. Characterization of the peptides 26α-nMSE, 26β-c4CF and 26β-n4CF. (A-C) Analytical HPLC chromatogram of 26α-nMSE (A), 26β-c4CF (B) and 26β-n4CF (C) at 220 nm from a gradient of 20 to 80% MeCN (0.1% TFA) in H_2O (0.1% TFA). (D-F) ESI MS of 26α-nMSE (D), 26β-c4CF (E) and 26β-n4CF (F) . (D) Calculated mass of 26α-nMSE: 5254.1 [M]. Deconvoluted observed mass of 26α-nMSE: 5254.1 Da [M]. (E) Calculated mass of 26β-c4CF: 5327.2 Da [M]. Deconvoluted observed mass of 26β-c4CF: 5327.3 Da [M]. (F) Calculated mass of 26β-n4CF: 5327.2 Da [M]. Deconvoluted observed mass of 26β-n4CF: 5326.2 Da [M].

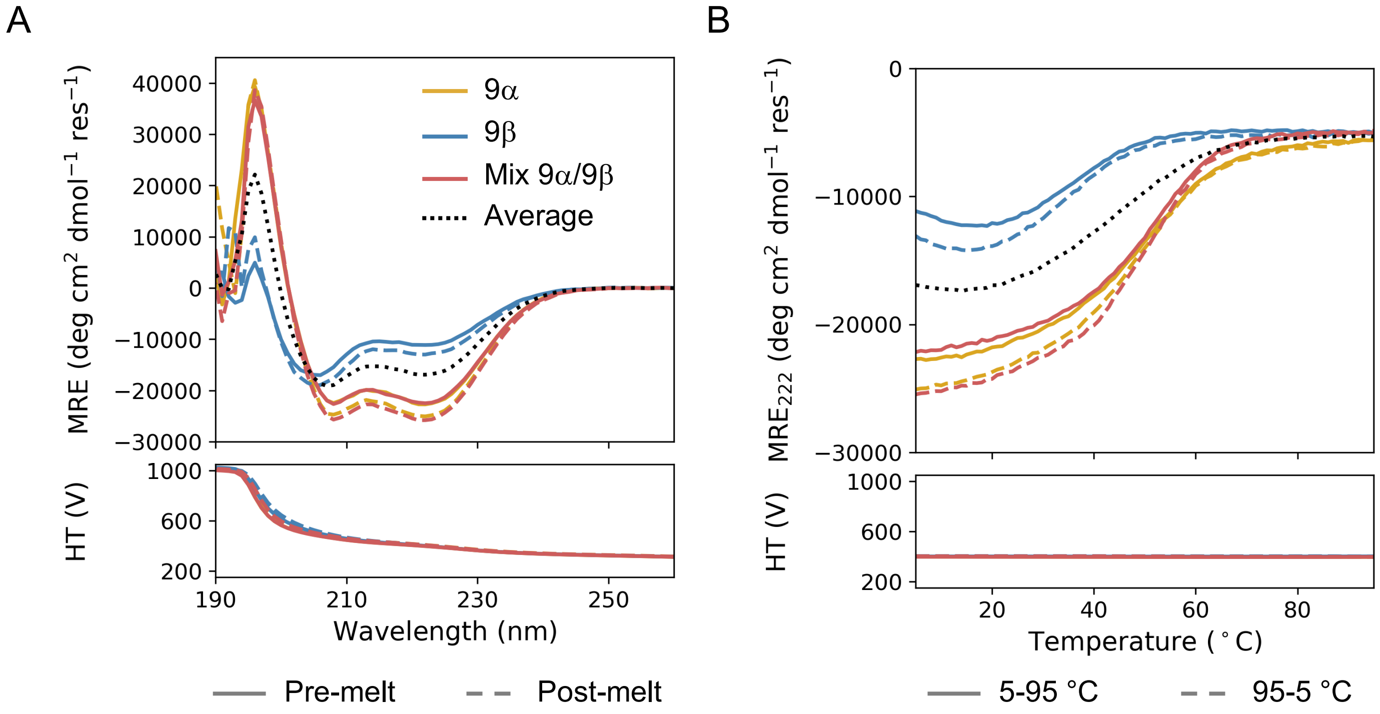

Figure S14. CD spectroscopy data for the peptides of pair 9. (A) CD spectra at 5 °C before (solid lines) and after (dashed lines) thermal denaturation for the peptides 9α (yellow) and 9β (blue) alone and the mixture of both peptides 9α/9β (red). The calculated average of 9α and 9β spectra is represented with a black dotted line. (B) Thermal unfolding (solid lines) and refolding (dashed lines) monitored at 222 nm wavelength. Color codes are the same as (A). Second derivatives of melting curves return melting temperature of 51 °C for 9α, 36 °C for 9β and 52 °C for the mixture 9α/9β. Conditions: 50 µM total concentration of peptides, PBS buffer, pH 7.4.

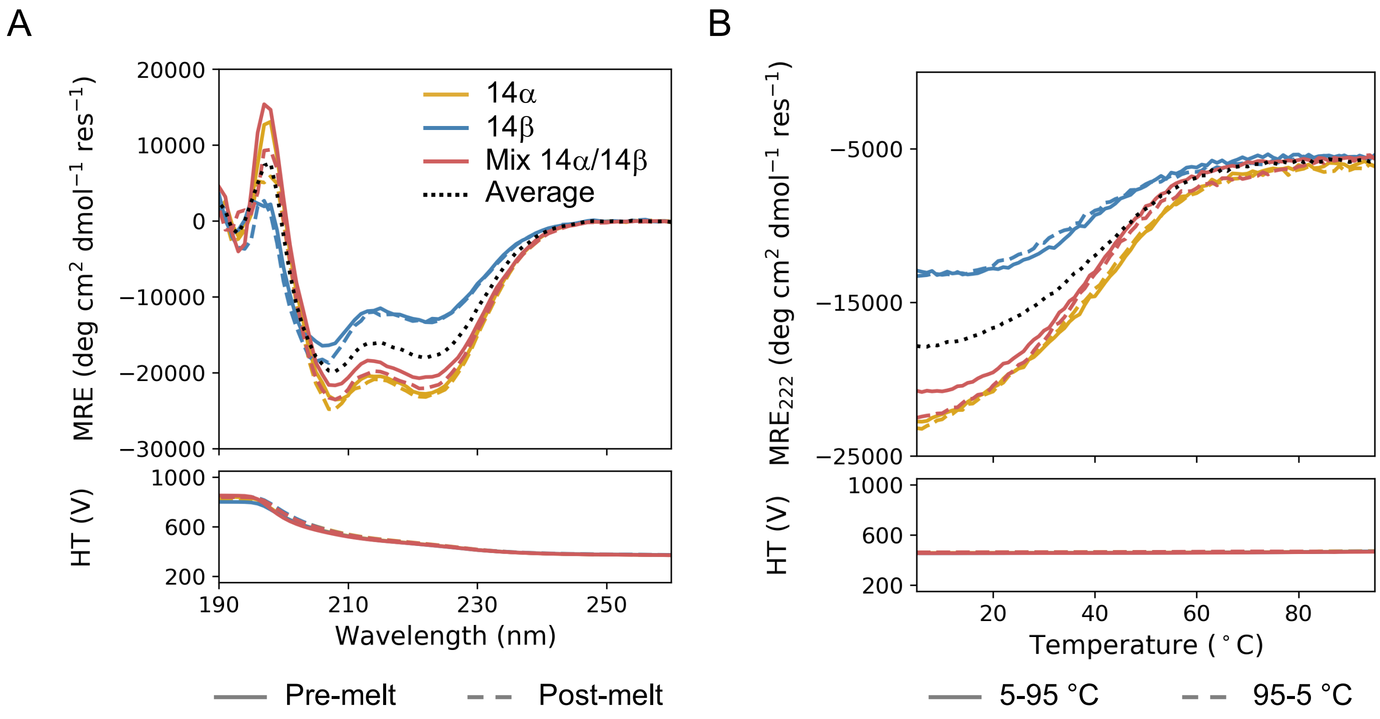

Figure S15. CD spectroscopy data for the peptides of pair 14. (A) CD spectra at 5 °C before (solid lines) and after (dashed lines) thermal denaturation for the peptides 14α (yellow) and 14β (blue) alone and the mixture of both peptides 14α/14β (red). The calculated average of 14α and 14β spectra is represented with a black dotted line. (B) Thermal unfolding (solid lines) and refolding (dashed lines) monitored at 222 nm wavelength. Color codes are the same as (A). Second derivatives of melting curves return melting temperature of 44 °C for 14α, 39 °C for 14β and 40 °C for the mixture 14α/14β. Conditions: 50 µM total concentration of peptides, PBS buffer, 500 µM TCEP, pH 7.4.

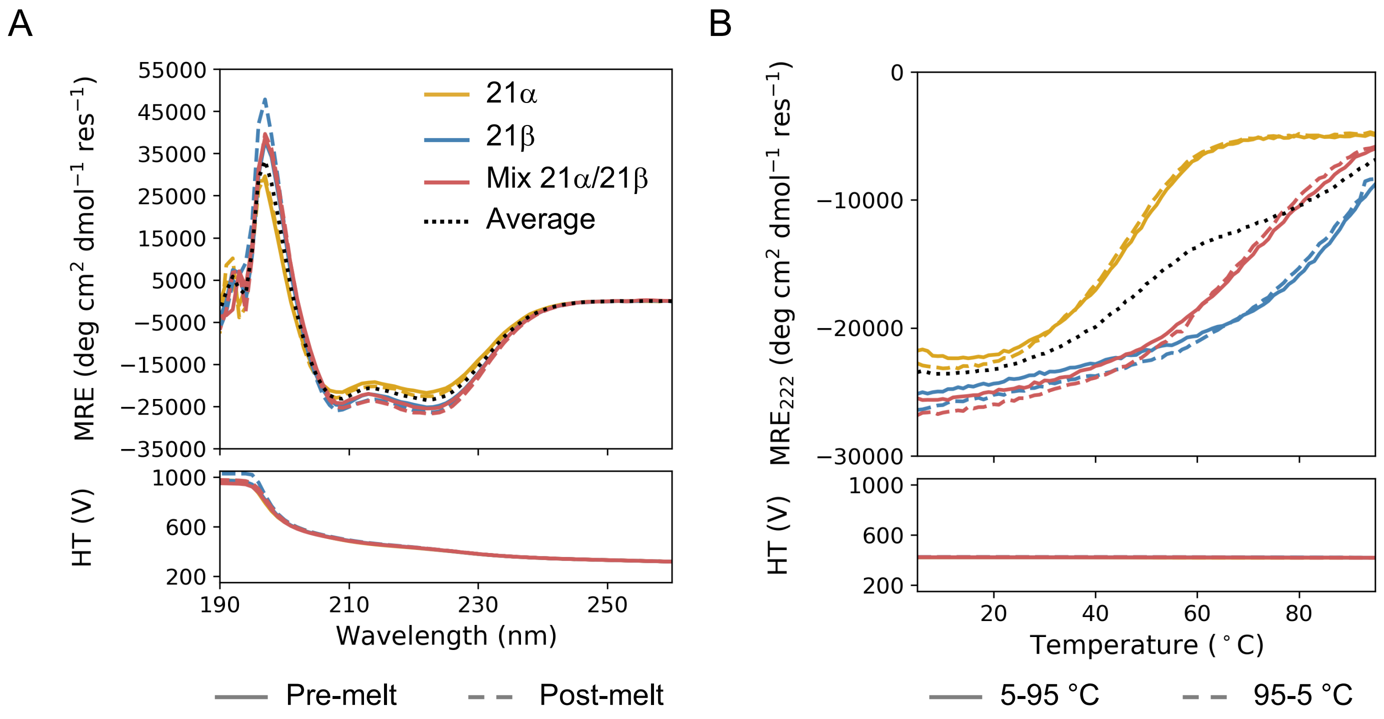

Figure S16. CD spectroscopy data for the peptides of pair 21. (A) CD spectra at 5 °C before (solid lines) and after (dashed lines) thermal denaturation for the peptides 21α (yellow) and 21β (blue) alone and the mixture of both peptides 21α/21β (red). The calculated average of 21α and 21β spectra is represented with a black dotted line. (B) Thermal unfolding (solid lines) and refolding (dashed lines) monitored at 222 nm wavelength. Color codes are the same as (A). Second derivatives of melting curves return melting temperature of 48 °C for 21α, not determined for 21β and 72 °C for the mixture 21α/21β. Conditions: 50 µM total concentration of peptides, PBS buffer, pH 7.4.

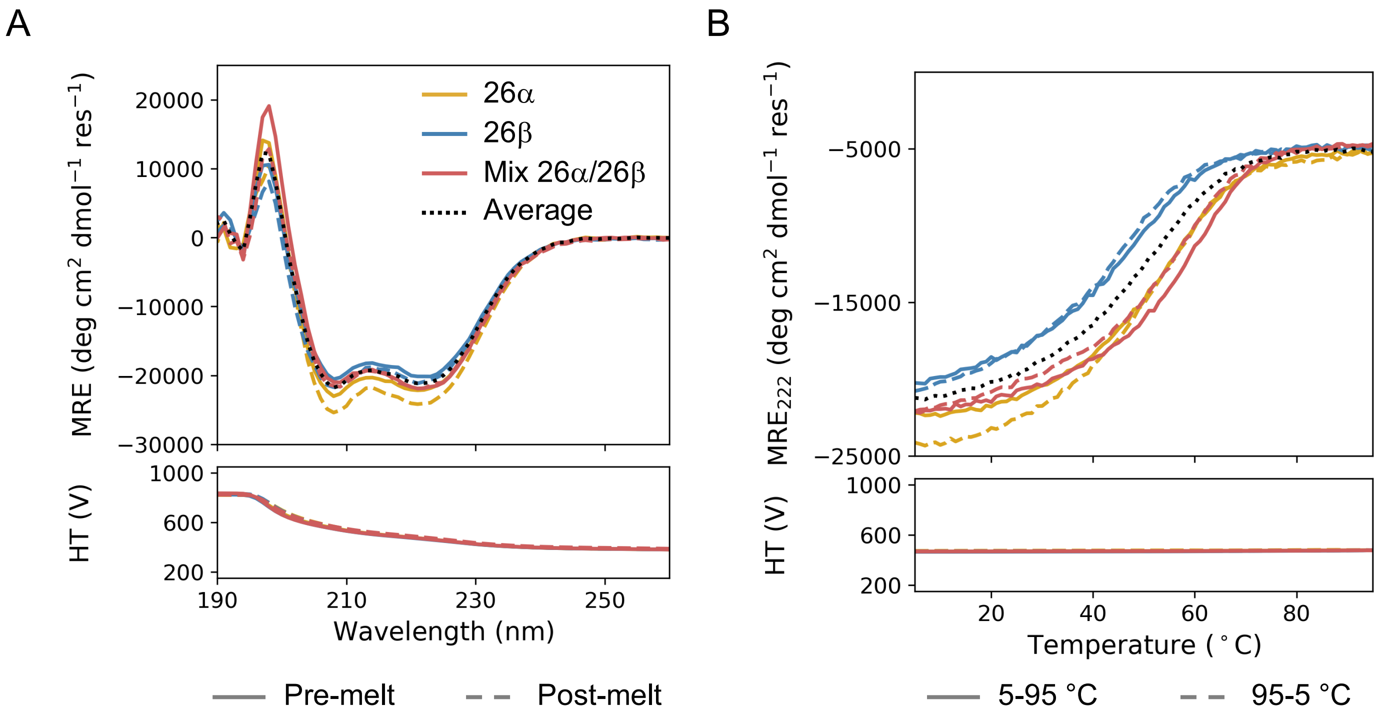

Figure S17. CD spectroscopy data for the peptides of pair 26. (A) CD spectra at 5 °C before (solid lines) and after (dashed lines) thermal denaturation for the peptides 26*α* (yellow) and 26*β* (blue) alone and the mixture of both peptides 26*α*/26*β* (red). The calculated average of 26*α* and 26*β* spectra is represented with a black dotted line. (B) Thermal unfolding (solid lines) and refolding (dashed lines) monitored at 222 nm wavelength. Color codes are the same as (A). Second derivatives of melting curves return melting temperature of 55 °C for 26*α*, 48 °C for 26*β* and 60 °C for the mixture 26*α*/26*β*. Conditions: 50 µM total concentration of peptides, PBS buffer, 500 µM TCEP, pH 7.4.

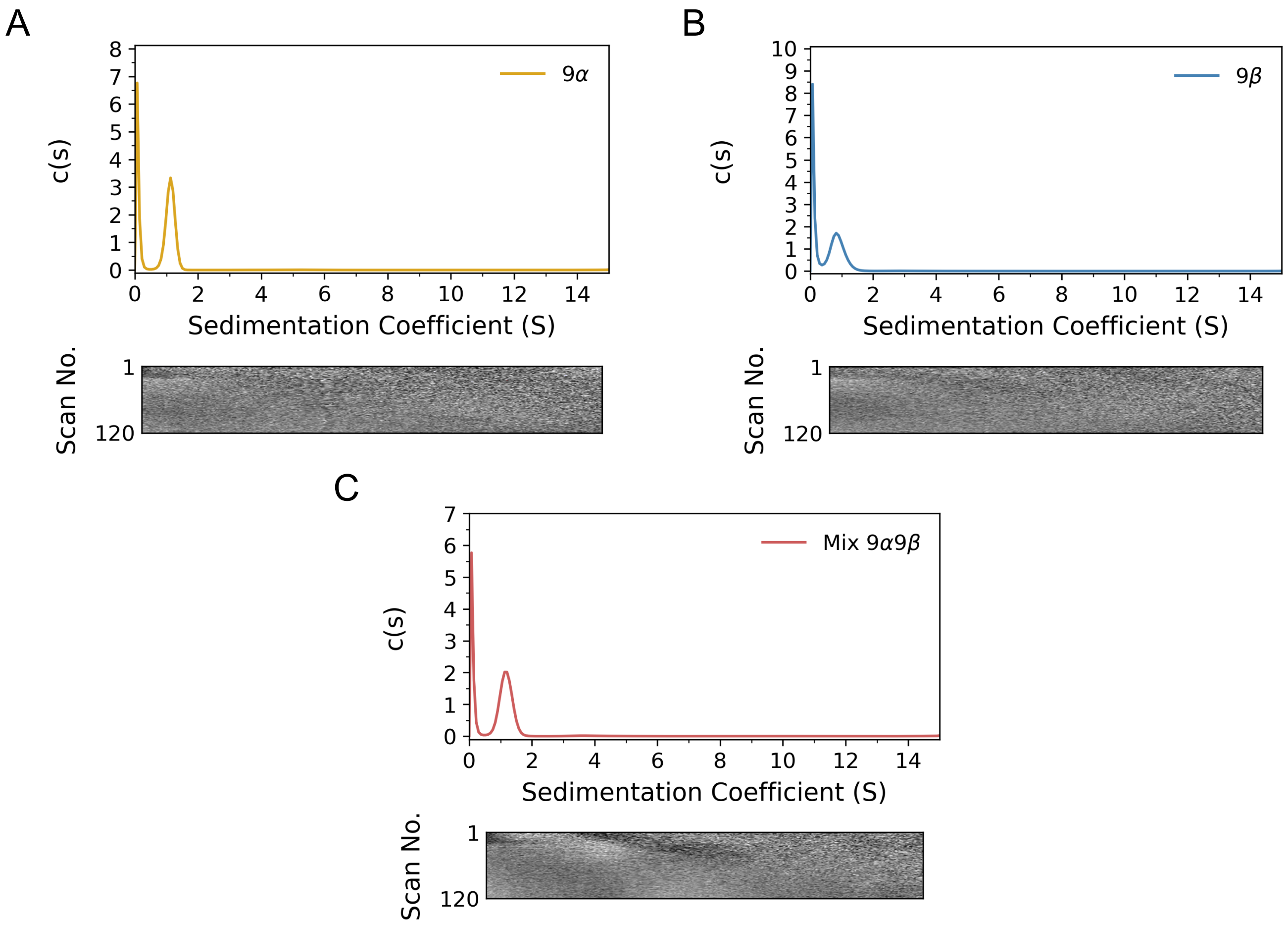

Figure S18. Sedimentation velocity (SV) AUC data for the the peptides of pair 9 at 50 krpm. Residuals are shown as a bitmap below the fitted data. (A) SV data of the peptide 9α ($\bar{v}$ = 0.764 cm^3 g^{-1}). Continuous c(s) distribution returned a molecular mass of 9913 Da corresponding to 1.9 x monomer mass at 95% confidence level (f/f_0 = 1.232, s = 1.120 S, $s_{20,w}$ = 1.165 S). (B) SV data of the peptide 9β ($\bar{v}$ = 0.760 cm^3 g^{-1}). Continuous c(s) distribution returned a molecular mass of 8697 Da corresponding to 1.7 x monomer mass at 95% confidence level (f/f_0 = 1.471, s = 0.877 S, $s_{20,w}$ = 0.911 S). (C) SV data of the equimolar mixture 9α/9β ($\bar{v}$ = 0.762 cm^3 g^{-1}). Continuous c(s) distribution returned a molecular mass of 9442 Da corresponding to 0.9 x heterodimer mass at 95% confidence level (f/f_0 = 1.151, s = 1.172 S, $s_{20,w}$ = 1.218 S). Conditions: 50 µM total concentration of peptides, PBS buffer, pH 7.4.

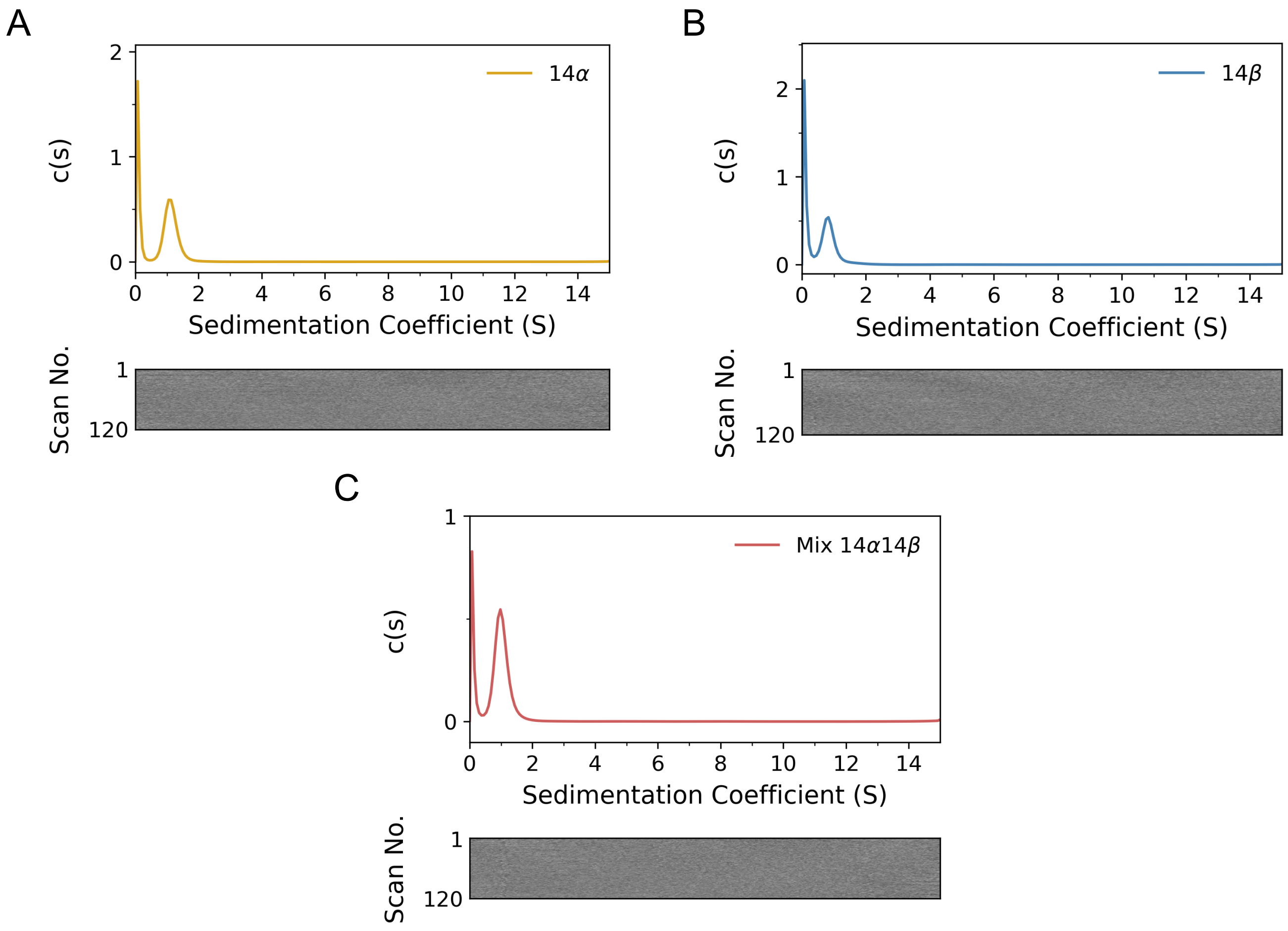

Figure S19. Sedimentation velocity (SV) AUC data for the peptides of pair 14 at 50 krpm. Residuals are shown as a bitmap below the fitted data. (A) SV data of the peptide 14*α* ($\bar{v}$ = 0.757 $cm^3 g^{-1}$). Continuous c(s) distribution returned a molecular mass of 11137 Da corresponding to 2.1 x monomer mass at 95% confidence level (f/f_0 = 1.362, s = 1.132 S, $s_{20,w}$ = 1.176 S). (B) SV data of the peptide 14*β* ($\bar{v}$ = 0.759 $cm^3 g^{-1}$). Continuous c(s) distribution returned a molecular mass of 10335 Da corresponding to 2.0 x monomer mass at 95% confidence level (f/f_0 = 1.660, s = 0.874 S, $s_{20,w}$ = 0.909 S). (C) SV data of the equimolar mixture 14*α*/14*β* ($\bar{v}$ = 0.758 $cm^3 g^{-1}$). Continuous c(s) distribution returned a molecular mass of 9024 Da corresponding to 0.9 x heterodimer mass at 95% confidence level (f/f_0 = 1.304, s = 1.022 S, $s_{20,w}$ = 1.062 S). Conditions: 50 µM total concentration of peptides, PBS buffer, 500 µM TCEP, pH 7.4.

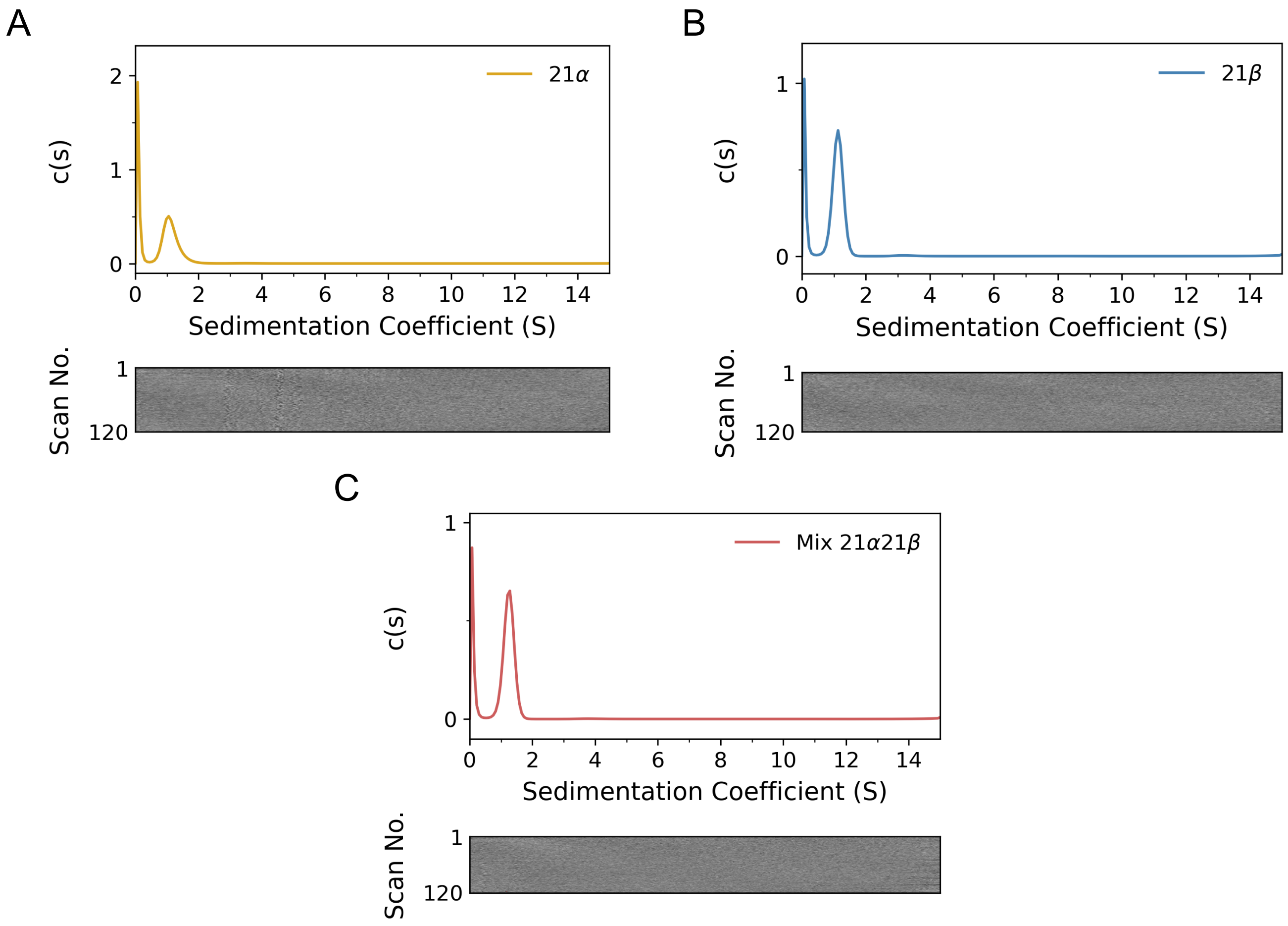

Figure S20. Sedimentation velocity (SV) AUC data for the peptides of pair 21 at 50 krpm. Residuals are shown as a bitmap below the fitted data. (A) SV data of the peptide 21α ($\overline{v}$ = 0.762 cm^3 g^{-1}). Continuous c(s) distribution returned a molecular mass of 12500 Da corresponding to 2.4 x monomer mass at 95% confidence level (f/f_0 = 1.446, s = 1.126 S, $s_{20,w}$ = 1.170 S). (B) SV data of the peptide 21β ($\overline{v}$ = 0.764 cm^3 g^{-1}). Continuous c(s) distribution returned a molecular mass of 11417 Da corresponding to 2.1 x monomer mass at 95% confidence level (f/f_0 = 1.350, s = 1.122 S, $s_{20,w}$ = 1.167 S). (C) SV data of the equimolar mixture 21α/21β ($\overline{v}$ = 0.763 cm^3 g^{-1}). Continuous c(s) distribution returned a molecular mass of 10545 Da corresponding to 1.0 x heterodimer mass at 95% confidence level (f/f_0 = 1.163, s = 1.243 S, $s_{20,w}$ = 1.292 S). Conditions: 50 µM total concentration of peptides, PBS buffer, pH 7.4.

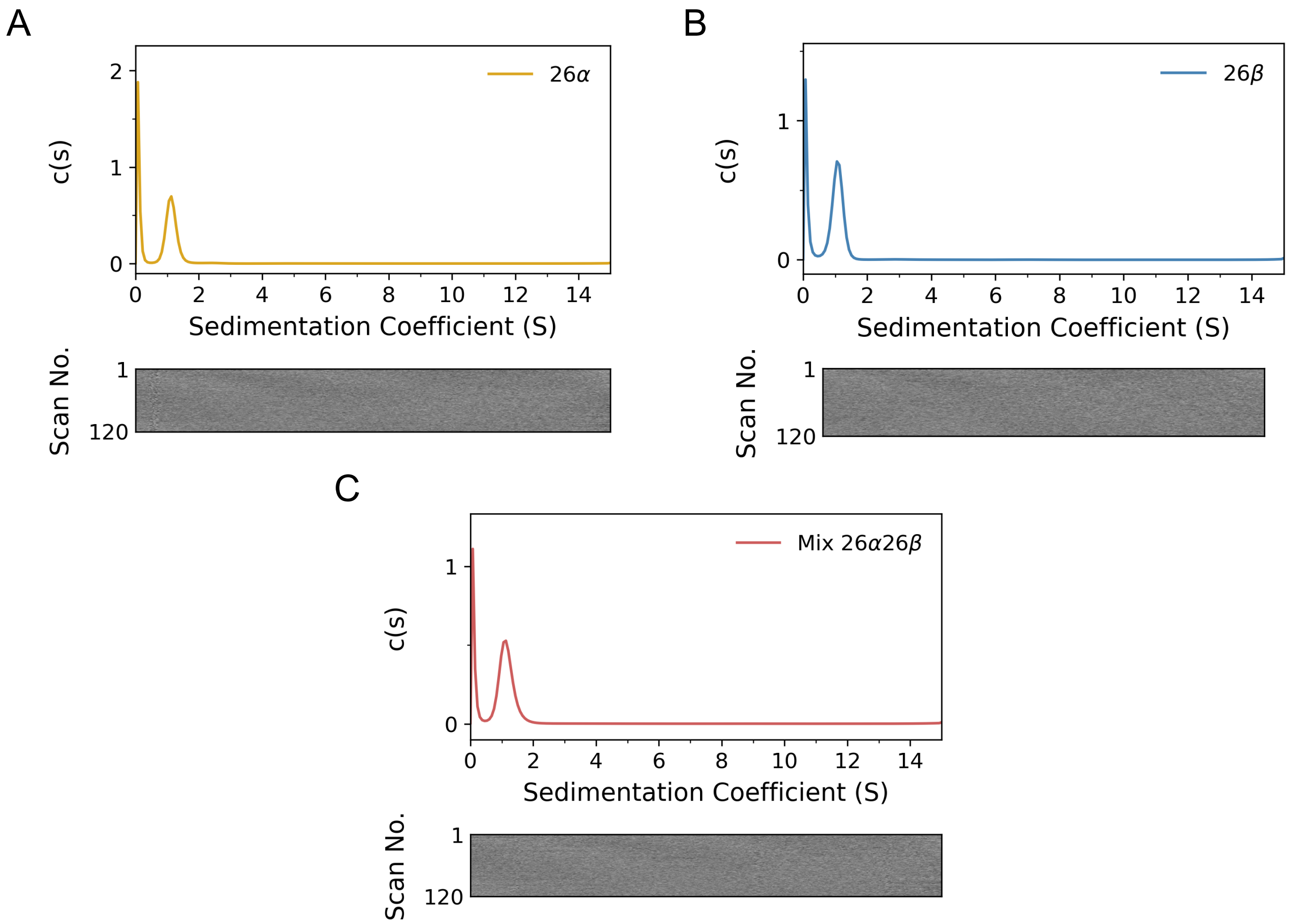

Figure S21. Sedimentation velocity (SV) AUC data for the peptides of pair 26 at 50 krpm. Residuals are shown as a bitmap below the fitted data. (A) SV data of the peptide 26*α* ($\bar{v}$ = 0.765 cm^3 g^{-1}). Continuous c(s) distribution returned a molecular mass of 12209 Da corresponding to 2.3 x monomer mass at 95% confidence level (f/f$_0$ = 1.395, s = 1.132 S, $s_{20,w}$ = 1.177 S). (B) SV data of the peptide 26*β* ($\bar{v}$ = 0.759 cm^3 g^{-1}). Continuous c(s) distribution returned a molecular mass of 11182 Da corresponding to 2.1 x monomer mass at 95% confidence level (f/f$_0$ = 1.429, s = 1.068 S, $s_{20,w}$ = 1.110 S). (C) SV data of the equimolar mixture 26*α*/26*β* ($\bar{v}$ = 0.762 cm^3 g^{-1}). Continuous c(s) distribution returned a molecular mass of 11661 Da corresponding to 1.1 x heterodimer mass at 95% confidence level (f/f$_0$ = 1.350, s = 1.149 S, $s_{20,w}$ = 1.195 S). Conditions: 50 µM total concentration of peptides, PBS buffer, 500 µM TCEP, pH 7.4.

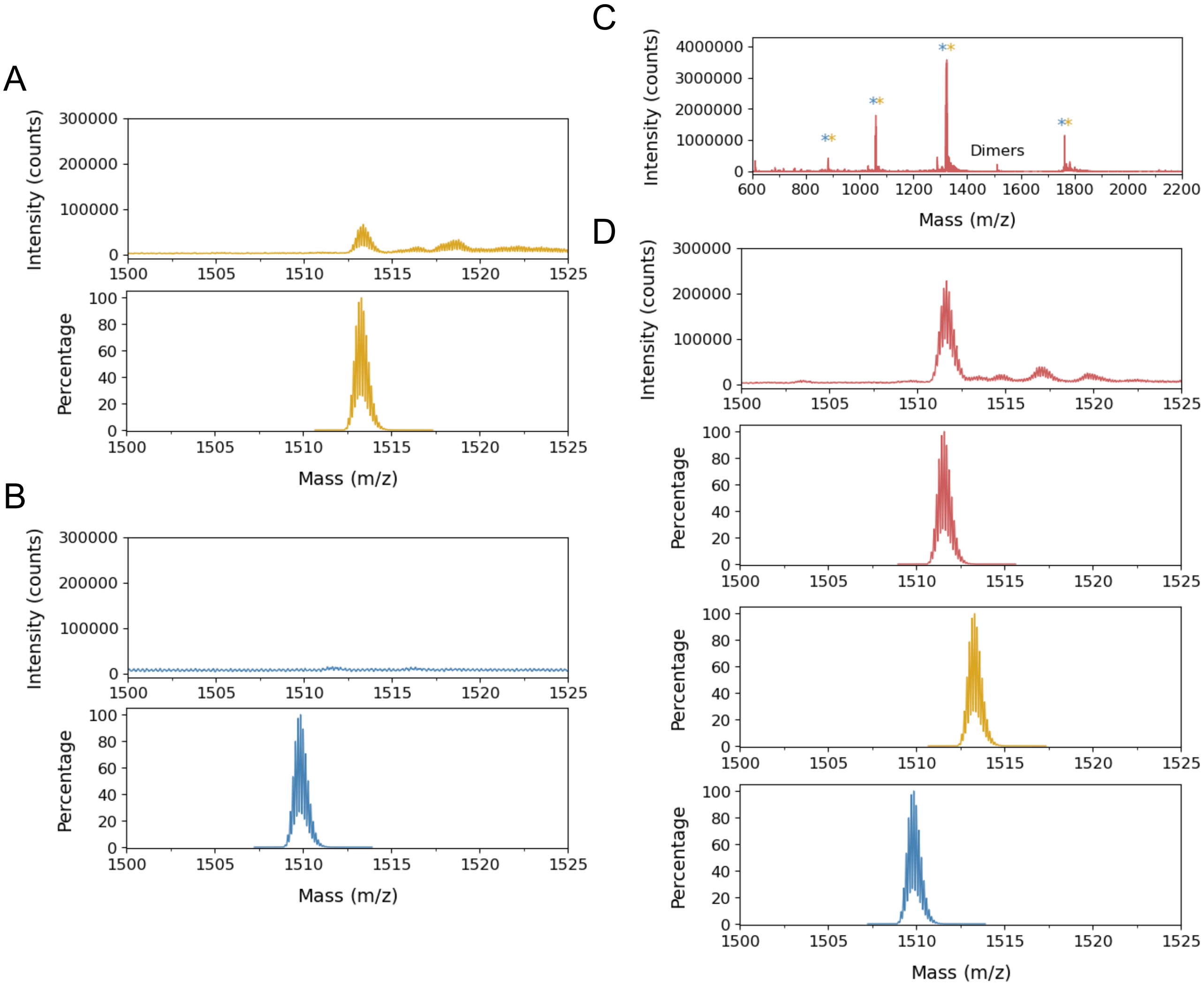

Figure S22. Native mass spectrometry (MS) data for the the peptides of pair 9. Focus on the zoomed area corresponding to the dimeric species protonated with 7 H^+. (A) Experimental native MS data (top panel) vs simulations (bottom panel) of the homodimer 9α protonated with 7 H^+. Formula for the homodimer 9α is $C_{478}H_{802}N_{126}O_{142}$. (B) Experimental native MS data (top panel) vs simulations (bottom panel) of the homodimer 9β protonated with 7 H^+. Formula for the homodimer 9β is $C_{474}H_{794}N_{126}O_{144}$. (C) Mass spectra of the mixture 9α/9β with the monomer 9α represented with yellow stars, the monomer 9β with blue stars. The dimeric region corresponding to a charge state of 7 H^+ is specified. (D) Top panel: zoom corresponding to the dimeric region protonated with 7 H^+ of the mixture 9α/9β. Bottom panels: simulations of the heterodimer 9α/9β protonated with 7 H^+ (red) and simulations of the homodimers 9α and 9β (yellow and blue, respectively). Formula for the heterodimer 9α/9β is $C_{476}H_{798}N_{126}O_{143}$. Conditions: 50 µM total concentration of peptides, ammonium acetate buffer, pH 7.0

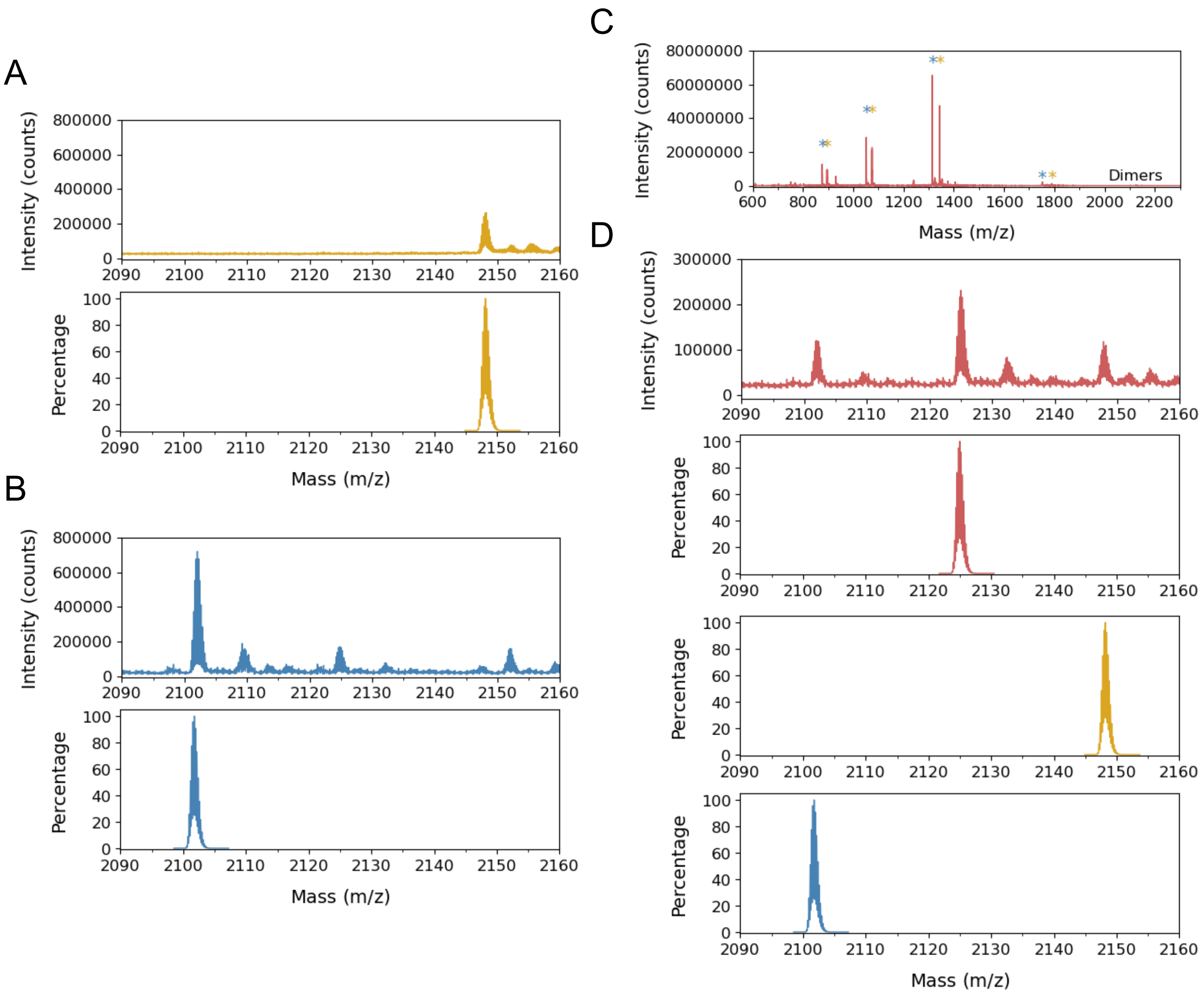

Figure S23. Native mass spectrometry (MS) data for the peptides of pair 14. Focus on the zoomed area corresponding to the dimeric species protonated with 5 H^+. (A) Experimental native MS data (top panel) vs simulations (bottom panel) of the homodimer 14α protonated with 5 H^+. Formula for the homodimer 14α is $C_{478}H_{804}N_{132}O_{142}S_2$. (B) Experimental native MS data (top panel) vs simulations (bottom panel) of the homodimer 14β protonated with 5 H^+. Formula for the homodimer 14β is $C_{470}H_{784}N_{126}O_{140}S_2$. (C) Mass spectra of the mixture 14α/14β with the monomer 14α represented with yellow stars, the monomer 14β with blue stars. The dimeric region corresponding to a charge state of 5 H^+ is specified. (D) Top panel: zoom corresponding to the dimeric region protonated with 5 H^+ of the mixture 14α/14β. Bottom panels: simulations of the heterodimer 14α/14β protonated with 5 H^+ (red) and simulations of the homodimers 14α and 14β (yellow and blue, respectively). Formula for the heterodimer 14α/14β is $C_{474}H_{794}N_{129}O_{141}S_2$. Conditions: 50 µM total concentration of peptides, ammonium acetate buffer, 500 µM TCEP, pH 7.0.

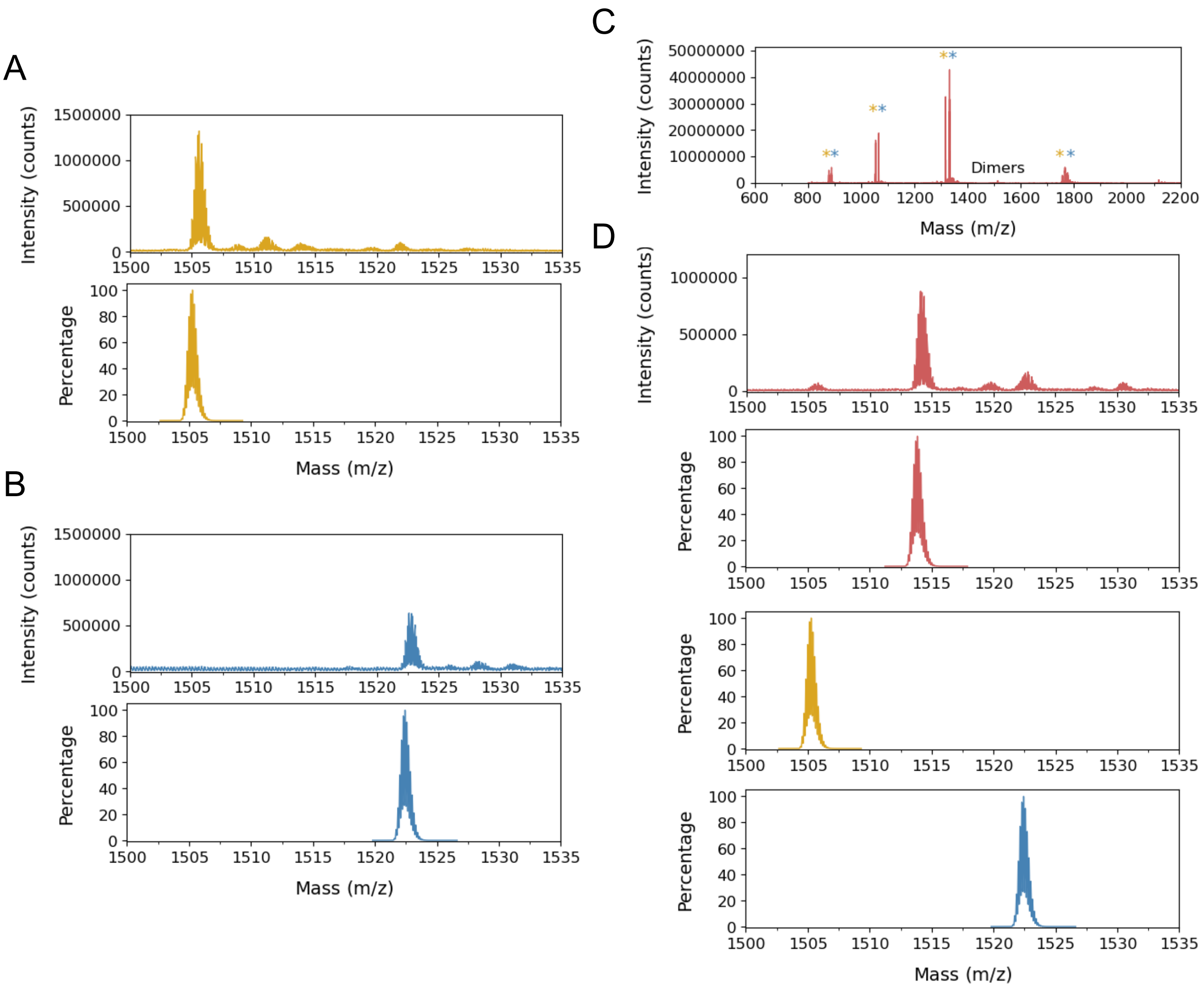

Figure S24. Native mass spectrometry (MS) data for the peptides of pair 21. Focus on the zoomed area corresponding to the dimeric species protonated with 7 H^+. (A) Experimental native MS data (top panel) vs simulations (bottom panel) of the homodimer 21α protonated with 7 H^+. Formula for the homodimer 21α is $C_{474}H_{794}N_{126}O_{142}$. (B) Experimental native MS data (top panel) vs simulations (bottom panel) of the homodimer 21β protonated with 7 H^+. Formula for the homodimer 21β is $C_{486}H_{802}N_{126}O_{140}$. (C) Mass spectra of the mixture 21α/21β with the monomer 21α represented with yellow stars, the monomer 21β with blue stars. The dimeric region corresponding to a charge state of 7 H^+ is specified. (D) Top panel: zoom corresponding to the dimeric region protonated with 7 H^+ of the mixture 21α/21β. Bottom panels: simulations of the heterodimer 21α/21β protonated with 7 H^+ (red) and simulations of the homodimers 21α and 21β (yellow and blue, respectively). Formula for the heterodimer 21α/21β is $C_{480}H_{798}N_{126}O_{141}$. Conditions: 50 µM total concentration of peptides, ammonium acetate buffer, pH 7.0.

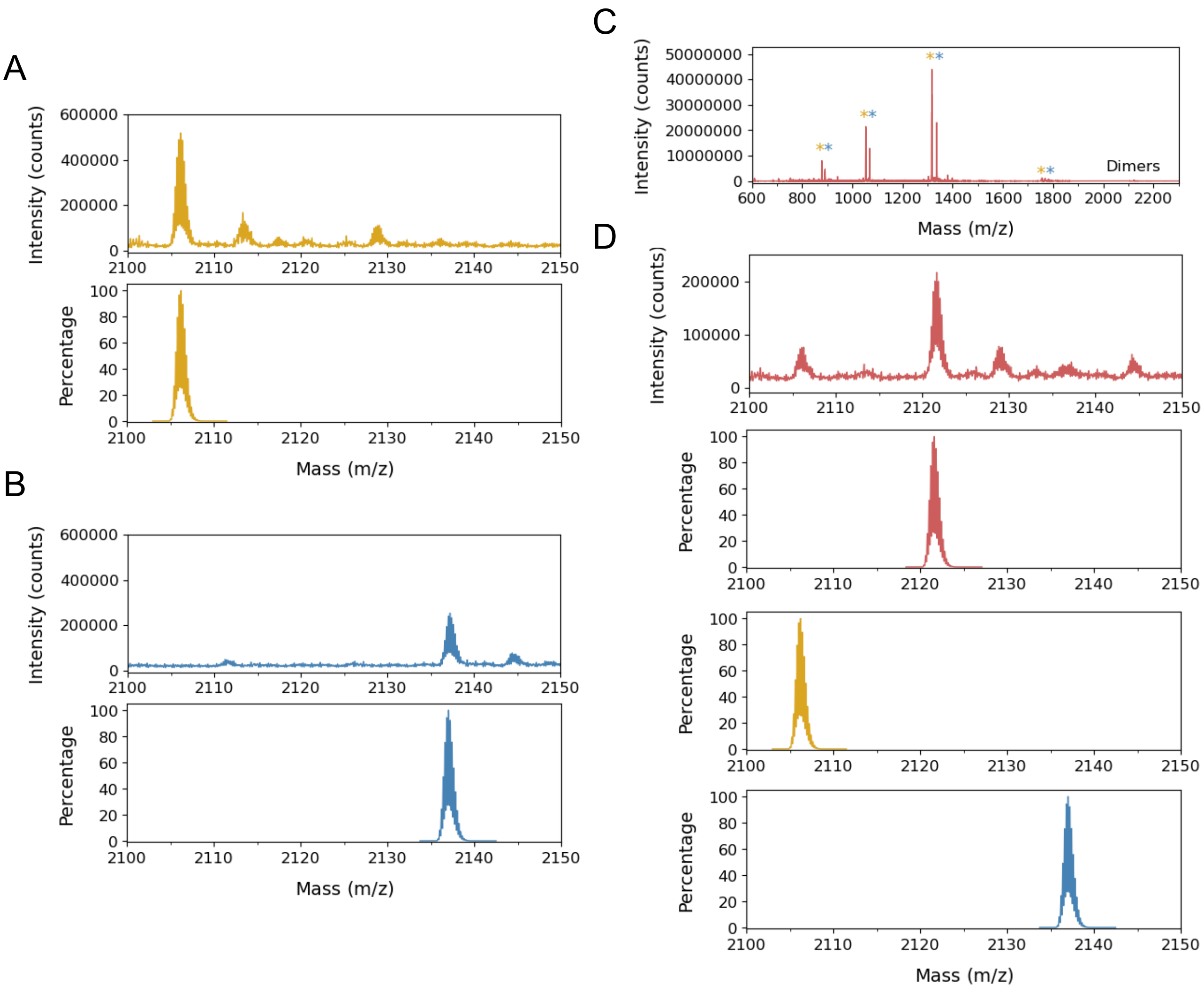

Figure S25. Native mass spectrometry (MS) data for the peptides of pair 26. Focus on the zoomed area corresponding to the dimeric species protonated with 5 H^+. (A) Experimental native MS data (top panel) vs simulations (bottom panel) of the homodimer 26α protonated with 5 H^+. Formula for the homodimer 26α is $C_{476}H_{798}N_{126}O_{140}$. (B) Experimental native MS data (top panel) vs simulations (bottom panel) of the homodimer 26β protonated with 5 H^+. Formula for the homodimer 26β is $C_{478}H_{800}N_{126}O_{144}S_2$. (C) Mass spectra of the mixture 26α/26β with the monomer 26α represented with yellow stars, the monomer 26β with blue stars. The dimeric region corresponding to a charge state of 5 H^+ is specified. (D) Top panel: zoom corresponding to the dimeric region protonated with 5 H^+ of the mixture 26α/26β. Bottom panels: simulations of the heterodimer 26α/26β protonated with 5 H^+ (red) and simulations of the homodimers 26α and 26β (yellow and blue, respectively). Formula for the heterodimer 26α/26β is $C_{477}H_{799}N_{126}O_{142}S$. Conditions: 50 µM total concentration of peptides, ammonium acetate buffer, 500 µM TCEP, pH 7.0.

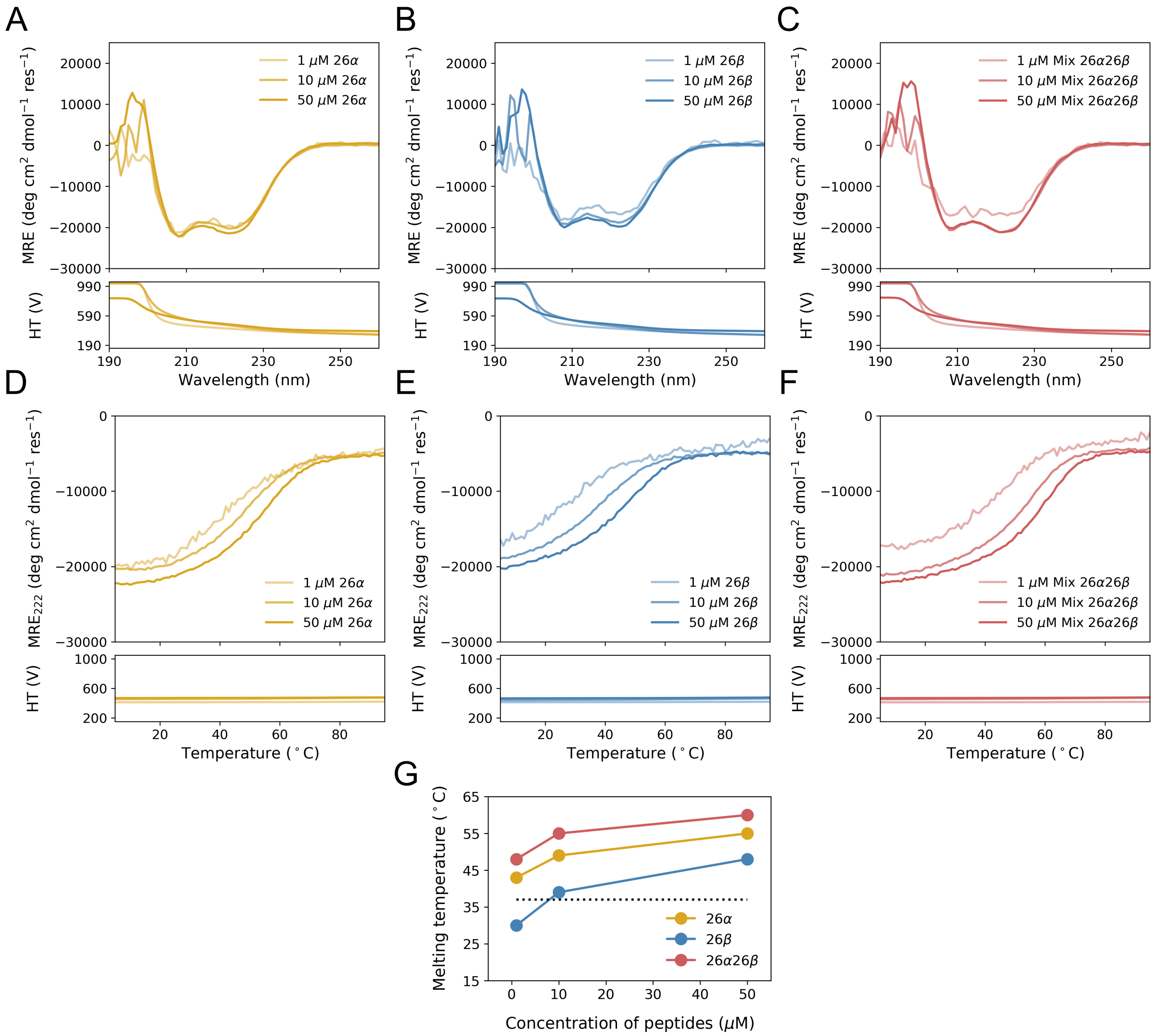

Figure S26. Concentration-dependence CD spectroscopy data for pair 26. (A-C) CD spectra at 5 °C at different peptide concentration (from 1 to 50 µM) for the peptides 26*α* (A) and 26*β* (B) alone and the mixture of both peptides 26*α*/26*β* (C). (D-F) Thermal unfolding monitored at 222 nm wavelength at different peptide concentration (from 1 to 50 µM) for the peptides 26*α* (D) and 26*β* (E) alone and the mixture of both peptides 26*α*/26*β* (F). (G) Deduced second derivatives of melting curves for the peptides 26*α* (yellow) and 26*β* (blue) and the mixture of both peptides 26*α*/26*β* (red) at the different concentrations. The temperature used for in-cell experiment (i.e., 37 ºC) is represented in dotted black line. Conditions: 1-50 µM total concentration of peptides, PBS buffer, 10 equiv. TCEP, pH 7.4.

2.2 Supplementary Tables

	CC-HP1.0	26α/26β
**PDB ID**	8BCS	8BCT
**Data collection**		
Source	Diamond I04	Diamond I04
Detector	EIGER2 XE 16M	EIGER2 XE 16M
Wavelength (Å)	0.9795	0.9795
Resolution range	31.56 - 2.1 (2.175 - 2.1)	35.77 - 1.7 (1.761 - 1.7)
Space group	P 3_1 2 1	C 1 2 1
Unit cell: *a b c (Å)*	36.4379 36.4379 59.3288	86.8928 78.3222 81.5499
α, β, γ (°)	90 90 120	90 107.966 90
Total reflections	51275 (4071)	396218 (39630)
Unique reflections	2896 (284)	57177 (5660)
Multiplicity	17.7 (14.3)	6.9 (7.0)
Completeness (%)	99.97 (99.65)	99.86 (99.89)
Mean I/sigma(I)	31.45 (3.00)	18.51 (0.93)
Wilson B-factor	58.89	29.22
R-merge	0.03629 (0.7703)	0.04752 (0.9934)
R-meas	0.03743 (0.7987)	0.05137 (1.074)
R-pim	0.009034 (0.2081)	0.01938 (0.4045)
CC1/2	1 (0.91)	0.999 (0.792)
CC*	1 (0.976)	1 (0.94)
**Refinement**		
Reflections used in refinement	2895 (283)	57105 (5654)
Reflections used for R-free	152 (16)	2921 (303)
R-work	0.2394 (0.3541)	0.2152 (0.3656)
R-free	0.2672 (0.4607)	0.2225 (0.3796)
CC(work)	0.968 (0.732)	0.962 (0.792)
CC(free)	0.953 (0.179)	0.975 (0.769)
Number of non-hydrogen atoms	330	3252
macromolecules	326	2945
ligands	4	88
solvent	-	219
Protein residues	49	391
RMS(bonds)	0.008	0.012
RMS(angles)	0.99	1.68
Ramachandran favored (%)	100.00	99.73
Ramachandran allowed (%)	0.00	0.27
Ramachandran outliers (%)	0.00	0.00
Rotamer outliers (%)	4.55	1.06
Clashscore	4.61	5.78
Average B-factor	67.81	37.67
macromolecules	67.76	36.76
ligands	71.96	51.63
solvent	-	44.30
Number of TLS groups	1	8

Table S1. Merging and refinement statistics for all X-ray crystal structures.

Peptide name	Sequence<br>gabcdef gabcdef gabcdef gabcdef gabcdef gabcdef	Calc. mass [M]	Obs. Mass [M]
9α	Ac-G ELEAL**A**K K**L**KAL**G**W K**I**KALSK EPSAQ ELEAL**G**Q E**S**EAL**T**K K**F**KALAQ G-NH_2	5293.2	5294.1
9β	Ac-G ELEAL**G**K K**S**KAL**T**W K**V**KALSK EPSAQ ELEAL**S**Q E**F**EAL**G**K K**V**KALAQ G-NH_2	5281.2	5281.8
14α	Ac-G ELEAL**A**K K**R**KAL**G**W K**F**KALSK EPSAQ ELEAL**S**Q E**C**EAL**S**K K**I**KALAQ G-NH_2	5368.3	5368.4
14β	Ac-G ELEAL**G**K K**C**KAL**A**W K**F**KALSK EPSAQ ELEAL**T**Q E**V**EAL**A**K K**G**KALAQ G-NH_2	5252.2	5253.0
21α	Ac-G ELEAL**G**K K**A**KAL**T**W K**L**KALSK EPSAQ ELEAL**G**Q E**T**EAL**A**K K**F**KALAQ G-NH_2	5265.2	5265.2
21β	Ac-G ELEAL**A**K K**F**KAL**A**W K**V**KALSK EPSAQ ELEAL**T**Q E**F**EAL**A**K K**A**KALAQ G-NH_2	5325.3	5325.3
26α	Ac-G ELEAL**G**K K**F**KAL**A**W K**V**KALSK EPSAQ ELEAL**T**Q E**A**EAL**G**K K**I**KALAQ G-NH_2	5263.2	5263.0
26β	Ac-G ELEAL**A**K K**T**KAL**T**W K**F**KALSK EPSAQ ELEAL**T**Q E**C**EAL**G**K K**L**KALAQ G-NH_2	5340.3	5340.9
26β-c4CF	Ac-G ELEAL**A**K K**T**KAL**T**W K**F**KALSK EPSAQ ELEAL**T**Q E**C**EAL**G**K K**LX**ALAQ G-NH_2	5327.2	5327.3
26β-n4CF	Ac-G ELEAL**A**K K**T**KAL**T**W K**FX**ALSK EPSAQ ELEAL**T**Q E**C**EAL**G**K K**L**KALAQ G-NH_2	5327.2	5326.2
26α-nMSE	Ac-G EL**Z**AL**G**K K**F**KAL**A**W K**V**KALSK EPSAQ ELEAL**T**Q E**A**EAL**G**K K**I**KALAQ G-NH_2	5254.1	5254.1

Table S2. Sequences of the synthesized peptides for *in vitro* studies with the associated mass.

X = 4-cyanophelalanine (4CF); Z = selenomethionine (MSE)

PDB ID	*Syn* or *anti*	Structure is a helix-loop-helix dimer	Structure part of a larger protein	*De novo* protein
1EC5	*anti*	✓		✓
1F4M	*syn*	✓		
1PD3	*syn*	✓		
1RPR	*anti*	✓		
1SKV	*anti*	✓		
1VMG	*anti*		✓	
2VOX	*syn*		✓	
3A6M	*syn*		✓	
3G67, 3G6B	*syn*	✓		
3ZX6	*syn*		✓	
4ADZ	*anti*		✓	
4BIU, 4BIW, 4CB0	*syn*		✓	
4JAU, 4JAV	*syn*		✓	
4U7N	*syn*		✓	
5FMN	*anti*		✓	
5IUN	*syn*		✓	
5J0K, 5J10	*syn*	✓		✓
5LBM	*syn* and *anti*		✓	
5LFK	*syn*		✓	
6B87	*syn*	✓		✓
6DMP	*syn*	✓		✓
6H8F	*anti*	✓		
**Total *syn***	**15 (65%)**	**6 (60%)**	**9 (69%)**	**3 (75%)**
**Total *anti***	**8 (35%)**	**4 (40%)**	**4 (31%)**	**1 (25%)**

Table S3. Comparison of *syn* or *anti* conformations within structures containing 4-helix coiled coils. Structurally resolved helix-loop-helix dimers with knobs-into-holes packing identified by SOCKET2[20] were visually inspected. These were predominantly obtained from the CC+ database.[21] Structures were either composed entirely of a helix-loop-helix dimer or the helix-loop-helix dimers were part of a larger protein (e.g., oligomerization domains within a protein). Helix-loop-helix dimeric domains without full structural characterization were not considered.

SUPPLEMENTARY REFERENCES

1. Hu, C. D.; Chinenov, Y.; Kerppola, T. K., Visualization of interactions among bZIP and Rel family proteins in living cells using bimolecular fluorescence complementation. *Mol Cell* **2002,** *9* (4), 789-98.
2. Smith, A. J.; Thomas, F.; Shoemark, D.; Woolfson, D. N.; Savery, N. J., Guiding Biomolecular Interactions in Cells Using de Novo Protein-Protein Interfaces. *ACS Synth Biol* **2019,** *8* (6), 1284-1293.
3. Bernhardt, T. G.; de Boer, P. A., The Escherichia coli amidase AmiC is a periplasmic septal ring component exported via the twin-arginine transport pathway. *Mol Microbiol* **2003,** *48* (5), 1171-82.
4. Kuipers, B. J.; Gruppen, H., Prediction of molar extinction coefficients of proteins and peptides using UV absorption of the constituent amino acids at 214 nm to enable quantitative reverse phase high-performance liquid chromatography-mass spectrometry analysis. *J Agric Food Chem* **2007,** *55* (14), 5445-51.
5. Schuck, P., Size-distribution analysis of macromolecules by sedimentation velocity ultracentrifugation and lamm equation modeling. *Biophys J* **2000,** *78* (3), 1606-19.
6. Schuck, P., On the analysis of protein self-association by sedimentation velocity analytical ultracentrifugation. *Anal Biochem* **2003,** *320* (1), 104-124.
7. Winter, G., xia2: an expert system for macromolecular crystallography data reduction. *J Appl Crystallogr* **2010,** *43*, 186-190.
8. Winter, G.; Waterman, D. G.; Parkhurst, J. M.; Brewster, A. S.; Gildea, R. J.; Gerstel, M.; Fuentes-Montero, L.; Vollmar, M.; Michels-Clark, T.; Young, I. D.; Sauter, N. K.; Evans, G., DIALS: implementation and evaluation of a new integration package. *Acta Crystallogr D Struct Biol* **2018,** *74* (Pt 2), 85-97.
9. Evans, P. R.; Murshudov, G. N., How good are my data and what is the resolution? *Acta Crystallogr D Biol Crystallogr* **2013,** *69* (Pt 7), 1204-14.
10. Winn, M. D.; Ballard, C. C.; Cowtan, K. D.; Dodson, E. J.; Emsley, P.; Evans, P. R.; Keegan, R. M.; Krissinel, E. B.; Leslie, A. G.; McCoy, A.; McNicholas, S. J.; Murshudov, G. N.; Pannu, N. S.; Potterton, E. A.; Powell, H. R.; Read, R. J.; Vagin, A.; Wilson, K. S., Overview of the CCP4 suite and current developments. *Acta Crystallogr D Biol Crystallogr* **2011,** *67* (Pt 4), 235-42.
11. Rhys, G. G.; Wood, C. W.; Beesley, J. L.; Zaccai, N. R.; Burton, A. J.; Brady, R. L.; Thomson, A. R.; Woolfson, D. N., Navigating the Structural Landscape of De Novo alpha-Helical Bundles. *J Am Chem Soc* **2019,** *141* (22), 8787-8797.
12. McCoy, A. J.; Grosse-Kunstleve, R. W.; Adams, P. D.; Winn, M. D.; Storoni, L. C.; Read, R. J., Phaser crystallographic software. *J Appl Crystallogr* **2007,** *40* (Pt 4), 658-674.
13. Afonine, P. V.; Grosse-Kunstleve, R. W.; Echols, N.; Headd, J. J.; Moriarty, N. W.; Mustyakimov, M.; Terwilliger, T. C.; Urzhumtsev, A.; Zwart, P. H.; Adams, P. D., Towards automated crystallographic structure refinement with phenix.refine. *Acta Crystallogr D Biol Crystallogr* **2012,** *68* (Pt 4), 352-67.
14. Murshudov, G. N.; Skubak, P.; Lebedev, A. A.; Pannu, N. S.; Steiner, R. A.; Nicholls, R. A.; Winn, M. D.; Long, F.; Vagin, A. A., REFMAC5 for the refinement of macromolecular crystal structures. *Acta Crystallogr D Biol Crystallogr* **2011,** *67* (Pt 4), 355-67.
15. Jumper, J.; Evans, R.; Pritzel, A.; Green, T.; Figurnov, M.; Ronneberger, O.; Tunyasuvunakool, K.; Bates, R.; Zidek, A.; Potapenko, A.; Bridgland, A.; Meyer, C.; Kohl, S. A. A.; Ballard, A. J.; Cowie, A.; Romera-Paredes, B.; Nikolov, S.; Jain, R.;

Adler, J.; Back, T.; Petersen, S.; Reiman, D.; Clancy, E.; Zielinski, M.; Steinegger, M.; Pacholska, M.; Berghammer, T.; Bodenstein, S.; Silver, D.; Vinyals, O.; Senior, A. W.; Kavukcuoglu, K.; Kohli, P.; Hassabis, D., Highly accurate protein structure prediction with AlphaFold. *Nature* **2021,** *596* (7873), 583-589.
16. Evans, R.; O'Neill, M.; Pritzel, A.; Antropova, N.; Senior, A.; Green, T.; Žídek, A.; Bates, R.; Blackwell, S.; Yim, J.; Ronneberger, O.; Bodenstein, S.; Zielinski, M.; Bridgland, A.; Potapenko, A.; Cowie, A.; Tunyasuvunakool, K.; Jain, R.; Clancy, E.; Kohli, P.; Jumper, J.; Hassabis, D., Protein complex prediction with AlphaFold-Multimer. *bioRxiv* **2021**, 2021.10.04.463034.
17. Mirdita, M.; Schutze, K.; Moriwaki, Y.; Heo, L.; Ovchinnikov, S.; Steinegger, M., ColabFold: making protein folding accessible to all. *Nat Methods* **2022,** *19* (6), 679-682.
18. Mariani, V.; Biasini, M.; Barbato, A.; Schwede, T., lDDT: a local superposition-free score for comparing protein structures and models using distance difference tests. *Bioinformatics* **2013,** *29* (21), 2722-8.
19. Wood, C. W.; Ibarra, A. A.; Bartlett, G. J.; Wilson, A. J.; Woolfson, D. N.; Sessions, R. B., BAlaS: fast, interactive and accessible computational alanine-scanning using BudeAlaScan. *Bioinformatics* **2020,** *36* (9), 2917-2919.
20. Kumar, P.; Woolfson, D. N., Socket2: A Program for Locating, Visualising, and Analysing Coiled-coil Interfaces in Protein Structures. *Bioinformatics* **2021**.
21. Testa, O. D.; Moutevelis, E.; Woolfson, D. N., CC+: a relational database of coiled-coil structures. *Nucleic Acids Res* **2009,** *37* (Database issue), D315-22.
